# The burden of inflammatory bowel disease in Asia (1990–2023) and projections to 2050: A comprehensive analysis of the 2023 global burden of disease study

**DOI:** 10.1371/journal.pone.0359856

**Published:** 2026-10-08

**Authors:** Yang-Tao Chen, Zhao-Chu Wang, Hui-Min Liu, Chen-Zi Zhao, Ya-Meng Xie, Xun Wang, Hong-Ling Huang, Xiao-Qiang Wu, Jing Wang, Rong Shi, Juan Huang

**Affiliations:** 1 Department of Anorectal surgery, The Affiliated People’s Hospital of Fujian University of Traditional Chinese Medicine, Fuzhou, Fujian, China; 2 Xiamen Medical College Affiliated Haicang Hospital, Xiamen, Fujian, China; Charite Universitatsmedizin Berlin, GERMANY

## Abstract

**Background:**

Inflammatory bowel disease (IBD), including ulcerative colitis (UC) and Crohn’s disease (CD), is becoming an increasingly important public health concern in Asia, with rising incidence and prevalence reported across the region. Despite progress in global burden studies, data gaps remain in Asia due to variations in healthcare and socio-demographic factors. This study aims to address these gaps by assessing the burden of IBD across Asia from 1990 to 2023, focusing on regional trends in UC and CD.

**Method:**

We utilized data from the Global Burden of Diseases, Injuries, and Risk Factors Study (GBD) 2023, focusing on the incidence, disability-adjusted life years (DALYs), prevalence, and mortality of IBD, UC, and CD across 47 Asian countries, which were categorized into five sub-regions: East Asia, Southeast Asia, South Asia, Central Asia, and West Asia. Temporal trends in age-standardized incidence, prevalence, DALY, and mortality rates for IBD, UC, and CD were analyzed by age, sex, and Socio-Demographic Index (SDI), calculating the estimated annual percentage changes(EAPC). Non-seasonal autoregressive integrated moving average (ARIMA) models selected using the corrected Akaike information criterion (AICc) were used to project age-standardized incidence, prevalence, and mortality rates through 2050. Out-of-sample validation was performed using 1990–2019 as the training period and 2020–2023 as the validation period.

**Outcome:**

In 2023, Asia recorded 160,998 new cases of IBD (age-standardized incidence rate: 3.42 per 100,000), with a total of 568,342 DALYs (age-standardized DALY rate: 12.08 per 100,000). The prevalence of IBD reached 1,395,394 cases (age-standardized prevalence rate: 29.69 per 100,000), and there were 13,905 deaths (age-standardized mortality rate: 0.30 per 100,000). From 1990 to 2023, the age-standardized incidence and prevalence rates of IBD increased by 1.95% annually, while the age-standardized DALY rate decreased by 0.19% annually. The age-standardized mortality rate remained broadly stable, with a slight annual decrease of 0.13%. Higher SDI was associated with a higher age-standardized prevalence rate, whereas associations with incidence, DALYs, and mortality were not significant after multiple-testing correction. The projections suggested continued increases in incidence and prevalence through 2050, whereas mortality rates remained broadly stable. Sensitivity analyses supported the robustness of the increasing incidence and prevalence trends, while DALY- and mortality-related findings were less stable. Out-of-sample validation suggested that the ARIMA models generally reproduced the broad direction of recent trends, although prediction performance varied across measures.

**Conclusion:**

From 1990 to 2023, the incidence and prevalence of IBD in Asia have risen significantly, which may result in considerable social and economic challenges for healthcare systems and governments in the future. These findings emphasize the importance of policy makers adopting strategies to address the increasing burden of IBD, such as training specialized healthcare professionals to manage this complex disease.

## Introduction

Inflammatory bowel disease (IBD), encompassing Crohn’s disease (CD) and ulcerative colitis (UC), is a chronic, immune-mediated inflammatory disorder of the gastrointestinal tract that can lead to significant morbidity and long-term complications. Historically regarded as a disorder primarily of Western, high-income countries, IBD has in recent decades undergone an epidemiologic transition: increasing incidence, prevalence, and burden have been documented in newly industrialized and middle-income regions [1]. For example, global analyses have demonstrated that by 2019 there were approximately 4.9 million prevalent IBD cases worldwide, and while some age-standardized rates (prevalence, deaths, DALYs) have declined in certain regions between 1990 and 2019, many regions have shown increasing absolute numbers and some rising rates [2,3].

Large-scale burden of disease studies, notably those from the Global Burden of Disease Study (GBD) Collaborators, have provided critical metrics such as incidence, prevalence, mortality (deaths), and disability-adjusted life years (DALYs) for IBD at global, regional, and national levels over time [4–6]. These metrics enable policymakers and health-system planners to quantify and compare the evolving burden of IBD across populations and to prioritize resources accordingly.

Nevertheless, despite these advances, major gaps persist—especially in Asia. Asia’s vast and heterogeneous population spans a wide spectrum of socio-demographic development, healthcare infrastructure, and environmental exposures, yet many studies remain country-specific or hospital-based, and comprehensive regional breakdowns remain scarce [7,8]. For example, a recent narrative review underlined that although the incidence and prevalence of IBD in Asia have increased markedly over the past two decades, detailed data remain limited and uneven across subregions (East, Southeast, South, West, Central Asia) with little consistent subphenotype separation (CD vs UC) or full burden metrics (incidence, prevalence, deaths, DALYs) over time [9]. Moreover, while regional studies exist (e.g., in India, China, Japan, Southeast Asia), few provide uniform long-term trend analyses up to recent years, and many do not fully utilize the latest GBD-style metrics across all IBD phenotypes [10,11].

To address these gaps, our study offers a novel and comprehensive assessment of IBD burden across Asia between 1990 and 2023. We subdivide Asia into five meaningful regions—East Asia, South Asia, Southeast Asia, Central Asia, and West Asia—and separately examine CD and UC. Using incidence, prevalence, deaths, and DALYs as outcome measures, we aim to map long-term trends, identify emerging hotspots, and elucidate regional heterogeneity in the burden of IBD. Importantly, inclusion of data through 2023 constitutes a significant advancement over many prior analyses, and allows us to capture the most up-to-date epidemiologic shifts in the region. By comparing trends across subregions, and by distinguishing CD versus UC, our goal is to provide actionable insights for health-system planning, resource allocation, and future research priorities tailored to the Asian context.

## Method

### Data collection

We obtained data on the burden of IBD, UC and CD, from GBD 2023, using the Global Health Data Exchange (GHDx) results tool. The GBD 2023 study provides comprehensive estimates on the burden of 369 diseases and injuries across 204 countries and territories from 1990 to 2023, offering a standardized approach to measure health outcomes globally [12]. For this study, we specifically extracted data from GBD 2023 on the incidence, prevalence, deaths, and DALYs related to IBD, UC, and CD across the Asian region.

The data we utilized from GBD 2023 were derived from a combination of vital registration systems, national health surveys, disease registries, and other publicly available health datasets, with statistical modeling techniques employed to fill gaps in the data. This robust approach enables accurate, comparable estimates of disease burden across regions and over time [13].

For the purposes of this study, we focused on data for 47 countries in Asia, which were classified into five distinct regions based on GBD’s regional definition: East Asia, Southeast Asia, South Asia, Central Asia, and West Asia. The countries included in each region are as follows: East Asia: China, Mongolia, Japan, Republic of Korea, Democratic People’s Republic of Korea; Southeast Asia: Brunei Darussalam, Cambodia, Indonesia, Lao People’s Democratic Republic, Malaysia, Myanmar, Philippines, Singapore, Thailand, Timor-Leste, Viet Nam; South Asia: Afghanistan, Bangladesh, Bhutan, India, Maldives, Nepal, Pakistan, Sri Lanka; Central Asia: Kazakhstan, Kyrgyzstan, Tajikistan, Turkmenistan, Uzbekistan; West Asia: Armenia, Azerbaijan, Bahrain, Cyprus, Georgia, Iran (Islamic Republic of), Iraq, Israel, Jordan, Kuwait, Lebanon, Oman, Palestine, Qatar, Saudi Arabia, Syrian Arab Republic, United Arab Emirates, Türkiye.

In addition to the disease-specific data, we also downloaded information on the Socio-Demographic Index (SDI) and population estimates for each Asian country from 1990 to 2023. These data were used to explore associations between social and economic factors and disease burden. The SDI, a composite measure reflecting income, education, and fertility rates, is a key factor in understanding regional health trends and disparities [14]. Population data were essential for age-standardizing disease burden estimates, ensuring comparability across regions and time periods [15].

The availability and quality of primary IBD data varied substantially across Asian countries. In countries with limited population-based surveillance, disease registries, vital registration, or access to endoscopic diagnosis, the GBD estimates relied more heavily on statistical modelling, covariates, and information borrowed across locations, age groups, sexes, and years. Consequently, estimates for data-sparse settings may be more sensitive to model assumptions and may have wider uncertainty intervals than estimates for countries with more complete surveillance systems. We therefore interpreted all country- and regional-level estimates together with their 95% uncertainty intervals (UIs).

### Data estimate

In this study, we obtained age-standardized rates (ASRs) per 100,000 population for IBD, including UC and CD, from the GBD 2023. These rates were extracted from GBD for individual countries and regions, and the ASRs were calculated using the following formula:


$$\begin{array}{c} ASR=\frac{\sum_{i=\text{1}}^{n}w_{i}\cdot {Rate}_{i}}{\sum_{i=\text{1}}^{n}w_{i}} \end{array}$$

Where:

- ASRis the age-standardized rate per 100,000 population.
- $w_{i}$is the weight of each age group in the standard population (e.g., the World Health Organization World Standard Population).
- ${\text{Rate}}_{i}$is the disease rate for each age group i.
- n represents the total number of age groups considered [16].

For regional analyses, however, data for the broader regions of Asia and its sub-regions (East Asia, Southeast Asia, South Asia, Central Asia, and West Asia) could not be directly obtained from the GBD database. To compute these regional estimates, we first downloaded the population data for each of the 47 countries in Asia for the period from 1990 to 2023. Using this population data, we calculated the population weights for each country by dividing the population of each country in each year by the total population of the corresponding region. These weights were used to derive the regional estimates for incidence, prevalence, deaths, and DALYs, as well as for uncertainty intervals (UI), across different age groups and genders.

In addition to disease-specific data, we also calculated the SDI for each region based on the same population data. The SDI was calculated for each region using the population-weighted average SDI of the countries within the region. This approach enabled us to explore the relationship between socio-economic factors and the burden of IBD in the Asian context.

Furthermore, we calculated the annual percentage change (EAPC) for the four key indicators—incidence, prevalence, deaths, and DALYs—from 1990 to 2023. The EAPC measures the average annual rate of change in the disease burden over the study period, expressed as a percentage. A positive EAPC indicates an increasing trend in disease burden, while a negative EAPC indicates a decrease. To estimate the EAPC, we assumed that the natural logarithm of the age-standardized rate fits a linear regression model, where the model is represented as y=α+βx+ϵ, with y being the natural log of the age-standardized rate and xreferring to the calendar year. The estimated EAPC was then calculated as $\text{1}\text{0}\text{0}\times (e^{\beta }-\text{1})$, where β is the slope of the regression line. Additionally, we computed the 95% confidence intervals (CIs) for each EAPC to assess the uncertainty of the observed trends. A significant EAPC greater than 0 suggests an upward trend in disease burden, while a value less than 0 indicates a decline [17]. This methodology enabled us to evaluate the temporal trends in the burden of IBD, UC, and CD across Asia and its sub-regions over the past three decades.

### Statistical analysis

Descriptive analyses were conducted for incidence, prevalence, deaths, and DALYs of IBD, UC, and CD in Asia and its subregions by year, sex, and disease subtype. Temporal trends from 1990 to 2023 were assessed using EAPCs in age-standardized rates. Age- and sex-specific patterns were described using stratified analyses of burden measures across age groups and between males and females. To further explore sex-related differences, we estimated sex-specific age-standardized incidence, prevalence, DALY, and mortality rates for IBD, UC, and CD across Asia and its five subregions in 2023. Male-to-female ratios were calculated for each region, disease subtype, and burden measure. Temporal trends in UC and CD were compared using log-linear models containing an interaction term between calendar year and disease subtype, with Benjamini–Hochberg correction applied across region–measure comparisons. The consistency of the apparent bimodal CD prevalence pattern was assessed descriptively using age-specific prevalence rates in 2023 for Asia and its five subregions. Local maxima were defined as rates exceeding those in both immediately adjacent age groups.

Country-level associations between SDI and age-standardized IBD burden measures in 2023 were examined using separate ecological linear regression models, with SDI as the sole explanatory variable and no additional covariates. Effect estimates were reported per 0.1-unit increase in SDI together with 95% CIs, P values, R^2^, adjusted R^2^, and AIC. Potential non-linearity was assessed using natural cubic splines with three degrees of freedom. P values were corrected using the Benjamini–Hochberg false discovery rate procedure. Pearson and Spearman correlations and exclusion analyses were performed as sensitivity analyses.

Future age-standardized incidence, prevalence, and mortality rates for IBD, UC, and CD in Asia were projected from 2024 to 2050 using separate non-seasonal ARIMA models fitted to annual data from 1990 to 2023 [18,19]. Models were selected using the auto.arima() function by minimizing AICc, with stepwise = FALSE and approximation = FALSE. Model adequacy was assessed using residual plots, residual autocorrelation functions, and the Ljung–Box test. Out-of-sample validation was conducted using 1990–2019 as the training period and 2020–2023 as the validation period, with performance evaluated using MAE, RMSE, MAPE, and coverage of the 95% ARIMA prediction intervals. Final models were refitted using the complete 1990–2023 point-estimate series to generate projections through 2050 [20]. The reported 95% prediction intervals represent uncertainty arising from the ARIMA forecasting models, conditional on the historical GBD point estimates. The GBD lower and upper uncertainty bounds were not propagated through the forecasting models; therefore, these intervals do not represent GBD uncertainty or combined GBD and forecasting uncertainty.

To evaluate the influence of data uncertainty, relative uncertainty was calculated as \((upper\ UI-lower\ UI)/point\ estimate\). For each burden measure, countries in the highest quartile of relative uncertainty in 2023 were classified as high-uncertainty countries. We repeated the principal country-level SDI analyses after excluding these countries. Because Afghanistan, Myanmar, and the Lao People’s Democratic Republic were specifically considered settings with potentially limited health-data infrastructure, an additional sensitivity analysis excluded these three countries. Temporal EAPC analyses were also repeated using the lower and upper bounds of the GBD 95% UIs to determine whether the direction of each trend was consistent across the uncertainty range. These sensitivity analyses assessed the robustness of the findings to the uncertainty reported in the GBD estimates; they did not quantify all potential sources of structural model uncertainty or diagnostic misclassification. These analyses were intended to evaluate the sensitivity of the principal conclusions rather than to validate the accuracy of individual country estimates.

All statistical analysis and plotting were performed using R version 4.4.3.

## Results

In 2023, Asia recorded a total of 160997.546 new cases of IBD, corresponding to an age-standardized incidence rate of 3.423 per 100,000 population (Table 1). The total number of DALYs for IBD in Asia was 568341.629, with an age-standardized DALY rate of 12.078 per 100,000 population(Table 1). Meanwhile, the total prevalence of IBD in Asia reached 1395394.162, with an age-standardized prevalence rate of 29.691 per 100,000 population. Additionally, the total number of deaths due to IBD was 13905.226, with an age-standardized mortality rate of 0.295 per 100,000 population in 2023(Table 2). Between 1990 and 2023, the age-standardized incidence rate of IBD in Asia increased at an average annual rate of 1.95, while the age-standardized DALY rate decreased on average by −0.19. The age-standardized prevalence rate of IBD increased at an average annual rate of 1.95, and the age-standardized mortality rate remained relatively stable, with an average annual change of −0.13.

**Table 1:** Incidence and DALYs of IBD, UC and CD in 1990 and 2023, and estimated annual percentage changes from 1990 to 2023.

	Incidence					DALYs				
	Number of cases, 1990	age-standardized rate per 100 000 population, 1990	Number of cases, 2023	age-standardized rate per 100 000 population, 2023	Estimated annual percentage change, 1990–2023	Count, 1990	age-standardized rate per 100 000 population, 1990	Count, 2023	age-standardized rate per 100 000 population, 2023	Estimated annual percentage change, 1990–2023
**IBD**										
Asia	61579.599 (52039.261-74404.925)	1.933(1.633-2.335)	160997.546 (135263.496-196176.776)	3.423(2.875-4.171)	1.95 (1.80,2.11)	392101.775 (247458.443-585933.879)	12.306(7.766-18.389)	568341.629 (407090.384-763445.860)	12.078 (8.654-16.222)	−0.19(−0.25,-0.13)
**Sex**										
Male	32302.998 (27178.137-39291.959)	1.979(1.665-2.407)	83041.857 (69311.152-101792.048)	3.466 (2.892-4.249)	1.89 (1.74,2.04)	204666.630 (113663.001-346096.503)	12.538 (6.963-21.203)	308054.058 (204602.601-455150.550)	12.855 (8.541-18.986)	−0.03 (−0.10,0.04)
Female	29276.601 (24752.404-35267.329)	1.884(1.593-2.270)	77955.688 (65652.731-95055.570)	3.378 (2.844-4.119)	2.01 (1.85,2.18)	187435.145 (100349.383-322203.703)	12.062(6.458-20.734)	260287.571 (172794.038-369388.813)	11.272 (7.485-15.995)	−0.37(−0.44,-0.31)
**Region**										
East Asia	12971.320 (11049.762-15638.368)	0.947 (0.806-1.141)	32635.335 (27604.809-39637.267)	1.994(1.686-2.421)	2.81 (2.31,3.31)	163140.531 (93611.097-246958.251)	11.904 (6.831-18.021)	153760.895 (118744.113-199275.008)	9.393 (7.254-12.174)	−0.90(−1.08,-0.72)
Southeast Asia	2147.358 (1825.941- 2609.802)	0.476 (0.405-0.578)	4760.958 (3995.096- 5832.054)	0.685(0.575-0.839)	1.13 (1.10,1.17)	33454.703 (18209.896-56709.915)	7.413 (4.035-12.566)	53697.456 (32908.962-82467.688)	7.728 (4.736-11.869)	0.02 (−0.10,0.13)
South Asia	39806.314 (33500.587-48083.014)	3.575 (3.009-4.318)	106795.914 (89656.518-130132.053)	5.470(4.592-6.665)	1.41 (1.35,1.48)	157510.504 (108644.420-228877.969)	14.146 (9.758-20.556)	298277.608 (208050.032-400933.822)	15.277 (10.656-20.535)	0.12 (0.05,0.19)
Central Asia	1690.480 (1432.666-2036.523)	3.291 (2.789-3.965)	3863.458 (3208.448-4688.884)	4.872 (4.046-5.913)	1.17 (1.09,1.25)	10295.206 (8398.116-12687.508)	20.045 (16.351-24.702)	13740.372 (11021.619-16803.609)	17.329 (13.900-21.192)	−0.24 (−0.46,-0.01)
West Asia	4964.126 (4230.305-6037.220)	2.485 (2.118-3.022)	12941.880 (10798.626-15886.518)	3.786 (3.158-4.649)	1.31 (1.21,1.42)	27700.830 (18594.914-40700.236)	13.868 (9.309-20.376)	48865.298 (36365.659-63965.733)	14.215 (10.597-18.583)	−0.01 (−0.10,0.08)
**Type**										
UC	44199.092 (35307.486-56884.575)	1.387 (1.108-1.785)	116396.510 (91568.297-152133.542)	2.474 (1.947-3.234)	2.00 (1.85,2.15)	326187.834 (196196.280-496921.223)	10.237(6.158-15.596)	446994.659 (314083.027-606710.281)	9.494 (6.672-12.887)	−0.38 (−0.46,-0.31)
CD	17380.507 (14233.114-22145.278)	0.545 (0.447-0.695)	44601.035 (36954.436-56036.544)	0.948 (0.785-1.191)	1.83 (1.66,2.00)	65913.941 (42766.310-102249.792)	2.069(1.342-3.209)	121346.970 (80043.288-173681.489)	2.584 (1.705-3.696)	0.63 (0.55,0.71)

**Table 2:** Prevalence and deaths of IBD, UC and CD in 1990 and 2023, and estimated annual percentage changes from 1990 to 2023.

	Prevalence					Deaths				
	Number of cases, 1990	age-standardized rate per 100 000 population, 1990	Number of cases, 2023	age-standardized rate per 100 000 population, 2023	Estimated annual percentage change, 1990–2023	Count, 1990	age-standardized rate per 100 000 population, 1990	Count, 2023	age-standardized rate per 100 000 population, 2023	Estimated annual percentage change, 1990–2023
**IBD**										
Asia	510410.972 (432139.454-602158.793)	16.019 (13.563-18.899)	1395394.162 (1187007.410-1653395.831)	29.691 (25.255-35.181)	1.95 (1.76,2.13)	9074.151 (5159.591-14498.402)	0.285 (0.162-0.455)	13905.226 (8942.550-20688.662)	0.295 (0.190-0.439)	-0.13 (-0.26,0.00)
**Sex**										
Male	259790.732 (220236.862-307676.211)	15.915 (13.492-18.849)	700189.852 (596432.485-831318.702)	29.242 (24.906-34.719)	1.90 (1.72,2.07)	4472.102 (2107.051-8255.284)	0.274 (0.129-0.506)	7522.884 (4259.898-12527.514)	0.314 (0.178-0.522)	0.26 (0.16,0.37)
Female	250620.241 (211640.508-299906.341)	16.128 (13.619-19.300)	695204.311 (590266.007-829874.454)	30.156 (25.603-35.999)	1.99 (1.80,2.19)	4602.050 (1967.829-8635.782)	0.296 (0.127-0.556)	6382.342 (3243.364-10961.521)	0.276 (0.140-0.474)	-0.56 (-0.73,-0.38)
**Region**										
East Asia	120797.879 (103327.275-142003.633)	8.815 (7.540-10.362)	278340.454 (239397.707-329021.297)	17.004 (14.625-20.100)	2.42 (1.87,2.98)	9074.151 (5159.591-14498.402)	0.325 (0.182-0.499)	13905.226 (8942.550-20688.662)	0.355 (0.254-0.490)	-0.21 (-0.50,0.08)
Southeast Asia	19251.477 (16181.968-22779.233)	4.266 (3.586-5.047)	43663.125 (37321.678-51461.160)	6.284 (5.371-7.406)	1.16 (1.12,1.21)	866.036 (459.115-1516.471)	0.192 (0.102-0.336)	1619.676 (927.428-2547.738)	0.233 (0.133-0.367)	0.45(0.33,0.57)
South Asia	302030.123 (254911.929-355984.431)	27.126 (22.894-31.972)	883817.930 (749806.356-1046727.034)	45.267 (38.404-53.611)	1.51 (1.42,1.60)	3047.824 (1752.394-5063.676)	0.274 (0.157-0.455)	5326.440(3083.700-8476.317)	0.273(0.158-0.434)	-0.08 (-0.20,0.03)
Central Asia	17709.136 (14980.402-21071.393)	34.479 (29.166-41.025)	36247.833 (30396.031-43689.129)	45.715 (38.334-55.099)	1.06 (0.99,1.13)	168.979 (133.660-216.007)	0.329 (0.260-0.421)	207.151 (166.046-259.964)	0.261 (0.209-0.328)	-0.55 (-0.90,-0.20)
West Asia	50622.358 (42737.880-60320.102)	25.344 (21.396-30.199)	153324.820 (130085.637-182497.211)	44.817 (38.003-53.338)	1.59 (1.43,1.75)	539.859 (313.511-861.354)	0.270 (0.157-0.431)	948.700 (602.963-1380.077)	0.275 (0.176-0.398)	0.02(-0.21,0.25)
**Type**										
UC	358996.059 (287357.875-448423.054)	11.267 (9.019-14.074)	986032.939 (788460.994-1233579.282)	20.981 (16.776-26.247)	1.99 (1.82,2.16)	8107.790 (4557.696-12954.822)	0.254 (0.143-0.407)	12350.852 (7917.522-18425.531)	0.262 (0.168-0.391)	-0.17 (-0.31,-0.03)
CD	151414.913 (118999.308-192651.626)	4.752 (3.735-6.046)	409361.224 (326114.805-512820.751)	8.710 (6.938-10.912)	1.84 (1.63,2.05)	966.362 (454.339-1799.297)	0.030 (0.014-0.056)	1554.374 (773.789-2795.411)	0.033 (0.017-0.060)	0.20 (0.11,0.29)

A total of 83,041.857 new cases of IBD were reported in men, which was higher than the 77,955.688 cases in women in 2023. The age-standardized incidence rate for IBD was 3.466 per 100,000 population in men, compared to 3.378 in women. Both sexes exhibited peak age-standardized incidence rates in the 45–49 age group (Fig 1A). The total number of DALYs due to IBD in men was 308,054.058, higher than the 260,287.571 in women. The age-standardized DALY rate for men was 12.855, while for women it was 11.272. DALY rates increased with age for both genders, as shown in Fig 1B. Regarding prevalence, the total number of prevalent cases in men was 700,189.852, higher than the 695,204.311 in women. However, the age-standardized prevalence rate was higher in women, at 30.156, compared to 29.242 in men. Both sexes exhibited peak prevalence rates in the 60–64 age group, as shown in Fig 1C. For mortality, the total number of deaths due to IBD in men was 7,522.884, which was higher than the 6,382.342 in women. The mortality rate followed a similar pattern, with men at 0.314 and women at 0.276. The age-related trends in mortality are depicted in Fig 1D. Sex-specific analyses showed that the magnitude and direction of sex differences varied across Asian subregions, disease subtypes, and burden measures (S21 and S24 Tables in S1 File). Male-to-female ratios were not uniform across regions or outcomes, indicating that sex-related differences in IBD burden were heterogeneous across Asia (S7 Fig in S1 File). Although males generally had higher ASDR and ASMR for IBD overall, this pattern was not uniform across all settings. Male rates were generally higher for IBD and UC in several regions, whereas the magnitude and direction of the disparity were less consistent for CD. In particular, female CD burden was comparable to or higher than male burden in some settings, such as South Asia. These findings suggest that sex disparities in IBD burden are context-dependent rather than uniform across all regions and subtypes.

**Fig 1:**
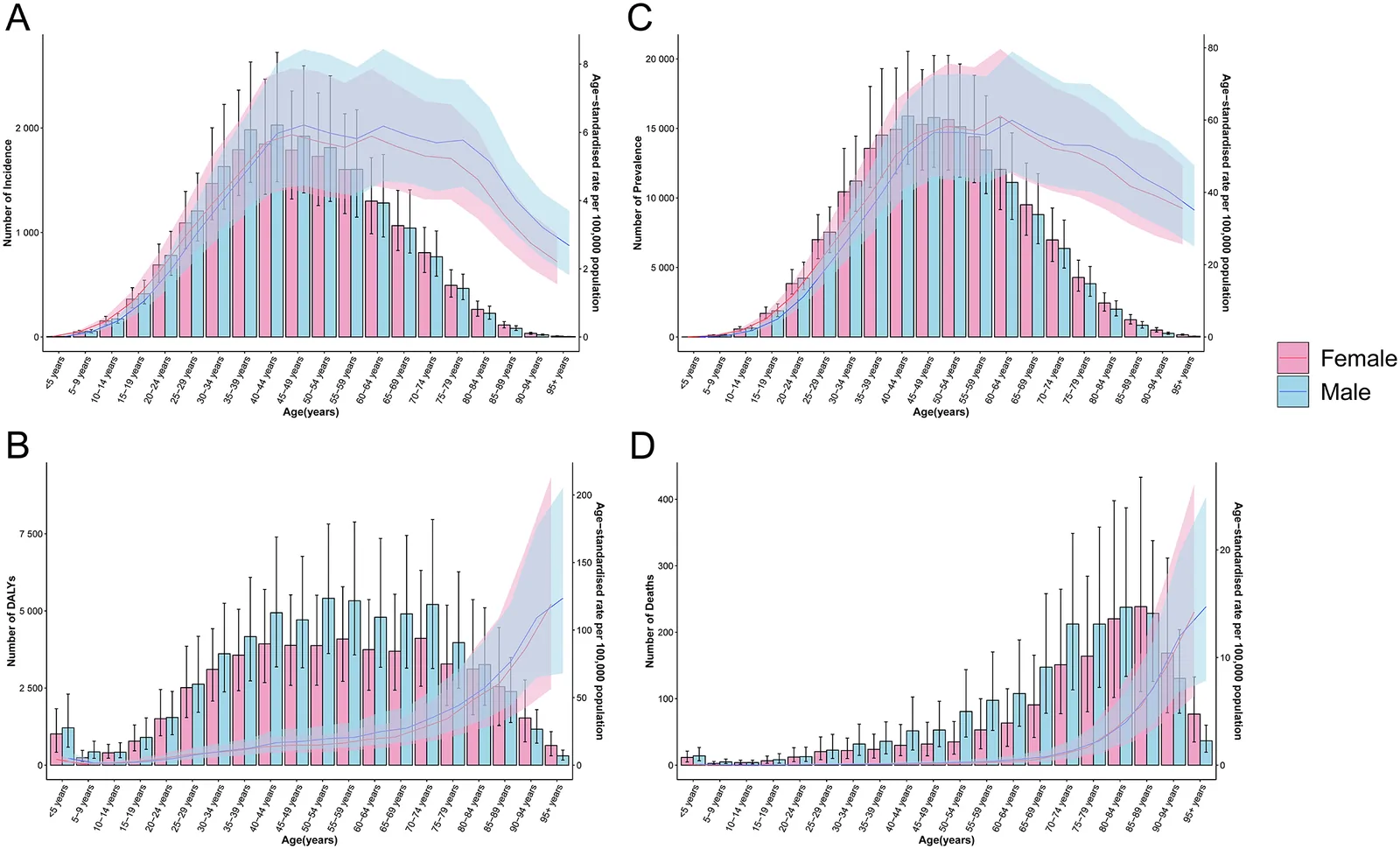
Age-standardized rates and total number of incidence (A), DALYs (B), prevalence (C),deaths (D) of IBD by sex in Asia for the year 2023.

In 2023, the number of new cases of UC was 116,396.510, which was higher than the 44,601.035 new cases of CD. The age-standardized incidence rates followed the same pattern, with UC at 2.474 and CD at 0.948. The incidence rate of UC peaked in the 45–49 age group, while the incidence rate of CD peaked in the 40–44 age group (Fig 2A). The total number of DALYs for UC in 2023 was 446,994.659, which was higher than the 121,346.970 for CD. The age-standardized DALY rate for UC was 9.494, higher than the 2.584 for CD. However, the age-standardized DALY rate for UC showed a decreasing trend, with an EAPC of −0.38, while the EAPC for CD’s DALY rate was 0.63. The rate of increase in DALYs with age was steeper for UC than for CD, as shown in Fig 2B. The total prevalence of UC in 2023 was 986,032.939, which was higher than the 409,361.224 for CD. The age-standardized prevalence rate for UC was 20.981, compared to 8.710 for CD. The age-specific prevalence rate for UC peaked at 60–64 years, whereas the aggregate Asian CD prevalence curve showed two apparent peaks at 45–49 and 60–64 years (Fig 2C). Detailed trends are shown in Fig 2C. The total number of deaths due to UC in 2023 was 12,350.852, which was higher than the 1,554.374 for CD. The age-standardized mortality rate for UC was 0.262, also higher than the 0.033 for CD. However, the mortality rate for UC showed a decreasing trend, with an EAPC of −0.17. Despite this, UC mortality rates were higher than those of CD as age increased, as detailed in Fig 2D. The age-standardized incidence, DALY, prevalence, and mortality rates for IBD, UC, and CD in 47 countries in Asia for the years 1990 and 2023, along with their corresponding EAPC, can be found in S1-S12 Tables (S1 File). Across Asia, UC and CD showed similar increases in age-standardized incidence and prevalence, with no significant differences in temporal slopes after FDR correction. In contrast, UC DALY and mortality rates decreased, whereas the corresponding CD rates increased (both FDR-adjusted P < 0.001). These subtype differences varied across subregions (S22 Table and S4 Fig in S1 File). UC-to-CD ratios also showed heterogeneous temporal patterns across burden measures and subregions. Across Asia, the UC-to-CD ratios increased slightly for incidence and prevalence but decreased from 4.949 to 3.684 for DALYs and from 8.390 to 7.946 for mortality between 1990 and 2023 (S23 Table,S6 Fig in S1 File). Subregional analyses showed that the apparent bimodal CD prevalence pattern was reproduced only in East Asia and was not observed consistently in Southeast Asia, South Asia, Central Asia, or West Asia (S5 Fig in S1 File).

**Fig 2:**
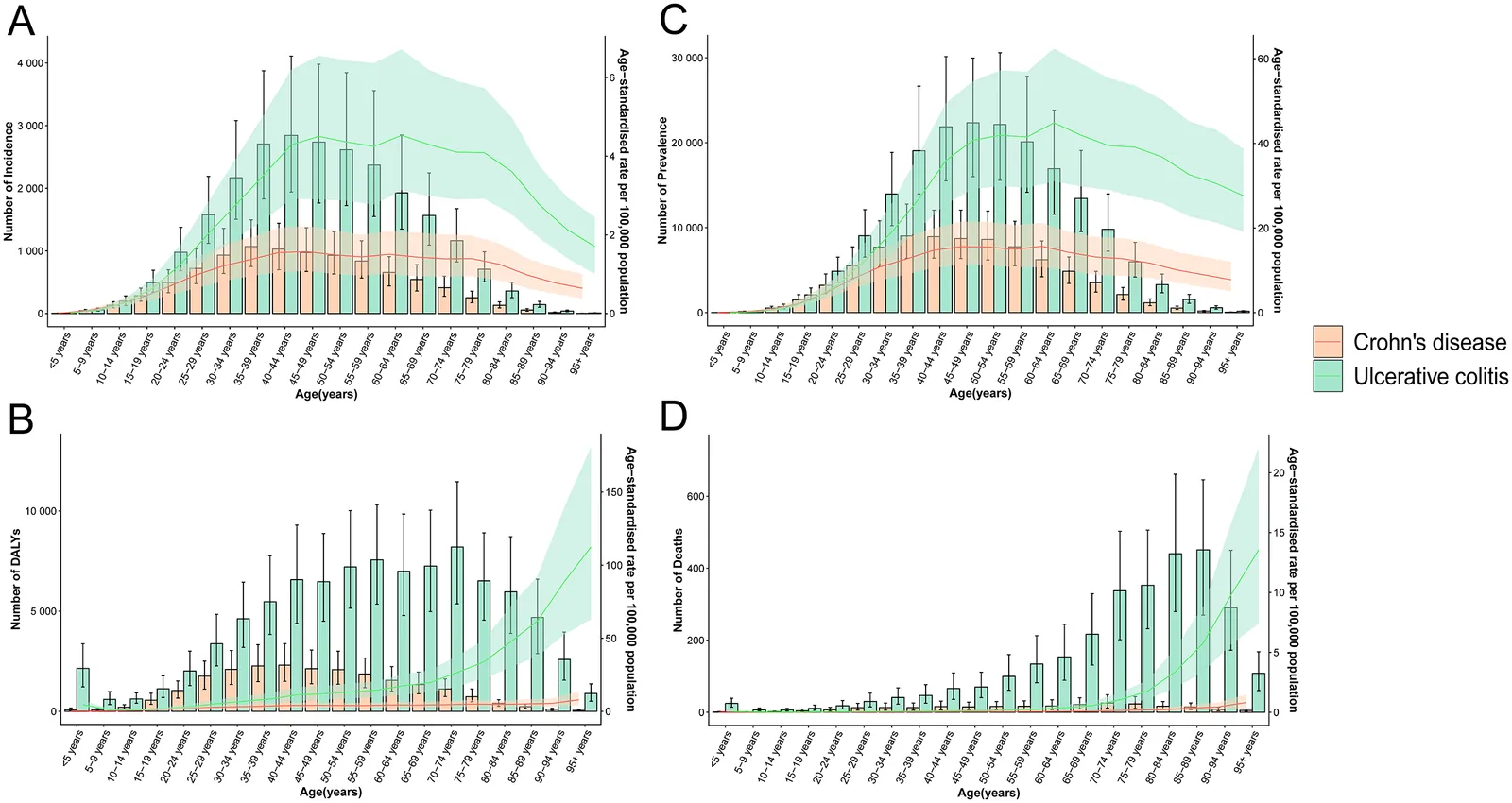
Age-standardized rates and total number of incidence (A), DALYs (B), prevalence (C),deaths (D) of IBD, categorized by UC and CD, in Asia for the year 2023. IBD = inflammatory bowel disease; UC = ulcerative colitis; CD = Crohn’s disease.

In 2023, South Asia had the highest age-standardized incidence rate of 5.470 in Asia(Table 1, Fig 3A). Between 1990 and 2023, East Asia experienced the largest increase in the age-standardized incidence rate, with an EAPC of 2.81 (95% CI: 2.31–3.31) (Fig 4A). During the same period, Central Asia had the highest age-standardized DALY rate, at 17.329 (Table 1, Fig 3B). Between 1990 and 2023, Southeast Asia experienced the largest increase in the age-standardized DALY rate, with an EAPC of 0.02, while East Asia saw the largest decrease, with an EAPC of −0.90 (Fig 4B). The highest age-standardized prevalence rates were observed in Central Asia at 45.715 and South Asia at 45.267 (Table 2, Fig 3C). Between 1990 and 2023, East Asia experienced the largest increase in prevalence, with an EAPC of 2.42 (Fig 4C). The highest age-standardized mortality rate was observed in East Asia, with a rate of 0.355 (Table 2, Fig 3D). Between 1990 and 2023, Southeast Asia experienced the largest increase in the age-standardized mortality rate, with an EAPC of 0.45 (Fig 4D). The age-standardized incidence rates, DALY rate, prevalence rate, and mortality rate for UC and CD across Asian countries are provided in S1-S2 Figs in S1 File.

**Fig 3:**
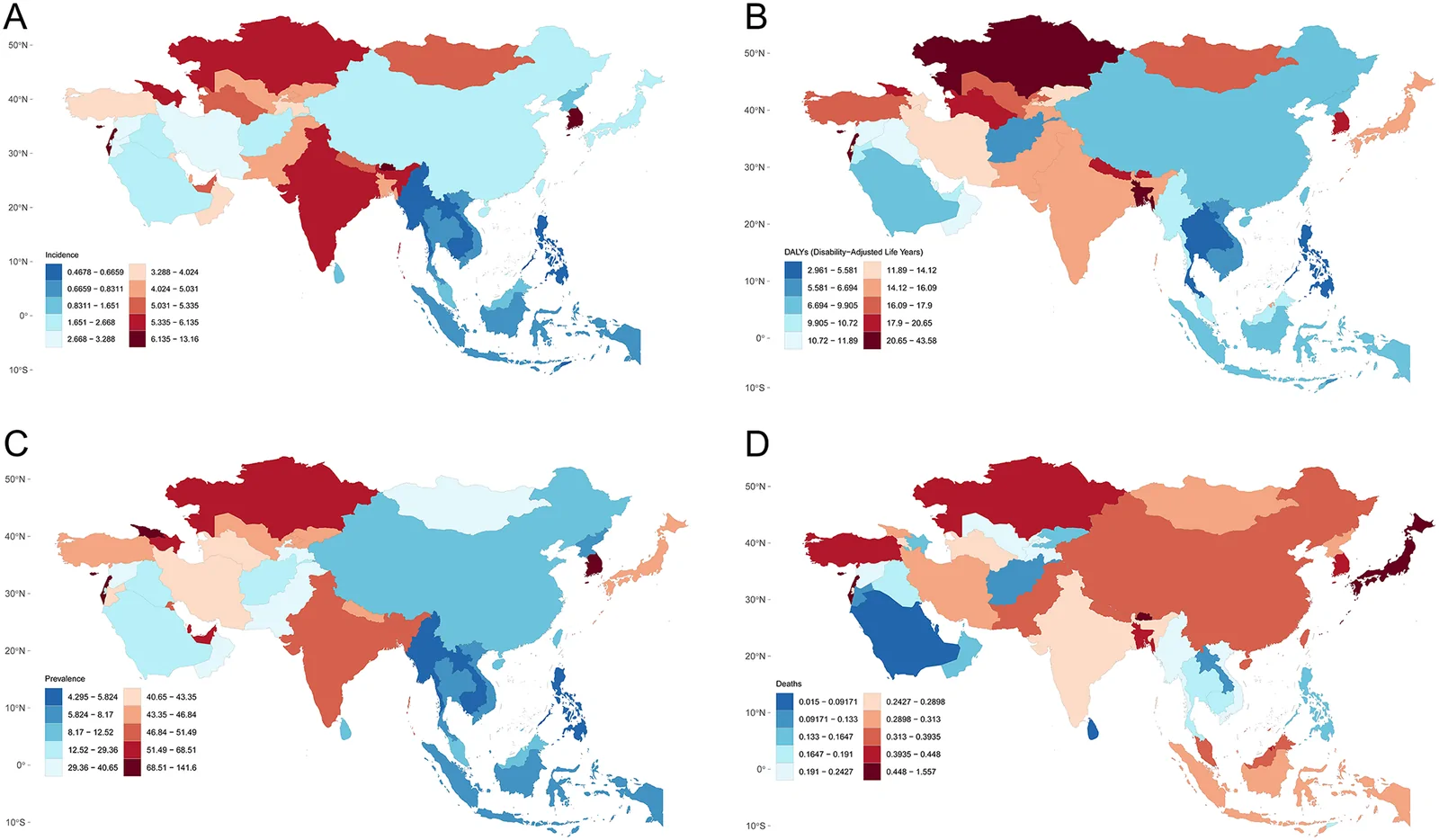
Age-standardized incidence rate (A), DALY rate (B), prevalence rate (C), and mortality rate (D) of IBD per 100,000 population in Asia, 2023. Base map data were obtained from Natural Earth (public domain). The figure was generated by the authors using original R code. The boundaries shown on this map do not imply any judgment regarding territorial sovereignty.

**Fig 4:**
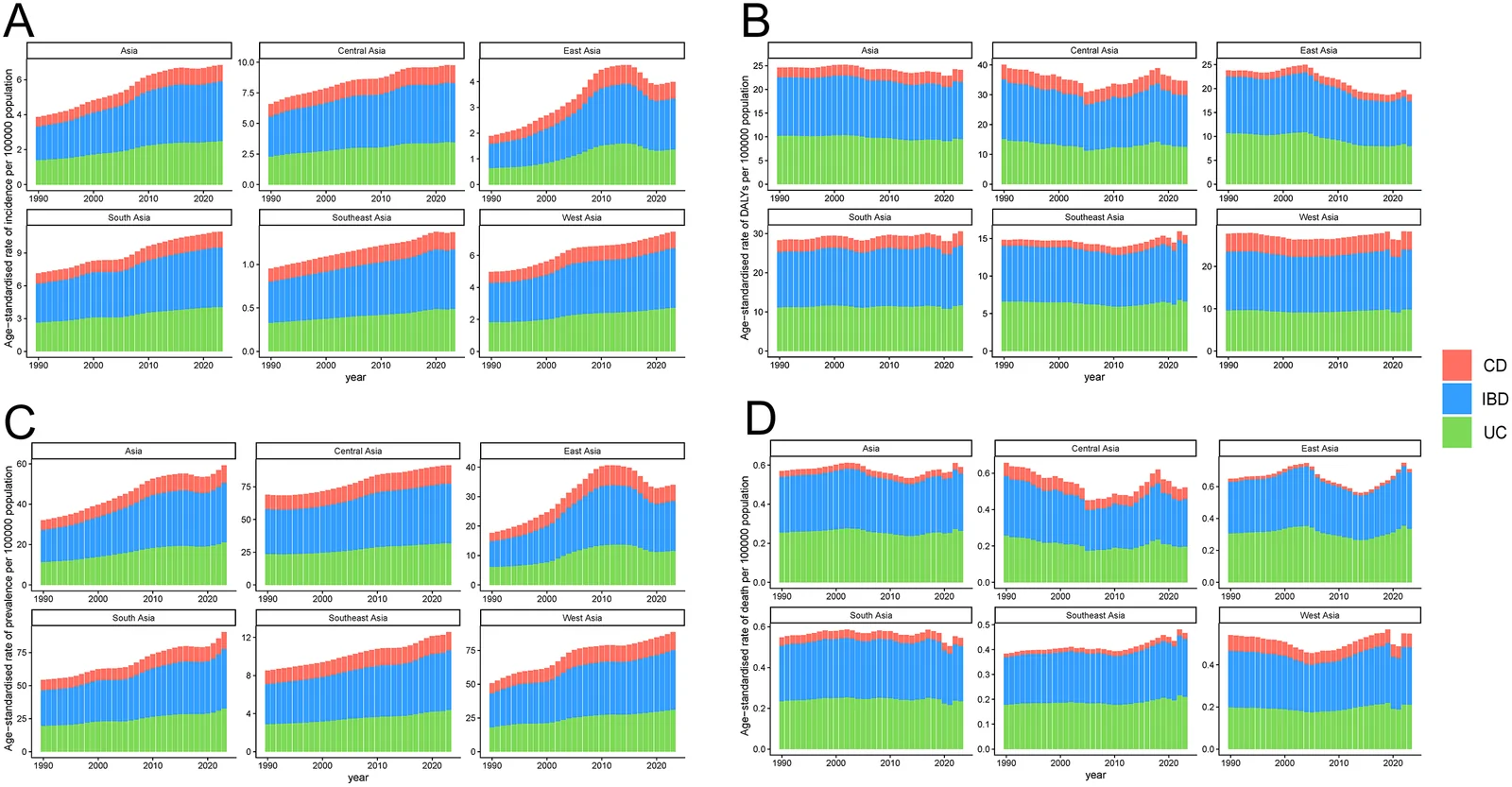
Trends in age-standardized incidence rate (A), DALY rate (B), prevalence rate (C), and mortality rate (D) for IBD, UC, and CD across Asia and its Sub-regions, 1990–2023.

Between 1990 and 2023, there was an overall increasing trend in age-standardized incidence and prevalence rates across Asia as SDI increased, with this upward trend observed in East Asia, Southeast Asia, South Asia, Central Asia, and West Asia (Fig 5A, 5B). In terms of age-standardized DALYs, different regions exhibited varying trends as SDI rose. Overall, Asia showed a negative correlation between SDI and DALYs, with East Asia and Central Asia demonstrating a negative relationship between age-standardized DALYs and SDI. Southeast Asia and West Asia, however, showed no significant relationship between SDI and DALYs, while South Asia showed a mild increasing trend (Fig 5C). Regarding mortality rates, no clear relationship was observed between age-standardized mortality rates and SDI across Asia. East Asia and West Asia showed no significant correlation, while Central Asia exhibited a negative correlation between SDI and mortality rates. Southeast Asia, on the other hand, showed a positive correlation (Fig 5D). The SDI values for Asia as a whole, as well as for East Asia, Southeast Asia, South Asia, Central Asia, and West Asia from 1990 to 2023, can be found in S13 Table (S1 File).

**Fig 5:**
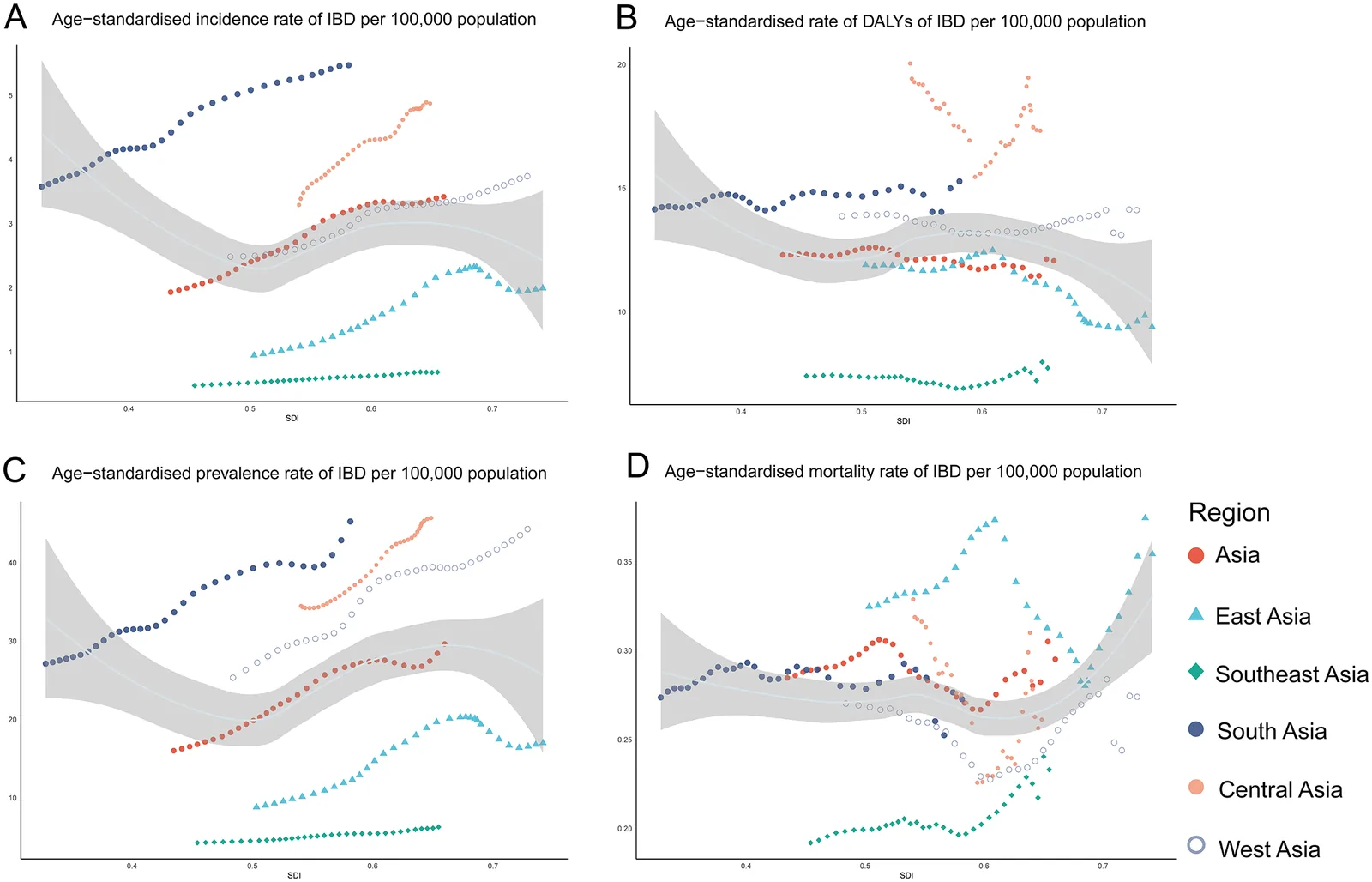
Age-standardized incidence rate (A), DALY rate (B), prevalence rate (C), and mortality rate (D) of IBD across Asia and its five sub-regions, stratified by SDI, 1990 - 2023.

At the country level in 2023, a 0.1-unit increase in SDI was associated with a 10.048-unit higher age-standardized prevalence rate (95% CI, 3.642–16.454; FDR-adjusted P = 0.011; R^2^ = 0.182; adjusted R^2^ = 0.163). Associations with incidence, DALYs, and mortality were not statistically significant after FDR correction. The corresponding regression coefficients, 95% CIs, P values, R^2^, adjusted R^2^, and AIC values are provided in S17 Table (S1 File). Natural cubic spline analyses provided no evidence of non-linearity for any burden measure (all FDR-adjusted P = 0.889; Fig 6; S17 Table in S1 File). By 2050, the age-standardized incidence, prevalence, and mortality rates of IBD were projected to reach 4.412 (95% PI, 3.838–4.986), 42.014 (37.054–46.975), and 0.295 (0.238–0.353) per 100,000 population, respectively. For UC, the corresponding projected rates were 3.205 (2.824–3.586), 30.043 (26.369–33.717), and 0.255 (0.199–0.311), respectively. For CD, the projected rates were 1.207 (1.003–1.410), 12.242 (10.224–14.261), and 0.032 (0.028–0.036), respectively (Fig 7; S14–S16 Tables in S1 File).

**Fig 6:**
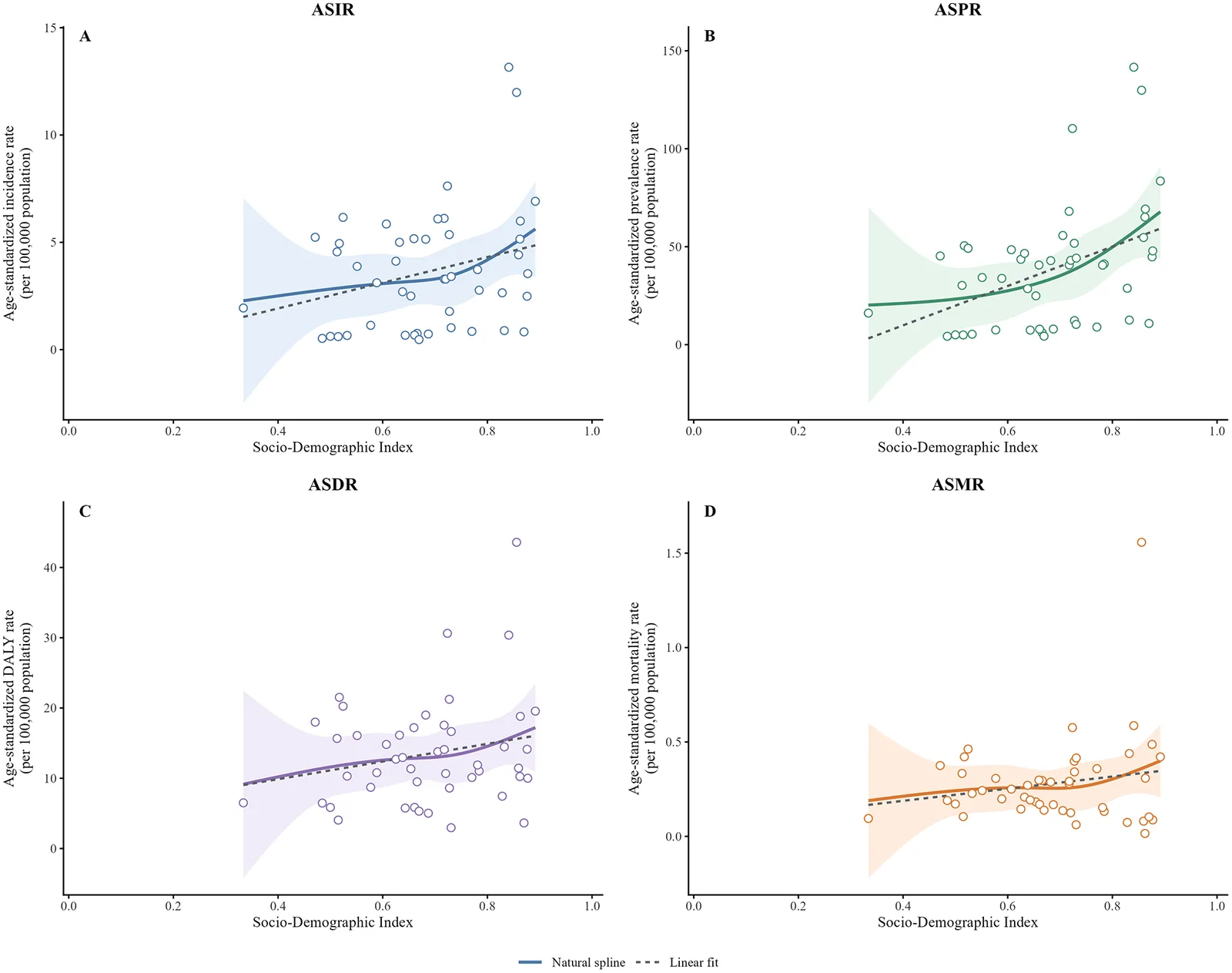
Country-level associations between SDI and age-standardized IBD burden measures in Asia, 2023. Panels show the associations of SDI with the age-standardized incidence rate (A), prevalence rate (B), DALY rate (C), and mortality rate (D). Solid curves and shaded areas represent natural cubic spline estimates and their 95% confidence intervals; dashed lines represent linear regression estimates.

**Fig 7:**
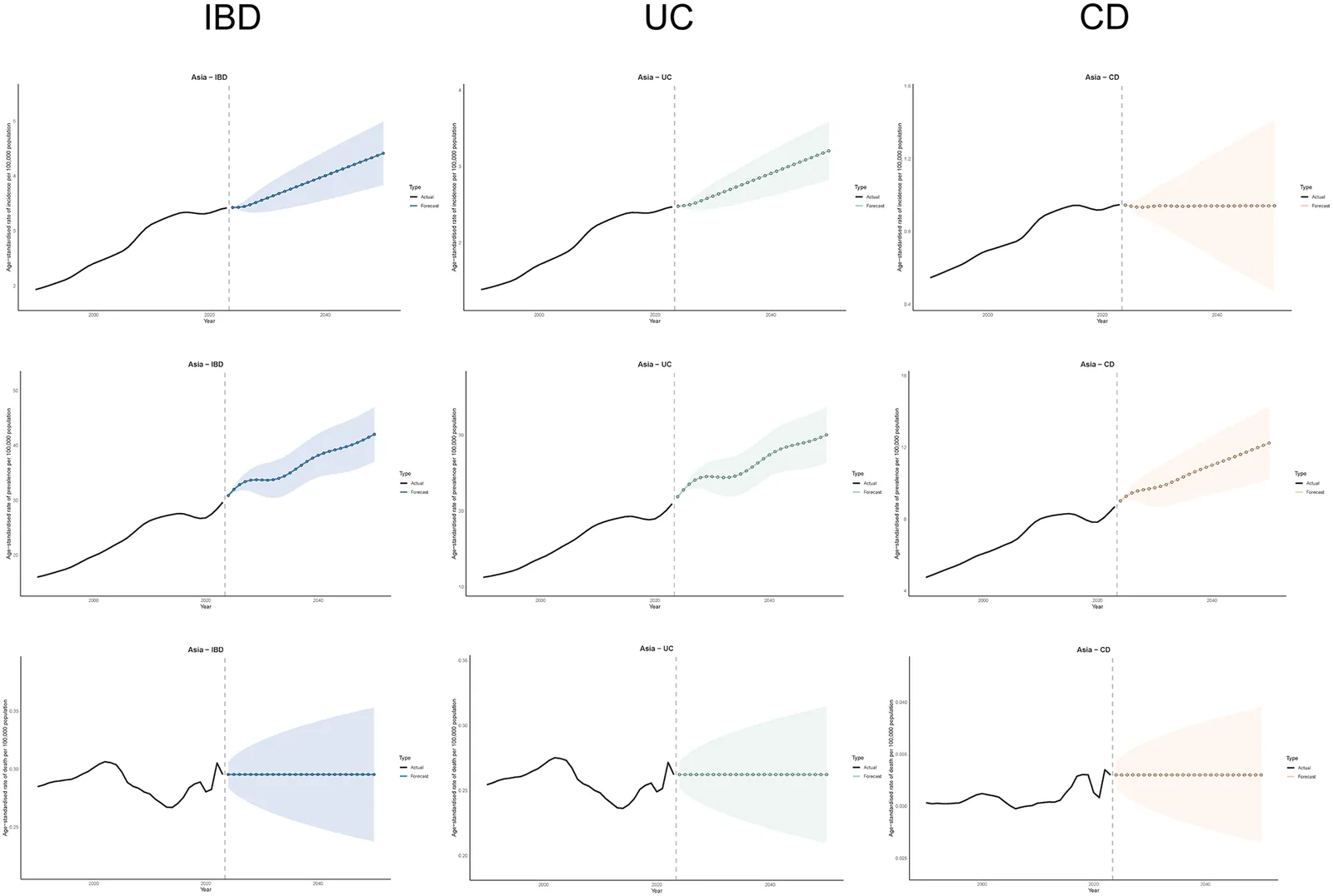
Projected age-standardized incidence, prevalence, and mortality rates for IBD, UC, and CD in Asia through 2050. Solid lines represent historical GBD point estimates, and forecast lines represent ARIMA point projections. Shaded areas represent 95% ARIMA prediction intervals conditional on the historical point-estimate series and do not incorporate uncertainty in the underlying GBD estimates.

Sensitivity analyses showed that the increasing incidence and prevalence trends remained directionally consistent when EAPCs were recalculated using the lower and upper bounds of the GBD 95% UIs (S18 Table in S1 File). In contrast, DALY and mortality trends were generally not directionally consistent, except in Central Asia, and should therefore be interpreted cautiously. Countries with the greatest relative uncertainty differed by outcome and included several data-limited settings, such as Myanmar, Cambodia, Bhutan, Lao PDR, Afghanistan, and the Democratic People’s Republic of Korea (S19 Table in S1 File). Excluding high-uncertainty countries did not materially change the absence of significant SDI associations for incidence, DALYs, or mortality. The positive SDI–prevalence association remained directionally consistent, although it was attenuated after exclusion of high-uncertainty countries (Pearson r = 0.379; FDR-adjusted P = 0.099; S17 Table in S1 File). Exclusion of Afghanistan, Myanmar, and Lao PDR produced similar conclusions.

Out-of-sample validation showed variable performance across the nine disease–measure series (S20 Table in S1 File). The selected ARIMA specifications, AICc values, and residual diagnostics are also reported in S20 Table in S1 File. MAE ranged from 0.0011 to 1.6718, RMSE from 0.0014 to 2.0822, and MAPE from 0.280% to 5.796%. Coverage of the 95% prediction intervals was 100% for incidence models, 0%–25% for prevalence models, and 50% for mortality models. All final-model Ljung–Box P values were greater than 0.05, indicating no evidence of residual autocorrelation. However, the limited prediction-interval coverage for some prevalence and mortality series indicates that these long-term projections should be interpreted cautiously.

## Discussion

In this study, we standardized the assessment of the burden of IBD in Asia, considering the entire region as well as its sub-regions (East Asia, Southeast Asia, South Asia, Central Asia, and West Asia), gender, SDI, and the trends from 1990 to 2023. We also projected the disease burden of IBD from 2024 to 2050. We report that, in 2023, Asia experienced 0.16 million new cases of IBD, 0.57 million DALYs, 1.39 million prevalent cases, and 13.91 thousand deaths. Between 1990 and 2023, both incidence and prevalence steadily increased, while the rise in DALYs slowed, as reflected by the negative EAPC for the age-standardized DALY rate. Interestingly, the mortality rate showed little to no change during the same period. The increasing age-standardized incidence and prevalence rates may reflect both genuine epidemiological changes and improved diagnosis and case ascertainment, whereas the declining age-standardized DALY rate may partly reflect improvements in disease management.However, the relatively stable mortality rate and the negative trend in age-standardized DALY rates may point to evolving treatment strategies or changes in the underlying health infrastructure across the region.Moreover, gender disparities are evident in the data, with men generally showing higher age-standardized incidence, DALY, and mortality rates, whereas prevalence differences were smaller and varied across regions and disease subtypes. Understanding the intersection of gender, socio-economic factors (as indicated by SDI), and disease burden across regions will be essential for targeted interventions and policy development aimed at reducing IBD’s impact on the Asian population.

These trends appear to be consistent with a broader but asynchronous global evolution of IBD. Recent global burden analyses and epidemiological reviews indicate that North America and Western Europe remain high-prevalence regions, whereas many newly industrialized regions continue to experience increasing incidence and prevalence. In this context, the upward trends observed in Asia may reflect an earlier stage of epidemiological transition rather than the later phase of compounding prevalence described in long-industrialized settings. Comparative evidence from Asian populations and Asian migrants to Western countries further suggests that the increase observed in Asia is not an isolated phenomenon, but part of a wider worldwide shift in IBD epidemiology, with substantial regional variation in timing and magnitude [3,6,7,21].

We found that in 2023, the highest age-standardized incidence rate of IBD was observed in South Asia, while East Asia saw the fastest increase in incidence from 1990 to 2023. Central Asia had the highest DALY rate, with Southeast Asia experiencing the largest increase in DALYs. The highest age-standardized prevalence rates were found in Central Asia and South Asia, while East Asia showed the greatest increase in prevalence. The highest mortality rate was also observed in East Asia, with the largest increase in Southeast Asia. These findings suggest substantial regional differences in the estimated burden of IBD. However, the magnitude of these differences should be interpreted cautiously because the availability and quality of primary IBD data vary considerably across Asian countries. In countries with limited population-based surveillance, disease registries, vital registration, or access to endoscopic diagnosis, GBD estimates may depend more heavily on statistical modelling, predictive covariates, and information borrowed across locations and periods. Consequently, some apparent differences between countries or subregions may reflect variation in data availability, diagnostic capacity, and model dependence in addition to genuine epidemiological variation. The increasing incidence and prevalence observed in Asia may reflect not only a true rise in disease occurrence, but also improvements in case ascertainment over time. Expanded access to diagnostic evaluation, greater clinician awareness, and strengthening of clinical and surveillance systems may have increased the likelihood of diagnosis and reporting, particularly in settings where historical underdiagnosis was likely. Healthcare utilisation and diagnostic delay are also important determinants of the observed IBD burden. Differences in healthcare-seeking behaviour, access to specialist services, and time from symptom onset to diagnosis may affect when IBD is recognised and recorded [22]. Improvements in endoscopy, imaging, histopathology, biomarkers, and disease activity assessment may have further increased diagnostic sensitivity and influenced epidemiological patterns over time [23–25].Therefore, the observed increase in age-standardized incidence should be interpreted cautiously. It may reflect a combination of genuine epidemiological change and improved case detection, healthcare utilization, diagnostic capacity, and reporting, rather than a true increase in disease occurrence alone.

Regional variation in IBD burden may also reflect differences in healthcare delivery across Asia. The expanding use of biologics, small molecules, and other advanced therapies has enabled earlier treatment escalation and improved disease control. However, prolonged treatment entails substantial drug and monitoring costs, potential adverse effects, and complex clinical decisions regarding treatment continuation or withdrawal [26]. Across Asian healthcare systems, differences in reimbursement, affordability, specialist capacity, and access to infusion and monitoring services may lead to unequal treatment uptake and clinical benefit, potentially contributing to regional heterogeneity in long-term outcomes [7,26].

The higher age-standardized incidence rates in South and Central Asia and the rapid increase observed in East Asia may reflect a combination of genuine epidemiological changes, including urbanization and changing environmental or lifestyle exposures, and improved diagnosis, clinical awareness, endoscopic access, and case reporting [27]. Because these rates were age-standardized, population growth and population ageing do not explain the temporal changes in incidence rates. Changes in the age-standardized incidence rate should instead be interpreted in relation to underlying epidemiological changes and improvements in case detection, diagnosis, and reporting. Central Asia’s higher DALY rate may indicate a greater severity of disease or delayed diagnoses, potentially reflecting gaps in healthcare access or diagnostic capabilities [28]. The rise in DALYs in Southeast Asia suggests increasing disease prevalence, possibly due to lifestyle changes in urbanized areas, including diet and environmental factors. With rapid urbanization, there has been a marked shift in dietary patterns, such as an increased consumption of processed foods, sugars, and fats, contributing to rising rates of chronic conditions like cardiovascular diseases, diabetes, and obesity [29]. Moreover, environmental factors like air pollution, water contamination, and reduced physical activity in urban settings may further exacerbate health outcomes [30]. Changes in cigarette smoking patterns may also have contributed to the observed trends in IBD burden. Smoking is an important environmental factor in IBD, particularly for Crohn’s disease, although its association with ulcerative colitis is more complex [31]. Because smoking prevalence has varied over time and across Asian populations, it may partly underlie some of the regional and temporal heterogeneity observed here. However, our study was not designed to directly assess the contribution of smoking to these trends.The increase in mortality rates in East Asia could be linked to the rising burden of disease in older age groups, who are more vulnerable to severe outcomes, or to the progression of long-standing IBD [32].

In Asia, men have higher age-standardized DALY and mortality rates for IBD compared to women, but lower incidence and prevalence rates. Both sexes exhibited peak age-specific incidence rates in the 45–49 age group, and both sexes also showed peak prevalence rates in the 60–64 age group.This phenomenon can be explained by several factors. The higher DALY and mortality rates in men may reflect more severe disease outcomes once diagnosed, possibly due to delayed healthcare seeking, less proactive management, or the higher severity of disease progression in men [33]. Additionally, men may be more likely to experience complications related to IBD, which could lead to a higher burden of disease in terms of both mortality and disability.In contrast, the lower incidence and prevalence rates in men could suggest that IBD is diagnosed later in men compared to women, who may seek healthcare more frequently or be diagnosed at earlier stages of the disease. Social and cultural factors may play a role here, as women may be more likely to visit healthcare providers and report symptoms early, leading to higher diagnosis rates [34].The peaks in age-standardized incidence and prevalence rates in the 45–49 and 60–64 age groups are consistent with the known age-related patterns of IBD. The 45–49 age group may represent a time when IBD is diagnosed after years of symptom progression, while the 60–64 age group reflects the cumulative burden of disease in older adults. The aging population in Asia is likely contributing to the higher prevalence in this age group, as people with chronic conditions like IBD are living longer due to improvements in healthcare [32]. The apparent bimodal CD prevalence pattern was not consistent across Asian subregions and was reproduced only in East Asia. Therefore, the aggregate Asian pattern may reflect differences in regional age structure, case ascertainment, survival, cohort composition, or disease management and should not be interpreted as evidence of universally distinct CD phenotypes.

The sex-specific analyses showed that the magnitude and direction of sex differences varied across Asian subregions, disease subtypes, and burden measures, rather than following a uniform pattern across Asia. In general, males tended to have higher DALY and mortality rates for IBD and UC, but this pattern was less consistent for CD and was not uniform across all regions. These disparities may reflect a combination of biological differences, healthcare-seeking behavior, variation in disease course, delayed diagnosis, and unequal access to diagnosis and treatment. In some settings, sociocultural factors may also influence healthcare access for women, potentially contributing to differences in disease recognition, timing of diagnosis, and long-term management [34]. Therefore, the observed sex differences in IBD burden should be interpreted as context-dependent and shaped by multiple interacting clinical and social factors.

Despite the annual increase in incidence and prevalence rates of UC and CD, the DALY and mortality rates for UC have shown a consistent decline, whereas CD has experienced a continuous increase. The incidence rate of UC peaked in the 45–49 age group, while the incidence rate of CD peaked in the 40–44 age group. The age-standardized prevalence rate for UC reached its peak in the 60–64 age group, while CD exhibited a bimodal peak in both the 60–64 and 45–49 age groups.This phenomenon can be attributed to several factors. For UC, the decline in DALY and mortality rates could be related to the increasing availability and effectiveness of treatment options, such as biologic therapies, which have significantly improved disease management. As UC tends to have a more stable disease course compared to CD, earlier diagnosis and better treatment strategies likely contribute to improved long-term outcomes and reduced disease burden [35]. The reduction in mortality rates for UC may also be associated with enhanced medical infrastructure and increased awareness, which facilitate timely interventions and better management of complications [4].On the other hand, CD is characterized by its more complex and often progressive course, with many patients experiencing severe complications, such as bowel obstructions, fistulas, and abscesses [36]. As a result, CD continues to show an upward trend in both incidence and mortality rates. The higher incidence observed in the 40–44 age group may reflect cohort effects or age-related differences in disease recognition, although these mechanisms could not be directly evaluated in the present study. Additionally, the aging population and the increasing burden of chronic diseases may contribute to higher prevalence and DALY rates in older age groups.The increasing incidence and prevalence of CD, coupled with the rising DALY and mortality rates, highlight the growing burden of this disease. Factors such as delayed diagnosis, the chronic and complex nature of the disease, and the lack of widespread access to advanced treatments in some regions may explain the persistence of high disease burden for CD [21]. Several Asia-specific factors should also be considered. In settings where intestinal tuberculosis remains endemic, its clinical, endoscopic, and histological overlap with CD may contribute to delayed or incorrect diagnosis and affect the estimated CD burden [37]. Access to biologics, advanced therapies, specialist care, and therapeutic monitoring remains unequal across Asian countries because of differences in affordability, reimbursement, and healthcare infrastructure, potentially contributing to regional disparities in disease outcomes. The COVID-19 pandemic may have further disrupted endoscopy, outpatient follow-up, infusion services, and treatment continuity, potentially delaying IBD diagnosis and care in some settings [38]. However, the effects of these factors could not be quantified using the present GBD data.

Formal comparisons showed that the principal temporal differences between UC and CD in Asia involved DALY and mortality rates rather than incidence or prevalence. While UC DALY and mortality rates declined, the corresponding CD rates increased, although these subtype differences varied across subregions. The divergent trends observed for UC and CD merit more detailed interpretation. The decline in UC DALY and mortality rates, despite rising incidence and prevalence, may reflect earlier diagnosis, improved disease monitoring, and more effective long-term management in some settings [35]. A population-based study from Norway and Sweden reported substantial reductions in major surgery after the introduction of biologic therapy, with a greater decline in UC than in CD [45]. These findings suggest that treatment availability may influence long-term IBD outcomes, although this benefit may vary across Asian healthcare systems because access to advanced therapies remains unequal [39]. By contrast, the increasing DALY burden of CD may be related to its typically progressive disease course, higher cumulative risk of structural complications, and persistent delays in diagnosis or treatment initiation [36]. These differences may also partly reflect changing diagnostic patterns, particularly in regions where increasing endoscopic availability and greater clinical awareness have improved recognition of CD over time. The UC-to-CD ratios changed heterogeneously across burden measures and Asian subregions, suggesting that the relative contributions of UC and CD have evolved differently across the region. These differences may reflect variation in disease epidemiology, diagnostic practices, healthcare access, treatment availability, and survival.

SDI continues to play a significant role in influencing the burden of IBD [40]. Between 1990 and 2023, as SDI increased, the incidence and prevalence rates of IBD across Asia’s sub-regions consistently rose. However, the overall trend for DALY rates was downward, with the exception of South Asia, where DALYs increased. In terms of mortality rates, there was no significant change overall, except for Southeast Asia, which exhibited an upward trend. At the country level, higher SDI was significantly associated only with age-standardized prevalence after multiple-testing correction. This association may reflect better diagnostic capacity, case ascertainment, access to care, and longer survival in higher-SDI countries. Although the model fit was modest, these ecological and cross-sectional associations should not be interpreted as causal effects of SDI on IBD burden. The observed relationships may also reflect differences in diagnostic capacity, healthcare access, treatment availability, survival, and other unmeasured country-level factors. As SDI rises, countries tend to have better healthcare infrastructure, which likely leads to improved diagnosis and reporting of IBD cases, contributing to the higher incidence and prevalence rates observed in higher SDI countries [1,2]. The higher SDI may also reflect better access to healthcare, earlier detection, and more effective treatments, which can contribute to higher prevalence as individuals with IBD live longer with the condition [14,41].On the other hand, the decline in DALY rates in most regions, except South Asia, could be attributed to advancements in medical management and treatment of IBD, leading to reduced disease severity and better control of symptoms. In South Asia, where healthcare access and awareness may still be limited, increasing DALYs may reflect worsening outcomes or delayed diagnoses.The stability of mortality rates across most regions suggests that while the incidence and prevalence of IBD have increased, the survival rates of those diagnosed with the disease have improved, potentially due to advancements in medical care. Southeast Asia’s rising mortality rate could be related to a combination of increasing disease burden, delayed diagnoses, and limited access to advanced treatments in some parts of the region [42]. IBD has substantial heterogeneity in pathogenesis and treatment response, which may not be adequately captured by conventional clinical classification. Precision medicine based on genetic, immune, microbial, and other molecular features may improve treatment selection, but its implementation requires specialized expertise, diagnostic infrastructure, and financial resources that remain unevenly distributed across Asian healthcare systems [41]. This variation may contribute to differences in treatment response and long-term IBD outcomes between countries and subregions.

The projections for IBD in Asia from 2024 to 2050 indicate that both incidence and prevalence rates will continue to rise, while the mortality rate is expected to remain unchanged. For UC, the incidence and prevalence rates are predicted to keep increasing, with mortality rates staying stable. In contrast, for CD, only the prevalence rate is expected to increase, with no change in either incidence or mortality rates.These trends suggest a combination of factors. The projected increases in incidence and prevalence may reflect continuation of both underlying epidemiological changes and improvements in diagnostic capacity and case ascertainment; however, the ARIMA models cannot distinguish between these mechanisms. As healthcare access improves, more individuals are diagnosed, leading to higher prevalence, especially in higher SDI regions. The stable mortality rates in IBD and UC, despite rising incidence, reflect significant advancements in treatment and disease management. Biologics, immunomodulators, and other advanced therapies have improved IBD management, but their availability and affordability vary substantially across Asia; therefore, these benefits may not be equally realized across countries and populations [43]. Additionally, early diagnosis and better patient monitoring may enable timely intervention and help prevent severe disease progression [22,23]. As a result, while the number of diagnosed cases continues to rise due to better awareness and diagnostic tools, mortality rates remain stable or decline, highlighting the effectiveness of modern treatments and healthcare improvements.For CD, the rise in prevalence but stable incidence and mortality rates could indicate that while the disease is being diagnosed more frequently, the treatment and management of CD may be improving, preventing the disease from becoming fatal. However, the lack of significant change in mortality and incidence rates could also reflect ongoing challenges in effectively managing the disease, as CD often has more complex complications than UC. Additionally, the stability in mortality suggests that the disease burden, while increasing in terms of diagnosed cases, is not yet leading to significant changes in fatal outcomes, possibly due to improvements in the quality of life and long-term management [44].

Despite the comprehensive data provided by the GBD 2023 study, several limitations should be acknowledged. First, the availability and quality of primary epidemiological data on IBD vary substantially across Asian countries. In countries with limited population-based surveillance, disease registries, vital registration, or access to endoscopic and specialist diagnosis, GBD estimates rely more heavily on statistical modelling, predictive covariates, and information borrowed across locations, age groups, sexes, and time periods. Estimates from these data-sparse settings may therefore be more sensitive to model assumptions and may not fully capture local epidemiological patterns. Accordingly, estimates for countries such as Afghanistan, Myanmar, Lao PDR, Cambodia, Bhutan, and the Democratic People’s Republic of Korea should not be interpreted as having the same empirical reliability as estimates from countries with more complete surveillance. Their point estimates and country rankings are suitable for broad comparison but not for detecting small differences or informing precise national planning without corroborating local data.Incomplete case ascertainment and diagnostic delay may lead to underestimation of incidence and prevalence, whereas increased healthcare utilisation, improved access to specialist care, advances in diagnostic technology, and changes in reporting over time may produce apparent increases that do not represent true epidemiological change alone. Diagnostic misclassification, including difficulties in distinguishing Crohn’s disease from intestinal tuberculosis in endemic settings, may introduce additional uncertainty into the subtype-specific estimates. These limitations also affect the regional estimates because the subregional results were derived by aggregating country-level estimates. Uncertainty originating from data-sparse countries is therefore carried into the corresponding subregional estimates, while highly populated countries may disproportionately influence regional point estimates. Aggregation does not eliminate systematic errors related to underdiagnosis, misclassification, or shared GBD modelling assumptions. Therefore, small differences between countries or subregions, as well as country rankings based on point estimates, should be interpreted cautiously and in conjunction with the corresponding 95% uncertainty intervals. Moreover, the reported GBD 95% uncertainty intervals reflect uncertainty propagated through the GBD estimation framework but may not encompass all sources of uncertainty, including structural model misspecification, incomplete case detection, changes in diagnostic practice, and disease misclassification. The estimates should therefore be regarded as model-based comparative estimates rather than equivalent to directly observed surveillance counts. Second, the study only included countries in Asia, and although the regional divisions (East Asia, Southeast Asia, South Asia, Central Asia, and West Asia) are useful, they may not fully account for the specific cultural, health system, and environmental differences within sub-regions. For example, South Asia and Southeast Asia share some common characteristics, but health policies and disease burden profiles might differ significantly within these regions. Thus, regional variations may be oversimplified. Finally, the study focused primarily on the burden of IBD, UC, and CD, but other factors influencing disease progression and outcomes, such as environmental exposures (e.g., pollution, climate change), healthcare access, and individual lifestyle factors, were not fully incorporated. The GBD data did not allow us to quantify diagnostic misclassification between CD and intestinal tuberculosis, country-level access to biologics and advanced therapies, or disruptions in IBD diagnosis and care during the COVID-19 pandemic. These unmeasured factors may have influenced comparisons between countries and subregions.

The sensitivity analyses provided additional context for interpreting the GBD-derived estimates. The increasing directions of the age-standardized incidence and prevalence trends remained generally consistent when the analyses were repeated using the lower and upper bounds of the reported GBD 95% uncertainty intervals. This consistency provides some support for the principal conclusion that the estimated incidence and prevalence of IBD have increased in Asia. However, it does not eliminate uncertainty in the magnitude of these increases or confirm that they reflect true epidemiological change rather than changes in case detection and reporting. In contrast, the findings for age-standardized DALY and mortality rates were less stable across the alternative analyses, particularly in settings with limited primary epidemiological data, and should therefore be interpreted with greater caution. The positive association between SDI and prevalence was directionally consistent across alternative analyses, although it was attenuated after excluding high-uncertainty countries. Associations with incidence, DALYs, and mortality remained weaker and non-significant after FDR correction.

The ARIMA projections should be interpreted as conditional extrapolations of the historical GBD point-estimate series. Their 95% prediction intervals quantify ARIMA forecast uncertainty only and do not incorporate uncertainty in the underlying GBD estimates. Consequently, the total uncertainty surrounding the 2050 projections is likely greater than that represented by the reported intervals. The reported prediction intervals quantify uncertainty arising from the ARIMA models but do not incorporate uncertainty in the underlying GBD estimates. Consequently, the total uncertainty surrounding the 2050 projections is likely greater than that represented by the reported intervals. Although point-forecast errors were generally modest, prediction-interval coverage was limited for several prevalence and mortality series, and the four-year validation period provided only a limited assessment of long-term performance. Moreover, the models do not explicitly incorporate future changes in diagnostic capacity, treatment access, healthcare policy, or environmental exposures.

In light of the limitations and findings from this study, future research should focus on enhancing data quality and expanding the scope of factors influencing the burden of IBD, UC, and CD across Asia. While the GBD 2023 study provides comprehensive estimates, there is a need for more granular, high-quality data from countries with weaker health surveillance systems. This could involve improving national disease registries and using new technologies like mobile health platforms to ensure more accurate and real-time reporting, especially in low-resource settings. Future studies should incorporate a broader range of determinants beyond healthcare access and diagnostic capacity, including diet, nutrition, environmental exposures, and social conditions, to better explain the regional heterogeneity and temporal trends of IBD burden across Asia [45]. Factors such as rapid urbanization, dietary westernization, changes in food processing and consumption patterns, environmental pollution, climate change, and inequalities in education and healthcare access may all influence disease onset, progression, and long-term outcomes. Further investigation of the interactions between genetic susceptibility and environmental influences may provide additional insight into the rising burden of IBD in the region [21]. Moreover, longitudinal cohort studies are needed to monitor disease progression and assess the effectiveness of public health strategies over time. Such studies could generate real-world evidence on how early recognition, equitable access to care, chronic disease management, and treatment options affect long-term outcomes, thereby helping policymakers design more effective strategies to mitigate the future burden of IBD in Asia.

## Conclusion

This study highlights the growing burden of inflammatory bowel disease (IBD) in Asia, revealing significant increases in both incidence and prevalence between 1990 and 2023. In 2023, Asia recorded 160,997 new cases, 568,342 DALYs, and 1.39 million prevalent cases, with 13,905 deaths due to IBD. Regional variations were notable, with South Asia having the highest age-standardized incidence rate, Central Asia the highest DALY rate, and East Asia the largest increase in disease burden over the period. Gender disparities were also observed, with men showing higher DALY and mortality rates, while women had higher incidence and prevalence rates.

UC and CD displayed different trends, with UC’s DALY and mortality rates decreasing, while CD saw increases in both metrics. These differences suggest distinct disease trajectories and the need for tailored treatment strategies. The relatively stable mortality rate reflects improvements in disease management, but the rising incidence and prevalence highlight the need for ongoing public health efforts.

Projections for 2050 indicate continued increases in incidence and prevalence, with stable mortality rates. This underscores the importance of improving healthcare access and treatment strategies, particularly for complex diseases like CD. Regional differences point to the need for targeted interventions that consider local healthcare systems, lifestyles, and environmental factors. Continued research is crucial to better understand the factors driving the rise in IBD and to refine management strategies across Asia. Although the projected trends suggest a continued increase in the burden of IBD in Asia, these forecasts should be interpreted cautiously as trend-based projections derived from historical data rather than precise predictions of future epidemiologic change.

## Supporting information

S1 File Supplementary appendix containing Figures S1-S7 and Tables S1–S24. (DOCX)

S2 File IBD data and R code used to reproduce the analyses. (ZIP)
