# Supplementary material for: The burden of inflammatory bowel disease in Asia (1990–2023) and projections to 2050: A comprehensive analysis of the 2023 global burden of disease study
Source: PLoS One. 2026 Oct 8;21(10):e0359856. doi: 10.1371/journal.pone.0359856 (PMC13649031; doi:10.1371/journal.pone.0359856)
Supplement: Supplementary file 1 [file pone.0359856.s001.docx]

**Supplementary appendix**

**The Burden of Inflammatory Bowel Disease in Asia (1990-2023) and Projections to 2050: A Comprehensive Analysis of the 2023 Global Burden of Disease Study.**

**Chen et al.**

**Content**

[S1 Fig: Age-standardized incidence rate (A), DALY rate (B), prevalence rate (C), and mortality rate (D) of UC per 100,000 population in Asia, 2023. Base map data were obtained from Natural Earth (public domain). The figure was generated by the authors using original R code.	1](#_Toc23412)

[S2 Fig: Age-standardized incidence rate (E), DALY rate (F), prevalence rate (G), and mortality rate (H) of CD per 100,000 population in Asia, 2023. Base map data were obtained from Natural Earth (public domain). The figure was generated by the authors using original R code.	1](#_Toc11992)

[S3 Fig. Observed versus predicted IBD ASIR, ASPR, ASDR, and ASMR in Asia, 2020-2023.	2](#_Toc21860)

[S4 Fig. Temporal trends in age-standardized burden measures for ulcerative colitis and Crohn’s disease across Asia and its subregions, 1990–2023.	2](#_Toc20428)

[S5 Fig. Age-specific prevalence rates of Crohn’s disease across Asian subregions in 2023.	3](#_Toc26078)

[S6 Fig. Temporal trends in UC-to-CD ratios for age-standardized burden measures across Asia and its subregions, 1990–2023.The UC-to-CD ratio was calculated as the age-standardized UC rate divided by the corresponding age-standardized CD rate. Shaded areas represent approximate uncertainty ranges based on the country-level lower and upper estimates.	3](#_Toc25981)

[S7 Fig. Male-to-female ratios of age-standardized incidence, prevalence, DALY, and mortality rates for IBD, UC, and CD across Asia and its five subregions in 2023. A ratio greater than 1 indicates a higher rate in males, whereas a ratio below 1 indicates a higher rate in females.	4](#_Toc27661)

[S1 Table: Incidence of IBD in 1990 and 2023, and estimated annual percentage changes from 1990 to 2023.	5](#_Toc10052)

[S2 Table: DALYs of IBD in 1990 and 2023, and estimated annual percentage changes from 1990 to 2023.	8](#_Toc11100)

[S3 Table: Prevalence of IBD in 1990 and 2023, and estimated annual percentage changes from 1990 to 2023.	11](#_Toc22154)

[S4 Table: Deaths of IBD in 1990 and 2023, and estimated annual percentage changes from 1990 to 2023.	14](#_Toc25745)

[S5 Table: Incidence of UC in 1990 and 2023, and estimated annual percentage changes from 1990 to 2023.	17](#_Toc10743)

[S6 Table: DALYs of UC in 1990 and 2023, and estimated annual percentage changes from 1990 to 2023.	20](#_Toc28870)

[S7 Table: Prevalence of UC in 1990 and 2023, and estimated annual percentage changes from 1990 to 2023.	23](#_Toc26375)

[S8 Table: Deaths of UC in 1990 and 2023, and estimated annual percentage changes from 1990 to 2023.	26](#_Toc13606)

[S9 Table: Incidence of CD in 1990 and 2023, and estimated annual percentage changes from 1990 to 2023.	29](#_Toc17748)

[S10 Table: DALYs of CD in 1990 and 2023, and estimated annual percentage changes from 1990 to 2023.	32](#_Toc15768)

[S11 Table: Prevalence of CD in 1990 and 2023, and estimated annual percentage changes from 1990 to 2023.	35](#_Toc20755)

[S12 Table: Deaths of CD in 1990 and 2023, and estimated annual percentage changes from 1990 to 2023.	38](#_Toc32190)

[S13 Table: SDI for Asia, East Asia, Southeast Asia, South Asia, Central Asia, and West Asia from 1990 to 2023.	41](#_Toc28311)

[S14 Table: Projected age-standardized incidence, prevalence, and mortality rates for IBD in Asia through 2050.	42](#_Toc29427)

[S15 Table: Projected age-standardized incidence, prevalence, and mortality rates for UC in Asia through 2050.	43](#_Toc28377)

[S16 Table: Projected age-standardized incidence, prevalence, and mortality rates for CD in Asia through 2050.	44](#_Toc30507)

[S17 Table. Country-level associations between the Socio-Demographic Index and age-standardized IBD burden measures in Asia, 2023.	45](#_Toc25941)

[S18 Table. Sensitivity analyses of temporal trends in IBD burden from 1990 to 2023.	47](#_Toc21476)

[S19 Table. Country-level relative uncertainty in age-standardized IBD burden estimates in Asia, 2023.	48](#_Toc12735)

[S20 Table：ARIMA model selection, residual diagnostics, and out-of-sample validation for age-standardized IBD, UC, and CD burden measures in Asia.	53](#_Toc23943)

[S21 Table. Sex-specific age-standardized DALY and mortality rates of IBD, UC, and CD across Asia and Asian subregions in 2023.	54](#_Toc1109)

[S22 Table. Comparison of temporal trends in age-standardized burden measures between ulcerative colitis and Crohn’s disease in Asia and its subregions, 1990–2023.	55](#_Toc25321)

[S23 Table. Temporal changes in UC-to-CD ratios for age-standardized incidence, prevalence, DALY, and mortality rates in Asia and its subregions, 1990–2023.	56](#_Toc6624)

[S24 Table. Sex-specific age-standardized incidence, prevalence, DALY, and mortality rates for IBD, UC, and CD across Asia and its subregions, 2023.	58](#_Toc22995)

**S1 Fig: Age-standardized incidence rate (A), DALY rate (B), prevalence rate (C), and mortality rate (D) of UC per 100,000 population in Asia, 2023.** Base map data were obtained from Natural Earth (public domain). The figure was generated by the authors using original R code.

**S2 Fig: Age-standardized incidence rate (E), DALY rate (F), prevalence rate (G), and mortality rate (H) of CD per 100,000 population in Asia, 2023.** Base map data were obtained from Natural Earth (public domain). The figure was generated by the authors using original R code.

**S3 Fig. Observed versus predicted IBD ASIR, ASPR, ASDR, and ASMR in Asia, 2020-2023.**

The ARIMA models were fitted using data from 1990 to 2019 and validated against observed estimates for 2020-2023. Observed values are shown by the solid black line for the full series and by blue points for the validation period. Predicted values are shown by the dashed orange line, and the shaded band with dashed boundaries indicates the 95% prediction interval. ASIR, age-standardized incidence rate; ASPR, age-standardized prevalence rate; ASDR, age-standardized DALY rate; ASMR, age-standardized mortality rate.

**S4 Fig. Temporal trends in age-standardized burden measures for ulcerative colitis and Crohn’s disease across Asia and its subregions, 1990–2023.**

**S5 Fig. Age-specific prevalence rates of Crohn’s disease across Asian subregions in 2023.**

**S6 Fig. Temporal trends in UC-to-CD ratios for age-standardized burden measures across Asia and its subregions, 1990–2023.The UC-to-CD ratio was calculated as the age-standardized UC rate divided by the corresponding age-standardized CD rate.** Shaded areas represent approximate uncertainty ranges based on the country-level lower and upper estimates.

**S7 Fig. Male-to-female ratios of age-standardized incidence, prevalence, DALY, and mortality rates for IBD, UC, and CD across Asia and its five subregions in 2023.** A ratio greater than 1 indicates a higher rate in males, whereas a ratio below 1 indicates a higher rate in females.

**S1 Table: Incidence of IBD in 1990 and 2023, and estimated annual percentage changes from 1990 to 2023.**

| Countries | Number of cases, 1990 | Age-standardised rate per 100 000 population, 1990 | Number of cases, 2023 | Age-standardised rate per 100 000 population, 2023 | Estimated annual percentage change, 1990–2023 |
| --- | --- | --- | --- | --- | --- |
| China | 8451.711(7236.454-10197.571) | 0.717(0.614-0.865) | 25482.447(21467.279-30992.165) | 1.781(1.501-2.166) | 3.47(2.87,4.07) |
| Democratic People's Republic of Korea | 145.922(124.080-176.915) | 0.699(0.595-0.848) | 298.927(245.653-367.578) | 1.132(0.930-1.392) | 1.25(1.13,1.37) |
| Cambodia | 40.863(34.777-49.885) | 0.396(0.337-0.484) | 106.668(89.380-131.819) | 0.620(0.520-0.767) | 1.57(1.51,1.63) |
| Indonesia | 998.837(853.121-1219.810) | 0.538(0.459-0.657) | 2174.944(1822.292-2658.606) | 0.757(0.634-0.925) | 0.95(0.90,1.00) |
| Lao People's Democratic Republic | 16.782(14.371-20.399) | 0.402(0.344-0.489) | 45.610(38.595-55.863) | 0.606(0.513-0.743) | 1.44(1.37,1.51) |
| Malaysia | 88.937(78.071-105.632) | 0.502(0.441-0.597) | 289.964(247.144-349.541) | 0.849(0.723-1.023) | 2.09(1.84,2.34) |
| Maldives | 0.798(0.684-0.954) | 0.359(0.308-0.429) | 3.689(3.114-4.520) | 0.680(0.574-0.833) | 2.30(2.17,2.44) |
| Myanmar | 190.067(159.896-226.753) | 0.470(0.395-0.560) | 380.234(323.483-463.358) | 0.661(0.562-0.806) | 1.11(1.07,1.14) |
| Philippines | 225.741(189.987-280.749) | 0.357(0.300-0.444) | 536.791(446.729-664.805) | 0.469(0.390-0.581) | 1.00(0.93,1.07) |
| Sri Lanka | 114.197(95.252-137.969) | 0.669(0.558-0.808) | 231.162(195.609-281.401) | 1.020(0.863-1.242) | 1.50(1.22,1.79) |
| Thailand | 277.300(235.624-331.362) | 0.486(0.413-0.581) | 477.916(400.469-575.543) | 0.727(0.609-0.876) | 1.15(1.08,1.21) |
| Timor-Leste | 3.103(2.598-3.791) | 0.397(0.333-0.485) | 7.511(6.387-9.075) | 0.524(0.445-0.632) | 0.84(0.82,0.86) |
| Viet Nam | 286.866(241.354-348.770) | 0.419(0.352-0.509) | 690.180(577.379-862.172) | 0.669(0.560-0.836) | 1.48(1.37,1.60) |
| Armenia | 130.414(110.929-159.646) | 3.800(3.232-4.652) | 185.446(153.240-229.798) | 6.117(5.055-7.580) | 1.51(1.44,1.58) |
| Azerbaijan | 310.765(257.071-376.201) | 4.223(3.493-5.112) | 640.980(518.646-793.218) | 6.092(4.929-7.539) | 1.27(1.20,1.33) |
| Georgia | 279.449(231.826-342.280) | 5.041(4.182-6.175) | 214.566(177.012-266.433) | 5.992(4.943-7.440) | 0.52(0.46,0.58) |
| Kazakhstan | 645.743(542.976-780.484) | 3.810(3.204-4.605) | 1093.559(897.748-1323.516) | 5.360(4.400-6.487) | 0.61(0.40,0.81) |
| Kyrgyzstan | 160.583(134.851-195.242) | 3.587(3.012-4.361) | 298.756(245.621-368.434) | 4.121(3.388-5.082) | 0.32(0.26,0.38) |
| Mongolia | 65.504(56.270-79.400) | 3.027(2.601-3.670) | 179.047(147.046-220.073) | 5.171(4.247-6.356) | 1.83(1.70,1.97) |
| Tajikistan | 161.548(138.265-192.202) | 3.006(2.573-3.577) | 414.659(346.035-500.598) | 3.880(3.238-4.684) | 0.90(0.83,0.98) |
| Turkmenistan | 134.078(114.013-162.281) | 3.610(3.070-4.370) | 288.492(241.043-346.287) | 5.143(4.297-6.174) | 1.21(1.16,1.26) |
| Uzbekistan | 588.528(502.560-706.314) | 2.823(2.410-3.388) | 1767.992(1478.001-2150.049) | 5.002(4.182-6.083) | 2.03(1.89,2.17) |
| Brunei Darussalam | 1.977(1.695-2.401) | 0.758(0.650-0.921) | 4.081(3.474-4.985) | 0.887(0.755-1.084) | 0.51(0.45,0.58) |
| Japan | 2697.634(2253.620-3278.270) | 2.181(1.822-2.650) | 3101.094(2635.686-3779.257) | 2.487(2.114-3.031) | 0.08(-0.22,0.39) |
| Republic of Korea | 1610.550(1379.338-1906.211) | 3.614(3.095-4.277) | 3573.819(3109.144-4278.194) | 6.913(6.014-8.275) | 2.42(1.90,2.94) |
| Singapore | 16.886(14.446-20.250) | 0.554(0.474-0.665) | 47.058(39.764-56.288) | 0.827(0.699-0.989) | 1.31(1.17,1.46) |
| Cyprus | 57.149(49.026-67.286) | 7.362(6.315-8.668) | 163.947(139.700-191.301) | 11.984(10.212-13.984) | 1.49(1.31,1.67) |
| Israel | 499.312(435.877-607.466) | 10.008(8.736-12.175) | 1314.490(1122.073-1579.854) | 13.158(11.232-15.815) | 0.22(-0.19,0.63) |
| Bahrain | 10.406(9.023-12.297) | 2.042(1.771-2.413) | 43.381(36.277-52.362) | 2.770(2.316-3.343) | 1.02(0.92,1.12) |
| Iran (Islamic Republic of) | 1159.716(973.603-1444.479) | 2.031(1.705-2.529) | 2891.141(2355.495-3656.099) | 3.288(2.679-4.158) | 1.95(1.69,2.21) |
| Iraq | 262.380(229.036-310.855) | 1.414(1.235-1.676) | 1103.884(946.009-1328.460) | 2.499(2.141-3.007) | 1.74(1.71,1.76) |
| Jordan | 76.755(67.168-87.932) | 2.042(1.787-2.339) | 433.658(366.584-527.047) | 3.285(2.777-3.993) | 1.40(1.30,1.50) |
| Kuwait | 51.963(46.741-58.901) | 3.053(2.746-3.460) | 165.745(136.380-207.659) | 3.542(2.914-4.437) | 0.44(0.06,0.83) |
| Lebanon | 187.926(160.188-234.036) | 5.911(5.039-7.362) | 434.933(366.042-529.965) | 7.624(6.417-9.290) | 1.10(0.95,1.26) |
| Palestine | 34.126(29.434-41.234) | 1.657(1.429-2.002) | 145.915(123.134-179.800) | 2.698(2.277-3.325) | 1.65(1.55,1.75) |
| Oman | 36.315(30.768-44.323) | 1.818(1.541-2.219) | 191.428(158.071-239.466) | 3.728(3.079-4.664) | 2.46(2.34,2.58) |
| Qatar | 11.853(9.733-14.644) | 2.638(2.166-3.259) | 137.065(109.246-171.321) | 4.417(3.521-5.522) | 1.79(1.67,1.91) |
| Saudi Arabia | 285.728(246.777-345.745) | 1.758(1.519-2.128) | 873.730(745.801-1061.567) | 2.649(2.261-3.218) | 1.14(1.06,1.23) |
| Syrian Arab Republic | 226.487(194.143-271.665) | 1.778(1.524-2.133) | 554.123(467.822-676.342) | 3.116(2.631-3.804) | 1.83(1.62,2.04) |
| Türkiye | 1289.483(1103.167-1550.625) | 2.244(1.920-2.699) | 2925.492(2474.048-3524.140) | 3.407(2.881-4.104) | 1.30(1.01,1.60) |
| United Arab Emirates | 53.898(45.793-67.605) | 2.863(2.432-3.591) | 521.955(403.044-671.686) | 5.152(3.978-6.630) | 1.79(1.76,1.82) |
| Afghanistan | 170.398(147.078-202.347) | 1.661(1.433-1.972) | 703.217(601.076-854.961) | 1.940(1.659-2.359) | 0.75(0.56,0.94) |
| Bangladesh | 3401.207(2900.189-4094.743) | 3.118(2.659-3.754) | 8599.884(7044.675-10498.003) | 4.947(4.052-6.038) | 1.51(1.45,1.57) |
| Bhutan | 19.665(16.654-23.989) | 3.181(2.694-3.881) | 45.993(38.245-56.508) | 6.162(5.124-7.571) | 2.04(1.91,2.18) |
| India | 31927.504(26800.975-38603.534) | 3.777(3.170-4.567) | 84475.244(71056.342-102922.167) | 5.853(4.923-7.131) | 1.48(1.39,1.56) |
| Nepal | 630.596(525.817-753.322) | 3.242(2.703-3.872) | 1626.190(1359.043-1962.164) | 5.235(4.375-6.317) | 1.62(1.58,1.66) |
| Pakistan | 3541.949(3013.938-4266.154) | 3.181(2.707-3.831) | 11110.536(9358.413-13552.328) | 4.554(3.836-5.555) | 0.95(0.85,1.05) |

**S2 Table: DALYs of IBD in 1990 and 2023, and estimated annual percentage changes from 1990 to 2023.**

| Countries | Number of cases, 1990 | Age-standardised rate per 100 000 population, 1990 | Number of cases, 2023 | Age-standardised rate per 100 000 population, 2023 | Estimated annual percentage change, 1990–2023 |
| --- | --- | --- | --- | --- | --- |
| China | 138192.948(75583.106-214404.379) | 11.720(6.410-18.183) | 123134.361(95812.242-159259.984) | 8.607(6.697-11.132) | -1.13(-1.33,-0.92) |
| Democratic People’s Republic of Korea | 2055.281(1251.092-3119.534) | 9.849(5.995-14.949) | 2301.389(1273.205-3739.893) | 8.714(4.821-14.161) | -0.10(-0.22,0.02) |
| Cambodia | 646.830(273.234-1239.444) | 6.275(2.651-12.024) | 1005.778(533.438-1669.526) | 5.850(3.103-9.711) | -0.25(-0.46,-0.04) |
| Indonesia | 18272.865(10026.176-30479.892) | 9.840(5.399-16.414) | 27309.547(16586.421-41816.932) | 9.500(5.770-14.547) | -0.36(-0.47,-0.25) |
| Lao People’s Democratic Republic | 175.842(89.896-320.978) | 4.213(2.154-7.690) | 304.508(192.233-479.287) | 4.048(2.556-6.372) | -0.26(-0.50,-0.02) |
| Malaysia | 1799.121(838.803-3127.152) | 10.163(4.738-17.664) | 3454.419(1654.171-5553.480) | 10.111(4.842-16.255) | 0.57(0.10,1.03) |
| Maldives | 11.260(6.459-19.241) | 5.065(2.905-8.656) | 31.778(21.744-46.865) | 5.856(4.007-8.637) | 0.64(0.43,0.85) |
| Myanmar | 4299.865(1911.258-7928.995) | 10.623(4.722-19.589) | 5926.959(2936.163-10368.464) | 10.306(5.105-18.028) | -0.14(-0.28,0.01) |
| Philippines | 3425.323(2121.118-5591.805) | 5.416(3.354-8.842) | 6082.528(4442.383-8301.374) | 5.312(3.879-7.249) | -0.26(-0.50,-0.02) |
| Sri Lanka | 417.189(308.219-579.875) | 2.443(1.805-3.395) | 670.808(502.538-857.014) | 2.961(2.218-3.783) | 0.74(0.59,0.90) |
| Thailand | 1322.277(921.547-1951.912) | 2.317(1.615-3.420) | 3305.281(2470.051-4559.611) | 5.029(3.758-6.938) | 2.26(2.06,2.46) |
| Timor-Leste | 55.799(25.977-106.156) | 7.145(3.326-13.594) | 92.861(52.816-149.055) | 6.472(3.681-10.389) | -0.46(-0.76,-0.15) |
| Viet Nam | 3290.830(1865.963-5757.471) | 4.801(2.722-8.400) | 5941.725(3829.111-9221.821) | 5.760(3.712-8.940) | 0.78(0.67,0.88) |
| Armenia | 752.357(601.650-899.591) | 21.923(17.531-26.213) | 532.245(416.903-674.802) | 17.556(13.752-22.258) | -0.10(-0.30,0.10) |
| Azerbaijan | 845.188(632.851-1133.524) | 11.485(8.599-15.402) | 1450.290(1049.336-1925.598) | 13.783(9.973-18.300) | 0.73(0.56,0.89) |
| Georgia | 1806.525(1394.240-2367.226) | 32.591(25.153-42.706) | 673.730(532.549-839.270) | 18.814(14.871-23.437) | -1.68(-2.32,-1.04) |
| Kazakhstan | 3817.137(3170.458-4604.646) | 22.523(18.707-27.169) | 4333.558(3586.658-5101.073) | 21.240(17.580-25.002) | 0.12(-0.13,0.37) |
| Kyrgyzstan | 1402.854(1141.716-1739.572) | 31.333(25.501-38.854) | 921.673(726.566-1158.421) | 12.712(10.021-15.977) | -3.38(-4.08,-2.69) |
| Mongolia | 480.082(295.370-759.197) | 22.188(13.651-35.088) | 595.548(432.873-799.376) | 17.201(12.503-23.088) | -1.04(-1.19,-0.89) |
| Tajikistan | 738.761(520.387-1031.828) | 13.748(9.684-19.201) | 1718.444(1201.555-2362.232) | 16.081(11.244-22.105) | 0.44(0.06,0.81) |
| Turkmenistan | 608.718(474.368-777.654) | 16.391(12.773-20.940) | 1065.332(810.659-1375.829) | 18.993(14.453-24.529) | 1.47(1.13,1.81) |
| Uzbekistan | 3727.737(3091.187-4533.809) | 17.880(14.826-21.746) | 5701.364(4696.180-6806.054) | 16.131(13.287-19.257) | -0.04(-0.43,0.36) |
| Brunei Darussalam | 32.112(20.716-48.346) | 12.310(7.941-18.533) | 66.536(46.513-91.466) | 14.468(10.114-19.889) | 0.58(0.30,0.87) |
| Japan | 12844.546(10618.286-15700.902) | 10.385(8.585-12.694) | 17620.110(14043.608-21549.142) | 14.133(11.264-17.285) | 0.76(0.52,1.01) |
| Republic of Korea | 9567.675(5863.243-12974.239) | 21.470(13.157-29.114) | 10109.487(7182.184-13926.614) | 19.555(13.892-26.938) | -0.37(-0.56,-0.18) |
| Singapore | 133.838(115.208-157.764) | 4.392(3.781-5.177) | 207.314(165.661-256.672) | 3.642(2.910-4.509) | -1.15(-1.50,-0.79) |
| Cyprus | 331.888(229.208-474.397) | 42.753(29.526-61.111) | 596.219(457.197-770.257) | 43.582(33.420-56.304) | -0.05(-0.16,0.05) |
| Israel | 1219.842(907.957-1567.055) | 24.449(18.198-31.409) | 3034.417(2284.639-4062.444) | 30.375(22.870-40.666) | 0.24(-0.19,0.67) |
| Bahrain | 42.797(29.717-63.839) | 8.399(5.832-12.529) | 172.828(124.685-232.060) | 11.035(7.961-14.817) | 0.69(0.60,0.79) |
| Iran (Islamic Republic of) | 7623.421(4988.312-11729.925) | 13.349(8.735-20.539) | 12387.583(9022.878-16491.301) | 14.089(10.262-18.756) | 0.05(-0.11,0.21) |
| Iraq | 1917.584(1199.335-3159.178) | 10.338(6.466-17.031) | 5013.918(3541.006-6824.019) | 11.349(8.015-15.447) | 0.26(0.14,0.37) |
| Jordan | 303.971(210.460-435.453) | 8.085(5.598-11.583) | 1408.547(1053.154-1891.656) | 10.671(7.978-14.331) | 1.15(0.98,1.33) |
| Kuwait | 169.282(128.254-208.625) | 9.945(7.535-12.257) | 468.242(344.192-652.739) | 10.006(7.355-13.948) | -0.16(-0.97,0.67) |
| Lebanon | 923.186(596.501-1361.970) | 29.038(18.763-42.840) | 1746.967(1325.390-2250.036) | 30.624(23.234-39.443) | 0.06(-0.06,0.17) |
| Palestine | 366.349(234.474-570.561) | 17.790(11.386-27.707) | 700.594(523.624-917.245) | 12.954(9.682-16.960) | -1.06(-1.32,-0.81) |
| Oman | 151.432(98.454-248.259) | 7.583(4.930-12.431) | 610.379(440.642-842.081) | 11.888(8.582-16.401) | 1.35(1.21,1.48) |
| Qatar | 45.179(30.464-67.849) | 10.056(6.780-15.101) | 355.218(253.390-493.402) | 11.448(8.167-15.902) | 0.01(-0.12,0.14) |
| Saudi Arabia | 693.752(507.809-955.142) | 4.270(3.125-5.878) | 2457.354(1671.946-3302.489) | 7.449(5.068-10.011) | 1.48(1.27,1.70) |
| Syrian Arab Republic | 1238.771(816.102-1909.997) | 9.726(6.407-14.995) | 1919.502(1391.594-2520.345) | 10.795(7.826-14.174) | 0.23(0.06,0.41) |
| Türkiye | 9180.777(5934.358-13419.831) | 15.978(10.328-23.355) | 14298.648(11242.084-17772.222) | 16.652(13.093-20.698) | 0.13(0.05,0.20) |
| United Arab Emirates | 88.527(54.766-127.812) | 4.702(2.909-6.789) | 1038.615(690.449-1503.767) | 10.252(6.815-14.843) | 2.19(2.04,2.34) |
| Afghanistan | 704.822(435.762-1118.961) | 6.868(4.246-10.904) | 2357.834(1606.820-3430.917) | 6.506(4.434-9.467) | -0.01(-0.30,0.28) |
| Bangladesh | 20523.744(13868.909-30813.894) | 18.815(12.714-28.248) | 37414.877(23363.092-51078.395) | 21.520(13.438-29.379) | 0.48(0.35,0.61) |
| Bhutan | 98.202(59.725-167.467) | 15.885(9.661-27.090) | 151.187(91.229-232.124) | 20.257(12.223-31.101) | 0.44(0.33,0.55) |
| India | 114752.533(81289.036-162375.296) | 13.574(9.616-19.208) | 213841.914(154241.187-278909.708) | 14.815(10.686-19.323) | 0.15(0.06,0.24) |
| Nepal | 3650.725(2267.272-5887.880) | 18.767(11.655-30.267) | 5588.455(3835.803-7876.104) | 17.990(12.348-25.355) | -0.27(-0.56,0.02) |
| Pakistan | 17352.029(10409.039-27915.357) | 15.582(9.347-25.068) | 38220.756(24387.620-58502.694) | 15.667(9.997-23.981) | -0.28(-0.37,-0.19) |

**S3 Table: Prevalence of IBD in 1990 and 2023, and estimated annual percentage changes from 1990 to 2023.**

| Countries | Number of cases, 1990 | Age-standardised rate per 100 000 population, 1990 | Number of cases, 2023 | Age-standardised rate per 100 000 population, 2023 | Estimated annual percentage change, 1990–2023 |
| --- | --- | --- | --- | --- | --- |
| China | 63850.039(54750.512-74894.670) | 5.415(4.643-6.352) | 175898.921(151240.213-207405.061) | 12.295(10.571-14.497) | 3.18(2.51,3.85) |
| Democratic People's Republic of Korea | 1508.222(1258.466-1807.198) | 7.228(6.031-8.660) | 1979.675(1665.643-2403.677) | 7.496(6.307-9.101) | 0.28(0.12,0.45) |
| Cambodia | 332.645(276.728-397.143) | 3.227(2.685-3.853) | 861.767(715.236-1011.632) | 5.013(4.160-5.884) | 1.38(1.31,1.44) |
| Indonesia | 7998.988(6699.031-9459.239) | 4.308(3.608-5.094) | 17674.598(15135.953-21024.354) | 6.148(5.265-7.314) | 0.98(0.93,1.03) |
| Lao People's Democratic Republic | 136.311(114.109-161.929) | 3.266(2.734-3.879) | 373.827(308.942-441.820) | 4.970(4.107-5.874) | 1.41(1.29,1.53) |
| Malaysia | 969.304(843.653-1130.297) | 5.475(4.766-6.385) | 3072.436(2624.790-3576.990) | 8.993(7.683-10.470) | 1.96(1.67,2.25) |
| Maldives | 7.881(6.619-9.333) | 3.545(2.978-4.199) | 42.962(36.175-50.734) | 7.918(6.667-9.350) | 2.53(2.49,2.56) |
| Myanmar | 1572.300(1293.150-1871.440) | 3.884(3.195-4.624) | 3069.893(2602.667-3627.432) | 5.338(4.525-6.307) | 0.95(0.91,0.99) |
| Philippines | 2248.240(1892.243-2665.619) | 3.555(2.992-4.215) | 4972.894(4207.516-5880.836) | 4.343(3.674-5.136) | 0.52(0.45,0.59) |
| Sri Lanka | 1136.469(936.699-1345.603) | 6.654(5.484-7.878) | 2340.563(1998.618-2778.643) | 10.331(8.822-12.265) | 1.47(1.23,1.71) |
| Thailand | 2705.422(2288.026-3193.516) | 4.740(4.009-5.596) | 5234.320(4451.226-6112.632) | 7.964(6.773-9.300) | 1.51(1.48,1.55) |
| Timor-Leste | 27.004(21.904-32.164) | 3.458(2.805-4.119) | 61.620(52.938-72.856) | 4.295(3.690-5.078) | 0.51(0.37,0.65) |
| Viet Nam | 3032.135(2559.188-3599.527) | 4.424(3.734-5.252) | 7667.268(6648.004-8916.473) | 7.433(6.445-8.644) | 1.66(1.58,1.73) |
| Armenia | 1531.270(1306.320-1832.405) | 44.620(38.065-53.394) | 2062.816(1740.095-2440.270) | 68.042(57.397-80.493) | 1.33(1.30,1.37) |
| Azerbaijan | 2736.174(2234.096-3277.737) | 37.179(30.357-44.538) | 5865.122(4905.467-6997.970) | 55.741(46.620-66.507) | 1.45(1.33,1.57) |
| Georgia | 3117.565(2604.069-3759.675) | 56.242(46.979-67.826) | 2478.770(2067.319-2940.365) | 69.220(57.730-82.110) | 0.72(0.64,0.79) |
| Kazakhstan | 7327.945(6198.965-8716.249) | 43.238(36.576-51.430) | 10556.191(8921.005-12517.953) | 51.740(43.725-61.355) | 0.88(0.76,1.00) |
| Kyrgyzstan | 1456.465(1218.161-1731.908) | 32.531(27.208-38.683) | 3157.520(2653.817-3781.271) | 43.550(36.603-52.153) | 0.95(0.87,1.03) |
| Mongolia | 563.125(478.257-659.885) | 26.026(22.104-30.498) | 1407.298(1185.883-1676.430) | 40.647(34.252-48.420) | 1.55(1.44,1.67) |
| Tajikistan | 1423.816(1174.737-1776.464) | 26.496(21.861-33.058) | 3664.549(3059.694-4298.036) | 34.292(28.632-40.220) | 0.99(0.88,1.10) |
| Turkmenistan | 1060.888(903.685-1237.771) | 28.567(24.334-33.330) | 2406.126(2001.078-2859.051) | 42.897(35.676-50.972) | 1.36(1.31,1.42) |
| Uzbekistan | 6440.022(5484.855-7609.002) | 30.889(26.307-36.496) | 16463.447(13760.438-20232.818) | 46.581(38.933-57.246) | 1.34(1.29,1.39) |
| Brunei Darussalam | 21.641(18.129-25.524) | 8.296(6.950-9.784) | 57.809(48.479-68.792) | 12.570(10.541-14.958) | 1.28(1.25,1.31) |
| Japan | 38227.467(32570.338-45318.243) | 30.907(26.333-36.639) | 55873.989(48016.680-66651.328) | 44.817(38.514-53.461) | 0.84(0.38,1.31) |
| Republic of Korea | 16649.025(14269.702-19323.636) | 37.360(32.021-43.362) | 43180.570(37289.288-50884.801) | 83.523(72.128-98.426) | 2.86(2.17,3.55) |
| Singapore | 207.488(175.806-242.834) | 6.809(5.770-7.969) | 616.692(525.930-727.342) | 10.835(9.240-12.779) | 1.48(1.24,1.72) |
| Cyprus | 765.500(653.283-890.860) | 98.610(84.155-114.759) | 1776.424(1514.565-2029.721) | 129.852(110.711-148.368) | 0.68(0.55,0.81) |
| Israel | 6060.524(5182.617-7060.626) | 121.471(103.875-141.516) | 14145.569(12114.225-16466.349) | 141.601(121.267-164.833) | -0.23(-0.72,0.27) |
| Bahrain | 120.658(99.971-149.721) | 23.680(19.620-29.384) | 645.628(553.947-782.060) | 41.224(35.370-49.935) | 1.90(1.73,2.07) |
| Iran (Islamic Republic of) | 12061.060(10188.125-14494.952) | 21.119(17.840-25.381) | 35769.856(30061.707-43699.963) | 40.682(34.190-49.701) | 2.20(2.00,2.40) |
| Iraq | 2344.881(2001.158-2746.997) | 12.641(10.788-14.809) | 11005.987(9351.011-12841.804) | 24.913(21.167-29.069) | 1.62(1.49,1.75) |
| Jordan | 870.882(752.926-1009.800) | 23.165(20.027-26.860) | 5683.069(4814.544-6730.489) | 43.053(36.474-50.988) | 1.99(1.89,2.10) |
| Kuwait | 606.926(535.704-680.631) | 35.657(31.473-39.987) | 2240.127(1913.953-2704.854) | 47.869(40.899-57.799) | 0.60(0.16,1.03) |
| Lebanon | 2076.317(1711.437-2534.255) | 65.310(53.833-79.714) | 6294.137(5364.203-7388.013) | 110.336(94.035-129.512) | 1.17(0.96,1.38) |
| Palestine | 326.539(272.022-391.711) | 15.857(13.210-19.022) | 1546.350(1293.528-1844.939) | 28.593(23.918-34.114) | 1.51(1.42,1.60) |
| Oman | 350.183(293.495-415.157) | 17.535(14.696-20.789) | 2083.736(1721.705-2563.866) | 40.585(33.534-49.937) | 2.33(2.24,2.42) |
| Qatar | 137.041(113.091-169.888) | 30.502(25.171-37.813) | 1696.642(1416.477-2012.330) | 54.681(45.652-64.855) | 1.50(1.38,1.62) |
| Saudi Arabia | 2482.354(2093.506-2945.226) | 15.277(12.884-18.126) | 9492.797(8094.347-11243.821) | 28.776(24.537-34.084) | 1.63(1.48,1.78) |
| Syrian Arab Republic | 2010.585(1687.274-2463.947) | 15.785(13.247-19.345) | 6015.828(5029.399-7217.142) | 33.832(28.284-40.588) | 2.11(2.03,2.19) |
| Türkiye | 12514.989(10584.659-14877.184) | 21.780(18.421-25.891) | 37925.502(32699.493-44602.500) | 44.168(38.082-51.945) | 2.15(1.75,2.55) |
| United Arab Emirates | 508.912(424.125-619.331) | 27.032(22.529-32.898) | 6596.462(5429.651-7990.754) | 65.111(53.594-78.874) | 2.40(2.25,2.56) |
| Afghanistan | 1363.973(1137.671-1617.283) | 13.292(11.087-15.760) | 5847.506(4821.613-7164.328) | 16.135(13.304-19.768) | 0.40(0.17,0.63) |
| Bangladesh | 27553.302(23453.818-32599.286) | 25.259(21.501-29.885) | 87814.067(73166.952-103931.858) | 50.509(42.084-59.780) | 2.29(2.17,2.40) |
| Bhutan | 154.200(131.981-181.621) | 24.944(21.349-29.379) | 366.996(307.722-435.168) | 49.173(41.231-58.307) | 2.01(1.90,2.11) |
| India | 239353.111(201820.143-281394.928) | 28.314(23.874-33.287) | 699554.343(595689.435-828115.286) | 48.466(41.270-57.373) | 1.56(1.47,1.66) |
| Nepal | 5156.979(4325.345-6151.918) | 26.510(22.234-31.624) | 14068.036(11792.224-16610.844) | 45.288(37.962-53.474) | 1.60(1.44,1.75) |
| Pakistan | 27304.210(23099.653-32684.459) | 24.519(20.743-29.350) | 73783.458(61993.616-87640.173) | 30.245(25.412-35.925) | 0.59(0.46,0.72) |

**S4 Table: Deaths of IBD in 1990 and 2023, and estimated annual percentage changes from 1990 to 2023.**

| Countries | Number of cases, 1990 | Age-standardised rate per 100 000 population, 1990 | Number of cases, 2023 | Age-standardised rate per 100 000 population, 2023 | Estimated annual percentage change, 1990–2023 |
| --- | --- | --- | --- | --- | --- |
| China | 3799.400(2075.224-5945.988) | 0.322(0.176-0.504) | 4887.219(3505.643-6720.602) | 0.342(0.245-0.470) | -0.30(-0.62,0.01) |
| Democratic People's Republic of Korea | 52.529(28.800-88.456) | 0.252(0.138-0.424) | 81.037(39.241-140.514) | 0.307(0.149-0.532) | 0.96(0.79,1.13) |
| Cambodia | 12.765(4.986-25.191) | 0.124(0.048-0.244) | 29.483(13.321-52.259) | 0.171(0.077-0.304) | 1.13(0.95,1.32) |
| Indonesia | 506.314(281.262-878.494) | 0.273(0.151-0.473) | 850.768(500.704-1325.105) | 0.296(0.174-0.461) | -0.06(-0.18,0.07) |
| Lao People's Democratic Republic | 4.073(2.020-7.654) | 0.098(0.048-0.183) | 7.869(4.370-13.328) | 0.105(0.058-0.177) | 0.20(-0.07,0.46) |
| Malaysia | 54.374(22.120-95.805) | 0.307(0.125-0.541) | 122.439(45.449-215.029) | 0.358(0.133-0.629) | 1.05(0.50,1.61) |
| Maldives | 0.240(0.126-0.427) | 0.108(0.057-0.192) | 0.917(0.562-1.484) | 0.169(0.104-0.274) | 1.73(1.56,1.89) |
| Myanmar | 89.276(39.291-163.870) | 0.221(0.097-0.405) | 130.702(59.258-226.902) | 0.227(0.103-0.395) | -0.00(-0.14,0.13) |
| Philippines | 75.189(44.145-127.440) | 0.119(0.070-0.202) | 158.769(112.459-221.647) | 0.139(0.098-0.194) | 0.18(-0.07,0.44) |
| Sri Lanka | 7.281(4.804-11.264) | 0.043(0.028-0.066) | 13.936(8.603-20.444) | 0.062(0.038-0.090) | 1.12(0.78,1.46) |
| Thailand | 26.549(13.828-45.840) | 0.047(0.024-0.080) | 110.266(70.608-159.846) | 0.168(0.107-0.243) | 3.96(3.64,4.29) |
| Timor-Leste | 1.061(0.480-2.072) | 0.136(0.061-0.265) | 2.725(1.440-4.767) | 0.190(0.100-0.332) | 1.00(0.81,1.19) |
| Viet Nam | 92.128(47.552-164.750) | 0.134(0.069-0.240) | 198.772(113.747-318.825) | 0.193(0.110-0.309) | 1.38(1.27,1.48) |
| Armenia | 14.536(11.915-17.838) | 0.424(0.347-0.520) | 8.473(7.019-10.066) | 0.279(0.232-0.332) | -0.32(-0.71,0.07) |
| Azerbaijan | 9.991(6.236-15.332) | 0.136(0.085-0.208) | 14.355(9.123-22.692) | 0.136(0.087-0.216) | 0.14(-0.08,0.37) |
| Georgia | 41.064(28.444-58.282) | 0.741(0.513-1.051) | 11.028(8.788-13.709) | 0.308(0.245-0.383) | -2.75(-4.04,-1.45) |
| Kazakhstan | 67.377(55.793-81.739) | 0.398(0.329-0.482) | 81.217(67.685-97.057) | 0.398(0.332-0.476) | 0.34(0.03,0.64) |
| Kyrgyzstan | 25.869(20.383-33.292) | 0.578(0.455-0.744) | 10.478(8.798-12.337) | 0.145(0.121-0.170) | -5.21(-6.20,-4.20) |
| Mongolia | 7.735(4.051-12.331) | 0.357(0.187-0.570) | 10.312(6.660-15.242) | 0.298(0.192-0.440) | -1.14(-1.42,-0.87) |
| Tajikistan | 12.386(7.352-20.182) | 0.230(0.137-0.376) | 25.935(15.629-40.934) | 0.243(0.146-0.383) | -0.27(-0.76,0.22) |
| Turkmenistan | 8.783(6.780-11.971) | 0.237(0.183-0.322) | 16.126(11.276-22.888) | 0.288(0.201-0.408) | 1.50(1.13,1.88) |
| Uzbekistan | 54.563(43.352-68.823) | 0.262(0.208-0.330) | 73.395(62.658-86.748) | 0.208(0.177-0.245) | -0.52(-1.27,0.23) |
| Brunei Darussalam | 0.801(0.493-1.199) | 0.307(0.189-0.459) | 2.017(1.315-2.920) | 0.438(0.286-0.635) | 1.17(0.82,1.51) |
| Japan | 334.598(281.464-397.296) | 0.271(0.228-0.321) | 607.146(474.087-735.999) | 0.487(0.380-0.590) | 1.97(1.33,2.62) |
| Republic of Korea | 257.191(111.371-396.823) | 0.577(0.250-0.890) | 217.543(136.781-412.209) | 0.421(0.265-0.797) | -2.23(-2.63,-1.84) |
| Singapore | 3.505(2.939-4.155) | 0.115(0.096-0.136) | 5.865(4.756-7.111) | 0.103(0.084-0.125) | -2.08(-2.96,-1.19) |
| Cyprus | 12.473(6.831-20.735) | 1.607(0.880-2.671) | 21.306(14.391-32.987) | 1.557(1.052-2.411) | -0.14(-0.29,0.01) |
| Israel | 14.831(12.605-17.351) | 0.297(0.253-0.348) | 58.598(47.197-69.131) | 0.587(0.472-0.692) | 2.30(1.83,2.78) |
| Bahrain | 0.590(0.301-1.126) | 0.116(0.059-0.221) | 2.070(1.238-3.093) | 0.132(0.079-0.197) | -0.29(-0.51,-0.07) |
| Iran (Islamic Republic of) | 138.184(83.825-219.413) | 0.242(0.147-0.384) | 256.083(137.150-428.589) | 0.291(0.156-0.487) | 0.22(-0.04,0.49) |
| Iraq | 31.841(16.360-59.816) | 0.172(0.088-0.322) | 80.577(44.233-128.913) | 0.182(0.100-0.292) | 0.34(0.16,0.52) |
| Jordan | 4.359(2.179-8.218) | 0.116(0.058-0.219) | 16.483(9.593-24.905) | 0.125(0.073-0.189) | 0.77(0.42,1.13) |
| Kuwait | 1.779(1.279-2.325) | 0.105(0.075-0.137) | 4.089(3.234-5.060) | 0.087(0.069-0.108) | -0.40(-2.37,1.61) |
| Lebanon | 20.936(10.063-34.757) | 0.659(0.317-1.093) | 32.866(21.085-48.453) | 0.576(0.370-0.849) | -0.31(-0.59,-0.02) |
| Palestine | 10.193(5.829-16.931) | 0.495(0.283-0.822) | 14.631(9.430-21.092) | 0.271(0.174-0.390) | -1.98(-2.32,-1.64) |
| Oman | 2.242(1.157-4.335) | 0.112(0.058-0.217) | 7.835(4.424-12.750) | 0.153(0.086-0.248) | 1.07(0.78,1.36) |
| Qatar | 0.603(0.281-1.157) | 0.134(0.062-0.258) | 2.500(1.504-3.746) | 0.081(0.048-0.121) | -2.21(-2.50,-1.92) |
| Saudi Arabia | 6.288(3.525-10.340) | 0.039(0.022-0.064) | 24.314(10.198-40.201) | 0.074(0.031-0.122) | 1.84(1.39,2.29) |
| Syrian Arab Republic | 22.403(12.838-38.481) | 0.176(0.101-0.302) | 35.313(20.002-53.402) | 0.199(0.112-0.300) | 0.38(-0.06,0.83) |
| Türkiye | 207.376(109.780-334.421) | 0.361(0.191-0.582) | 356.626(253.493-458.743) | 0.415(0.295-0.534) | 0.56(0.23,0.89) |
| United Arab Emirates | 0.170(0.062-0.496) | 0.009(0.003-0.026) | 1.553(0.862-2.545) | 0.015(0.009-0.025) | 1.26(0.54,1.99) |
| Afghanistan | 14.555(7.320-27.287) | 0.142(0.071-0.266) | 34.282(19.651-53.371) | 0.095(0.054-0.147) | -0.86(-1.26,-0.47) |
| Bangladesh | 436.617(261.666-706.988) | 0.400(0.240-0.648) | 732.131(313.747-1208.136) | 0.421(0.180-0.695) | 0.22(0.04,0.40) |
| Bhutan | 1.917(0.918-3.639) | 0.310(0.148-0.589) | 3.450(1.549-6.455) | 0.462(0.207-0.865) | 0.91(0.74,1.07) |
| India | 2129.491(1254.108-3496.273) | 0.252(0.148-0.414) | 3612.218(2234.515-5623.327) | 0.250(0.155-0.390) | -0.06(-0.20,0.09) |
| Nepal | 73.003(38.405-125.464) | 0.375(0.197-0.645) | 116.527(66.606-192.549) | 0.375(0.214-0.620) | -0.19(-0.50,0.11) |
| Pakistan | 384.721(185.048-692.333) | 0.345(0.166-0.622) | 812.980(438.468-1370.551) | 0.333(0.180-0.562) | -0.48(-0.65,-0.32) |

**S5 Table: Incidence of UC in 1990 and 2023, and estimated annual percentage changes from 1990 to 2023.**

| Countries/  Regions | Number of cases, 1990 | Age-standardised rate per 100 000 population, 1990 | Number of cases, 2023 | Age-standardised rate per 100 000 population, 2023 | Estimated annual percentage change, 1990–2023 |
| --- | --- | --- | --- | --- | --- |
| East Asia | 8753.954(7056.917-11192.546) | 0.639(0.515-0.817) | 22096.585(17384.801-28944.760) | 1.350(1.062-1.768) | 3.08(2.53,3.63) |
| Southeast Asia | 1472.114(1180.492-1899.776) | 0.326(0.262-0.421) | 3367.153(2659.590-4406.466) | 0.485(0.383-0.634) | 1.23(1.19,1.26) |
| South Asia | 29177.761(23218.015-37653.017) | 2.621(2.085-3.382) | 78794.848(61956.374-102999.006) | 4.036(3.173-5.275) | 1.39(1.33,1.45) |
| Central Asia | 1170.113(934.252-1495.930) | 2.278(1.819-2.913) | 2732.221(2127.107-3524.589) | 3.446(2.683-4.445) | 1.23(1.15,1.31) |
| West Asia | 3625.151(2917.811-4643.306) | 1.815(1.461-2.325) | 9405.704(7440.425-12258.721) | 2.752(2.175-3.588) | 1.35(1.26,1.44) |
| China | 5824.924(4719.521-7463.589) | 0.494(0.400-0.633) | 17354.967(13607.351-22840.854) | 1.213(0.951-1.597) | 3.72(3.09,4.37) |
| Democratic People's Republic of Korea | 107.311(86.501-137.847) | 0.514(0.415-0.661) | 227.585(179.082-299.690) | 0.862(0.678-1.135) | 1.33(1.21,1.45) |
| Cambodia | 27.616(21.773-35.623) | 0.268(0.211-0.346) | 74.281(59.001-98.795) | 0.432(0.343-0.575) | 1.67(1.61,1.73) |
| Indonesia | 696.535(562.105-899.363) | 0.375(0.303-0.484) | 1557.382(1220.444-2038.754) | 0.542(0.425-0.709) | 1.01(0.95,1.08) |
| Lao People's Democratic Republic | 11.437(8.973-14.699) | 0.274(0.215-0.352) | 31.650(24.753-41.627) | 0.421(0.329-0.553) | 1.51(1.43,1.59) |
| Malaysia | 57.637(48.092-71.974) | 0.326(0.272-0.407) | 194.731(155.229-252.869) | 0.570(0.454-0.740) | 2.35(2.07,2.64) |
| Maldives | 0.540(0.431-0.674) | 0.243(0.194-0.303) | 2.618(2.078-3.413) | 0.482(0.383-0.629) | 2.47(2.35,2.60) |
| Myanmar | 130.329(102.884-166.138) | 0.322(0.254-0.410) | 265.888(213.457-342.128) | 0.462(0.371-0.595) | 1.19(1.16,1.22) |
| Philippines | 173.601(139.923-227.685) | 0.274(0.221-0.360) | 416.878(334.429-538.933) | 0.364(0.292-0.471) | 1.05(0.97,1.12) |
| Sri Lanka | 78.620(61.834-102.290) | 0.460(0.362-0.599) | 162.222(127.283-213.524) | 0.716(0.562-0.943) | 1.61(1.31,1.92) |
| Thailand | 166.467(132.641-216.905) | 0.292(0.232-0.380) | 299.185(230.179-390.304) | 0.455(0.350-0.594) | 1.23(1.12,1.35) |
| Timor-Leste | 2.110(1.653-2.743) | 0.270(0.212-0.351) | 5.143(4.175-6.628) | 0.358(0.291-0.462) | 0.86(0.84,0.87) |
| Viet Nam | 195.612(153.788-250.600) | 0.285(0.224-0.366) | 491.911(394.492-656.838) | 0.477(0.382-0.637) | 1.62(1.51,1.73) |
| Armenia | 92.306(74.438-121.020) | 2.690(2.169-3.526) | 134.882(105.434-176.701) | 4.449(3.478-5.829) | 1.57(1.50,1.63) |
| Azerbaijan | 216.261(168.451-281.890) | 2.939(2.289-3.830) | 459.645(351.393-610.097) | 4.368(3.340-5.798) | 1.35(1.28,1.42) |
| Georgia | 198.332(156.405-257.526) | 3.578(2.822-4.646) | 156.146(120.945-206.989) | 4.360(3.377-5.780) | 0.59(0.53,0.65) |
| Kazakhstan | 451.515(356.264-582.967) | 2.664(2.102-3.440) | 779.344(610.935-999.654) | 3.820(2.994-4.900) | 0.66(0.46,0.86) |
| Kyrgyzstan | 111.578(89.201-144.746) | 2.492(1.992-3.233) | 211.227(164.021-278.567) | 2.913(2.262-3.842) | 0.36(0.31,0.42) |
| Mongolia | 44.857(36.022-59.256) | 2.073(1.665-2.739) | 126.524(99.046-165.168) | 3.654(2.861-4.771) | 1.92(1.79,2.06) |
| Tajikistan | 111.043(90.058-141.929) | 2.066(1.676-2.641) | 290.248(231.187-375.340) | 2.716(2.163-3.512) | 0.97(0.89,1.05) |
| Turkmenistan | 91.834(75.107-117.239) | 2.473(2.022-3.157) | 203.332(158.125-261.734) | 3.625(2.819-4.666) | 1.28(1.23,1.33) |
| Uzbekistan | 404.142(323.621-509.049) | 1.938(1.552-2.442) | 1248.070(962.838-1609.294) | 3.531(2.724-4.553) | 2.10(1.97,2.23) |
| Brunei Darussalam | 1.253(1.014-1.613) | 0.480(0.389-0.618) | 2.621(2.106-3.387) | 0.570(0.458-0.736) | 0.49(0.35,0.64) |
| Japan | 1985.741(1569.784-2556.922) | 1.605(1.269-2.067) | 2230.039(1784.048-2843.112) | 1.789(1.431-2.280) | 0.27(-0.06,0.59) |
| Republic of Korea | 791.122(645.089-974.932) | 1.775(1.448-2.188) | 2157.470(1715.273-2795.936) | 4.173(3.318-5.408) | 3.21(2.52,3.90) |
| Singapore | 9.517(7.644-12.434) | 0.312(0.251-0.408) | 27.483(21.325-36.204) | 0.483(0.375-0.636) | 1.41(1.24,1.58) |
| Cyprus | 28.365(22.223-36.452) | 3.654(2.863-4.696) | 81.393(64.099-106.785) | 5.950(4.685-7.806) | 1.32(0.88,1.77) |
| Israel | 339.811(275.992-432.517) | 6.811(5.532-8.669) | 855.387(689.329-1098.774) | 8.563(6.900-10.999) | 0.09(-0.38,0.55) |
| Bahrain | 5.898(4.794-7.201) | 1.158(0.941-1.413) | 26.083(20.408-33.698) | 1.665(1.303-2.152) | 1.20(0.86,1.55) |
| Iran (Islamic Republic of) | 1019.810(826.363-1309.115) | 1.786(1.447-2.292) | 2551.954(2024.529-3316.226) | 2.902(2.303-3.772) | 1.97(1.70,2.24) |
| Iraq | 165.524(139.066-211.681) | 0.892(0.750-1.141) | 738.861(585.695-978.072) | 1.672(1.326-2.214) | 1.76(1.71,1.81) |
| Jordan | 58.749(48.847-68.921) | 1.563(1.299-1.833) | 332.168(265.937-424.465) | 2.516(2.015-3.216) | 1.43(1.33,1.52) |
| Kuwait | 40.808(36.183-46.279) | 2.397(2.126-2.719) | 126.589(97.691-167.504) | 2.705(2.088-3.579) | 0.19(-0.34,0.71) |
| Lebanon | 141.461(113.207-188.546) | 4.450(3.561-5.931) | 329.934(257.102-417.973) | 5.784(4.507-7.327) | 1.13(0.95,1.31) |
| Palestine | 24.228(19.616-30.560) | 1.177(0.953-1.484) | 105.666(83.562-135.061) | 1.954(1.545-2.497) | 1.63(1.53,1.73) |
| Oman | 25.621(20.007-32.714) | 1.283(1.002-1.638) | 139.521(107.357-184.183) | 2.717(2.091-3.587) | 2.47(2.36,2.58) |
| Qatar | 8.728(6.717-11.381) | 1.943(1.495-2.533) | 101.712(75.770-134.540) | 3.278(2.442-4.336) | 1.70(1.60,1.81) |
| Saudi Arabia | 188.662(151.618-240.888) | 1.161(0.933-1.482) | 475.873(371.467-609.998) | 1.443(1.126-1.849) | 0.74(0.64,0.85) |
| Syrian Arab Republic | 161.030(126.450-205.773) | 1.264(0.993-1.616) | 408.524(327.671-535.603) | 2.297(1.843-3.012) | 1.89(1.66,2.11) |
| Türkiye | 870.270(696.693-1109.545) | 1.515(1.212-1.931) | 1986.578(1607.947-2574.149) | 2.314(1.873-2.998) | 1.42(1.12,1.72) |
| United Arab Emirates | 39.286(30.740-51.296) | 2.087(1.633-2.725) | 394.787(284.088-547.904) | 3.897(2.804-5.408) | 1.81(1.76,1.86) |
| Afghanistan | 124.453(99.684-157.405) | 1.213(0.971-1.534) | 508.001(404.274-643.037) | 1.402(1.115-1.774) | 0.68(0.48,0.88) |
| Bangladesh | 2482.869(1993.756-3185.518) | 2.276(1.828-2.920) | 6351.821(4982.110-8284.058) | 3.653(2.866-4.765) | 1.53(1.47,1.60) |
| Bhutan | 14.390(11.325-18.765) | 2.328(1.832-3.035) | 34.084(27.142-44.145) | 4.567(3.637-5.915) | 2.08(1.94,2.21) |
| India | 23406.729(18626.676-30249.382) | 2.769(2.203-3.578) | 62391.874(49009.208-81570.217) | 4.323(3.395-5.651) | 1.45(1.38,1.53) |
| Nepal | 461.687(360.040-592.389) | 2.373(1.851-3.045) | 1202.697(950.015-1540.907) | 3.872(3.058-4.960) | 1.65(1.61,1.69) |
| Pakistan | 2608.474(2064.269-3346.594) | 2.342(1.854-3.005) | 8141.531(6454.264-10699.704) | 3.337(2.646-4.386) | 0.95(0.84,1.05) |

**S6 Table: DALYs of UC in 1990 and 2023, and estimated annual percentage changes from 1990 to 2023.**

| Countries/  Regions | Number of cases, 1990 | Age-standardised rate per 100 000 population, 1990 | Number of cases, 2023 | Age-standardised rate per 100 000 population, 2023 | Estimated annual percentage change, 1990–2023 |
| --- | --- | --- | --- | --- | --- |
| East Asia | 145862.737(81363.856-223971.977) | 10.644(5.937-16.343) | 130000.836(97872.520-173237.574) | 7.942(5.979-10.583) | -1.10(-1.29,-0.92) |
| Southeast Asia | 29744.374(15567.122-51878.337) | 6.591(3.449-11.495) | 45768.781(27012.776-71312.675) | 6.587(3.888-10.263) | -0.14(-0.27,-0.01) |
| South Asia | 123685.760(80708.498-182375.187) | 11.108(7.249-16.380) | 227363.566(156704.360-304600.355) | 11.645(8.026-15.601) | 0.01(-0.07,0.08) |
| Central Asia | 7758.910(6239.616-9710.522) | 15.106(12.148-18.906) | 9938.685(7757.095-12453.414) | 12.534(9.783-15.706) | -0.32(-0.56,-0.08) |
| West Asia | 19136.053(12317.188-28985.200) | 9.580(6.166-14.511) | 33922.791(24736.275-45106.264) | 9.838(7.180-13.084) | 0.02(-0.08,0.11) |
| China | 127149.006(68640.128-198948.196) | 10.783(5.821-16.872) | 106818.837(80680.972-142045.701) | 7.466(5.639-9.929) | -1.34(-1.54,-1.13) |
| Democratic People's Republic of Korea | 1800.865(1098.304-2713.641) | 8.630(5.263-13.004) | 2045.896(1083.921-3435.325) | 7.746(4.104-13.007) | -0.06(-0.17,0.05) |
| Cambodia | 577.582(234.579-1136.825) | 5.603(2.276-11.029) | 873.639(438.972-1492.327) | 5.082(2.553-8.681) | -0.34(-0.56,-0.12) |
| Indonesia | 16405.260(8858.004-28280.753) | 8.834(4.770-15.230) | 23513.937(13911.657-36298.170) | 8.180(4.839-12.627) | -0.51(-0.63,-0.39) |
| Lao People's Democratic Republic | 157.058(78.413-296.648) | 3.763(1.879-7.107) | 253.506(147.759-405.231) | 3.370(1.964-5.387) | -0.53(-0.79,-0.27) |
| Malaysia | 1590.454(702.400-2850.675) | 8.984(3.968-16.103) | 2954.205(1306.251-4920.191) | 8.647(3.823-14.401) | 0.48(-0.02,0.97) |
| Maldives | 8.790(4.994-15.484) | 3.954(2.247-6.966) | 22.744(15.273-34.409) | 4.192(2.815-6.341) | 0.33(0.10,0.55) |
| Myanmar | 3843.150(1599.201-7196.444) | 9.495(3.951-17.779) | 5006.281(2217.377-8972.519) | 8.705(3.856-15.601) | -0.34(-0.49,-0.19) |
| Philippines | 3108.170(1893.710-5155.733) | 4.915(2.994-8.152) | 5255.655(3859.316-7131.308) | 4.590(3.370-6.228) | -0.46(-0.72,-0.20) |
| Sri Lanka | 301.635(214.642-428.089) | 1.766(1.257-2.506) | 485.088(355.178-640.145) | 2.141(1.568-2.826) | 0.75(0.59,0.91) |
| Thailand | 1029.178(651.277-1578.309) | 1.803(1.141-2.765) | 2766.166(1968.677-3831.730) | 4.209(2.995-5.830) | 2.49(2.24,2.75) |
| Timor-Leste | 50.474(22.274-98.191) | 6.464(2.852-12.574) | 81.827(44.259-135.563) | 5.703(3.085-9.449) | -0.55(-0.85,-0.25) |
| Viet Nam | 2870.499(1438.574-5142.067) | 4.188(2.099-7.502) | 4865.440(2964.907-7872.550) | 4.717(2.874-7.632) | 0.56(0.45,0.68) |
| Armenia | 586.487(475.632-704.133) | 17.090(13.859-20.518) | 395.538(311.114-502.388) | 13.047(10.262-16.571) | -0.25(-0.46,-0.05) |
| Azerbaijan | 594.338(431.550-805.093) | 8.076(5.864-10.940) | 976.771(689.952-1346.366) | 9.283(6.557-12.796) | 0.52(0.37,0.66) |
| Georgia | 869.632(624.429-1162.610) | 15.689(11.265-20.974) | 415.703(314.233-558.216) | 11.609(8.775-15.588) | -0.61(-0.99,-0.23) |
| Kazakhstan | 2605.331(2121.370-3161.866) | 15.373(12.517-18.656) | 2908.348(2351.503-3512.119) | 14.255(11.526-17.214) | 0.26(-0.02,0.54) |
| Kyrgyzstan | 1021.214(812.558-1308.588) | 22.809(18.149-29.228) | 668.718(530.115-830.453) | 9.223(7.312-11.454) | -3.40(-4.05,-2.75) |
| Mongolia | 423.055(249.836-684.131) | 19.553(11.547-31.619) | 465.593(335.227-625.070) | 13.448(9.682-18.054) | -1.54(-1.72,-1.35) |
| Tajikistan | 656.220(435.425-946.481) | 12.212(8.103-17.613) | 1518.881(1024.065-2165.289) | 14.213(9.583-20.262) | 0.40(-0.01,0.81) |
| Turkmenistan | 454.308(355.159-573.972) | 12.233(9.563-15.455) | 791.756(600.417-1037.457) | 14.116(10.704-18.496) | 1.71(1.27,2.15) |
| Uzbekistan | 3021.837(2515.105-3719.616) | 14.494(12.063-17.841) | 4050.981(3250.995-4908.095) | 11.462(9.198-13.887) | -0.51(-0.90,-0.12) |
| Brunei Darussalam | 29.839(18.688-45.185) | 11.438(7.164-17.321) | 61.788(42.380-85.674) | 13.435(9.215-18.629) | 0.58(0.28,0.88) |
| Japan | 9348.733(7574.764-11549.248) | 7.558(6.124-9.337) | 13517.224(10777.178-16518.133) | 10.842(8.644-13.249) | 1.00(0.74,1.27) |
| Republic of Korea | 7141.078(3800.824-10076.760) | 16.024(8.529-22.612) | 7153.287(4995.222-10613.345) | 13.836(9.662-20.529) | -0.63(-0.79,-0.47) |
| Singapore | 82.709(70.003-97.507) | 2.714(2.297-3.200) | 136.338(111.220-167.410) | 2.395(1.954-2.941) | -1.35(-1.81,-0.89) |
| Cyprus | 209.130(134.954-317.406) | 26.940(17.384-40.888) | 361.530(265.022-466.533) | 26.427(19.373-34.102) | -0.20(-0.37,-0.02) |
| Israel | 738.380(519.516-1025.841) | 14.799(10.413-20.561) | 1860.187(1355.110-2540.626) | 18.621(13.565-25.432) | 0.34(0.05,0.63) |
| Bahrain | 25.601(15.970-43.473) | 5.024(3.134-8.532) | 103.746(72.795-139.838) | 6.624(4.648-8.929) | 0.57(0.43,0.71) |
| Iran (Islamic Republic of) | 5735.900(3692.386-8735.142) | 10.044(6.465-15.295) | 9700.626(7054.152-12951.229) | 11.033(8.023-14.730) | 0.16(0.00,0.32) |
| Iraq | 1407.024(829.740-2458.519) | 7.585(4.473-13.254) | 3578.114(2415.197-5093.066) | 8.099(5.467-11.529) | 0.22(0.07,0.36) |
| Jordan | 183.932(126.154-257.048) | 4.892(3.356-6.837) | 871.553(606.016-1212.226) | 6.603(4.591-9.183) | 1.12(0.98,1.25) |
| Kuwait | 136.309(102.575-166.879) | 8.008(6.026-9.804) | 310.513(218.028-446.634) | 6.635(4.659-9.544) | -0.80(-1.79,0.21) |
| Lebanon | 613.414(394.516-924.764) | 19.295(12.409-29.088) | 1149.827(824.935-1485.338) | 20.156(14.461-26.038) | -0.02(-0.13,0.09) |
| Palestine | 229.516(143.090-359.650) | 11.145(6.948-17.465) | 384.292(271.661-515.966) | 7.106(5.023-9.540) | -1.48(-1.82,-1.14) |
| Oman | 107.347(68.244-173.832) | 5.375(3.417-8.704) | 409.970(273.772-549.385) | 7.985(5.332-10.700) | 1.11(0.98,1.24) |
| Qatar | 33.733(21.591-55.652) | 7.508(4.806-12.387) | 249.882(174.182-349.548) | 8.053(5.614-11.266) | -0.26(-0.41,-0.11) |
| Saudi Arabia | 437.264(311.884-611.724) | 2.691(1.919-3.765) | 1314.032(901.500-1889.939) | 3.983(2.733-5.729) | 1.07(0.86,1.29) |
| Syrian Arab Republic | 993.306(646.719-1581.570) | 7.798(5.077-12.417) | 1488.189(1042.262-1921.030) | 8.369(5.861-10.804) | 0.12(-0.08,0.32) |
| Türkiye | 6176.373(3743.048-9508.783) | 10.749(6.514-16.548) | 9639.754(7490.899-12075.423) | 11.227(8.724-14.063) | 0.19(0.08,0.29) |
| United Arab Emirates | 58.363(35.192-93.081) | 3.100(1.869-4.944) | 712.564(455.444-1062.512) | 7.033(4.496-10.488) | 2.19(1.99,2.40) |
| Afghanistan | 524.933(305.577-917.467) | 5.115(2.978-8.941) | 1712.566(1120.090-2595.106) | 4.725(3.091-7.161) | -0.05(-0.36,0.26) |
| Bangladesh | 16731.381(11197.039-24182.959) | 15.338(10.265-22.169) | 29539.457(17615.255-41312.861) | 16.991(10.132-23.762) | 0.36(0.23,0.49) |
| Bhutan | 78.545(46.025-137.293) | 12.706(7.445-22.209) | 116.986(68.862-177.684) | 15.675(9.227-23.807) | 0.31(0.19,0.43) |
| India | 89152.475(59129.481-128381.921) | 10.546(6.995-15.187) | 161314.427(115625.815-208565.507) | 11.176(8.011-14.450) | 0.04(-0.06,0.13) |
| Nepal | 2915.676(1757.162-4836.173) | 14.988(9.033-24.860) | 4255.933(2812.371-5886.395) | 13.701(9.054-18.950) | -0.44(-0.74,-0.14) |
| Pakistan | 13972.325(8053.579-23475.802) | 12.547(7.232-21.081) | 29916.364(19091.517-45388.249) | 12.263(7.826-18.606) | -0.37(-0.47,-0.27) |

**S7 Table: Prevalence of UC in 1990 and 2023, and estimated annual percentage changes from 1990 to 2023.**

| Countries/  Regions | Number of cases, 1990 | Age-standardised rate per 100 000 population, 1990 | Number of cases, 2023 | Age-standardised rate per 100 000 population, 2023 | Estimated annual percentage change, 1990–2023 |
| --- | --- | --- | --- | --- | --- |
| East Asia | 83120.103(67090.807-103012.067) | 6.065(4.896-7.517) | 188693.154(153018.821-235875.713) | 11.528(9.348-14.410) | 2.61(2.03,3.19) |
| Southeast Asia | 12869.965(10177.043-16171.060) | 2.852(2.255-3.583) | 30282.165(24287.927-38090.006) | 4.358(3.496-5.482) | 1.28(1.24,1.33) |
| South Asia | 215260.671(171969.120-269920.414) | 19.333(15.445-24.242) | 634847.721(505951.148-794042.791) | 32.516(25.914-40.669) | 1.50(1.42,1.58) |
| Central Asia | 12039.629(9676.074-15119.989) | 23.441(18.839-29.438) | 25092.102(19787.559-32034.265) | 31.645(24.955-40.401) | 1.11(1.04,1.18) |
| West Asia | 35705.691(28444.832-44199.525) | 17.876(14.241-22.128) | 107117.796(85415.539-133536.508) | 31.321(24.971-39.045) | 1.60(1.48,1.73) |
| China | 42275.471(33887.065-52627.450) | 3.585(2.874-4.463) | 113607.448(91844.619-143175.823) | 7.941(6.420-10.008) | 3.43(2.73,4.13) |
| Democratic People's Republic of Korea | 1052.046(839.463-1317.706) | 5.042(4.023-6.315) | 1409.189(1107.535-1781.978) | 5.336(4.194-6.747) | 0.36(0.19,0.52) |
| Cambodia | 217.600(169.438-271.771) | 2.111(1.644-2.637) | 584.947(458.245-733.381) | 3.403(2.666-4.266) | 1.50(1.42,1.57) |
| Indonesia | 5453.932(4313.599-6971.748) | 2.937(2.323-3.754) | 12404.377(9916.345-15631.827) | 4.315(3.450-5.438) | 1.05(0.99,1.11) |
| Lao People's Democratic Republic | 90.290(68.509-113.049) | 2.163(1.641-2.708) | 251.877(195.358-317.418) | 3.349(2.597-4.220) | 1.47(1.34,1.61) |
| Malaysia | 614.878(507.026-737.463) | 3.473(2.864-4.166) | 2029.317(1636.960-2526.204) | 5.940(4.791-7.394) | 2.21(1.89,2.54) |
| Maldives | 5.237(4.082-6.438) | 2.356(1.836-2.896) | 29.497(23.464-36.421) | 5.436(4.324-6.712) | 2.63(2.59,2.67) |
| Myanmar | 1041.434(813.041-1303.421) | 2.573(2.009-3.220) | 2097.656(1681.222-2641.136) | 3.647(2.923-4.592) | 1.08(1.04,1.13) |
| Philippines | 1678.529(1309.250-2110.881) | 2.654(2.070-3.338) | 3749.734(3041.430-4757.152) | 3.275(2.656-4.154) | 0.57(0.50,0.64) |
| Sri Lanka | 764.994(594.529-961.160) | 4.479(3.481-5.627) | 1628.976(1290.684-2054.471) | 7.190(5.697-9.068) | 1.61(1.35,1.86) |
| Thailand | 1614.126(1283.002-2009.628) | 2.828(2.248-3.521) | 3366.190(2696.307-4241.201) | 5.122(4.102-6.453) | 1.72(1.65,1.80) |
| Timor-Leste | 17.796(13.765-22.527) | 2.279(1.763-2.885) | 41.203(32.753-51.750) | 2.872(2.283-3.607) | 0.55(0.42,0.69) |
| Viet Nam | 2010.369(1595.474-2468.007) | 2.933(2.328-3.601) | 5344.069(4304.813-6668.718) | 5.181(4.173-6.465) | 1.82(1.74,1.90) |
| Armenia | 1052.244(836.743-1325.926) | 30.661(24.382-38.636) | 1457.813(1165.946-1820.455) | 48.086(38.459-60.048) | 1.40(1.37,1.43) |
| Azerbaijan | 1859.747(1443.075-2349.818) | 25.270(19.609-31.930) | 4096.028(3206.261-5173.171) | 38.928(30.471-49.164) | 1.54(1.42,1.66) |
| Georgia | 2155.704(1749.622-2682.538) | 38.890(31.564-48.394) | 1750.799(1387.961-2179.992) | 48.891(38.759-60.876) | 0.78(0.71,0.86) |
| Kazakhstan | 5024.832(4014.154-6388.235) | 29.649(23.685-37.693) | 7358.241(5961.593-9286.531) | 36.066(29.220-45.517) | 0.93(0.81,1.04) |
| Kyrgyzstan | 990.254(794.782-1243.056) | 22.118(17.752-27.764) | 2184.508(1726.907-2796.262) | 30.130(23.818-38.567) | 1.00(0.91,1.08) |
| Mongolia | 378.909(302.952-469.078) | 17.512(14.002-21.680) | 980.562(781.817-1240.124) | 28.321(22.581-35.818) | 1.67(1.55,1.78) |
| Tajikistan | 963.302(743.443-1231.122) | 17.926(13.835-22.910) | 2518.818(1990.912-3175.502) | 23.571(18.631-29.716) | 1.06(0.95,1.18) |
| Turkmenistan | 716.194(582.589-891.568) | 19.285(15.687-24.007) | 1668.977(1316.281-2069.961) | 29.755(23.467-36.904) | 1.44(1.38,1.50) |
| Uzbekistan | 4345.047(3541.106-5366.007) | 20.840(16.984-25.737) | 11361.557(8791.867-14706.008) | 32.146(24.875-41.609) | 1.40(1.35,1.45) |
| Brunei Darussalam | 13.815(10.965-17.141) | 5.296(4.203-6.571) | 38.441(30.591-49.473) | 8.359(6.652-10.758) | 1.35(1.25,1.45) |
| Japan | 30727.374(25089.417-38076.685) | 24.843(20.285-30.785) | 44296.756(36490.916-54748.480) | 35.530(29.269-43.914) | 1.01(0.53,1.49) |
| Republic of Korea | 8686.305(6971.910-10521.148) | 19.492(15.645-23.609) | 28399.199(22793.934-34929.308) | 54.932(44.090-67.563) | 3.72(2.88,4.57) |
| Singapore | 117.195(92.974-145.424) | 3.846(3.051-4.773) | 374.356(293.903-471.744) | 6.577(5.164-8.288) | 1.66(1.42,1.90) |
| Cyprus | 413.537(324.178-528.227) | 53.271(41.760-68.045) | 961.464(772.578-1181.869) | 70.281(56.474-86.392) | 0.58(0.25,0.91) |
| Israel | 4219.845(3338.482-5226.209) | 84.578(66.913-104.749) | 9477.436(7494.692-11692.057) | 94.872(75.024-117.041) | -0.30(-0.68,0.07) |
| Bahrain | 63.056(49.742-80.580) | 12.375(9.762-15.815) | 370.905(287.027-475.983) | 23.683(18.327-30.392) | 2.16(1.90,2.41) |
| Iran (Islamic Republic of) | 10228.747(8207.627-12525.151) | 17.911(14.372-21.932) | 29965.344(24371.130-37356.801) | 34.080(27.718-42.487) | 2.17(1.95,2.40) |
| Iraq | 1398.820(1127.772-1720.776) | 7.541(6.080-9.277) | 7010.590(5394.049-8763.904) | 15.869(12.210-19.838) | 1.64(1.47,1.82) |
| Jordan | 640.207(532.348-748.784) | 17.029(14.160-19.917) | 4138.699(3268.446-5193.857) | 31.354(24.761-39.347) | 2.00(1.86,2.14) |
| Kuwait | 459.516(399.096-515.585) | 26.997(23.447-30.291) | 1594.153(1276.477-2023.654) | 34.065(27.277-43.243) | 0.19(-0.43,0.82) |
| Lebanon | 1481.534(1159.634-1909.420) | 46.601(36.476-60.060) | 4508.843(3600.560-5633.810) | 79.040(63.118-98.761) | 1.17(0.98,1.37) |
| Palestine | 218.879(169.401-270.652) | 10.629(8.226-13.143) | 1064.266(829.897-1344.642) | 19.679(15.345-24.863) | 1.49(1.38,1.61) |
| Oman | 228.784(178.115-289.711) | 11.456(8.919-14.507) | 1412.496(1094.519-1789.729) | 27.511(21.318-34.859) | 2.29(2.17,2.40) |
| Qatar | 93.267(73.860-120.919) | 20.759(16.439-26.913) | 1169.474(935.079-1469.675) | 37.691(30.137-47.366) | 1.40(1.25,1.55) |
| Saudi Arabia | 1526.404(1191.226-1892.112) | 9.394(7.331-11.645) | 4869.220(3889.727-6082.911) | 14.760(11.791-18.440) | 1.26(1.18,1.34) |
| Syrian Arab Republic | 1347.522(1047.857-1695.346) | 10.579(8.227-13.310) | 4226.208(3310.032-5288.529) | 23.767(18.615-29.742) | 2.16(2.06,2.26) |
| Türkiye | 7973.487(6339.691-9878.382) | 13.876(11.033-17.192) | 24371.351(19598.076-30078.796) | 28.383(22.824-35.030) | 2.29(1.87,2.71) |
| United Arab Emirates | 344.391(276.363-439.387) | 18.293(14.680-23.339) | 4672.707(3533.081-5986.673) | 46.123(34.874-59.092) | 2.40(2.19,2.61) |
| Afghanistan | 940.570(749.131-1182.015) | 9.166(7.300-11.519) | 3932.674(2984.647-5133.711) | 10.851(8.235-14.165) | 0.27(0.01,0.53) |
| Bangladesh | 19596.429(15518.943-24273.936) | 17.965(14.227-22.253) | 63086.703(48580.378-79684.434) | 36.286(27.943-45.833) | 2.33(2.21,2.44) |
| Bhutan | 109.134(87.578-136.194) | 17.654(14.167-22.031) | 264.916(205.646-335.031) | 35.495(27.554-44.890) | 2.05(1.94,2.16) |
| India | 170585.564(136648.068-214187.366) | 20.179(16.165-25.337) | 503002.993(403572.803-628197.826) | 34.849(27.960-43.523) | 1.54(1.45,1.64) |
| Nepal | 3673.650(2897.994-4709.554) | 18.884(14.897-24.210) | 10145.842(8066.405-12870.147) | 32.662(25.967-41.432) | 1.64(1.48,1.79) |
| Pakistan | 19585.094(15468.794-24463.752) | 17.587(13.891-21.968) | 52756.120(41227.122-65730.751) | 21.626(16.900-26.944) | 0.59(0.46,0.72) |

**S8 Table: Deaths of UC in 1990 and 2023, and estimated annual percentage changes from 1990 to 2023.**

| Countries/  Regions | Number of cases, 1990 | Age-standardised rate per 100 000 population, 1990 | Number of cases, 2023 | Age-standardised rate per 100 000 population, 2023 | Estimated annual percentage change, 1990–2023 |
| --- | --- | --- | --- | --- | --- |
| East Asia | 4177.313(2344.311-6455.103) | 0.305(0.171-0.471) | 5464.602(3922.503-7565.386) | 0.334(0.240-0.462) | -0.22(-0.51,0.08) |
| Southeast Asia | 801.452(408.330-1427.170) | 0.178(0.090-0.316) | 1453.048(812.096-2311.160) | 0.209(0.117-0.333) | 0.33(0.20,0.46) |
| South Asia | 2604.931(1477.676-4253.126) | 0.234(0.133-0.382) | 4550.212(2613.858-7274.766) | 0.233(0.134-0.373) | -0.09(-0.21,0.03) |
| Central Asia | 131.320(102.107-171.310) | 0.256(0.199-0.334) | 155.295(118.955-199.039) | 0.196(0.150-0.251) | -0.61(-0.94,-0.28) |
| West Asia | 392.774(225.272-648.113) | 0.197(0.113-0.324) | 727.695(450.110-1075.180) | 0.209(0.130-0.309) | 0.21(0.00,0.42) |
| China | 3633.094(2006.193-5681.806) | 0.308(0.170-0.482) | 4680.121(3365.247-6411.038) | 0.327(0.235-0.448) | -0.30(-0.61,0.01) |
| Democratic People's Republic of Korea | 48.594(26.967-81.510) | 0.233(0.129-0.391) | 76.811(36.339-134.823) | 0.291(0.138-0.510) | 1.03(0.87,1.19) |
| Cambodia | 11.777(4.621-23.789) | 0.114(0.045-0.231) | 27.070(12.028-48.889) | 0.157(0.070-0.284) | 1.11(0.92,1.30) |
| Indonesia | 470.239(251.017-831.595) | 0.253(0.135-0.448) | 767.142(439.377-1200.325) | 0.267(0.153-0.418) | -0.17(-0.30,-0.04) |
| Lao People's Democratic Republic | 3.810(1.888-7.180) | 0.091(0.045-0.172) | 7.010(3.778-11.725) | 0.093(0.050-0.156) | -0.00(-0.27,0.27) |
| Malaysia | 50.453(20.182-90.123) | 0.285(0.114-0.509) | 111.624(39.256-197.062) | 0.327(0.115-0.577) | 1.00(0.43,1.57) |
| Maldives | 0.195(0.102-0.348) | 0.088(0.046-0.157) | 0.710(0.407-1.184) | 0.131(0.075-0.218) | 1.54(1.38,1.70) |
| Myanmar | 81.861(34.417-154.047) | 0.202(0.085-0.381) | 113.702(51.250-201.117) | 0.198(0.089-0.350) | -0.21(-0.35,-0.07) |
| Philippines | 70.119(41.000-119.517) | 0.111(0.065-0.189) | 141.237(101.187-195.429) | 0.123(0.088-0.171) | -0.02(-0.30,0.27) |
| Sri Lanka | 5.941(3.953-9.184) | 0.035(0.023-0.054) | 11.291(6.508-16.804) | 0.050(0.029-0.074) | 1.04(0.69,1.39) |
| Thailand | 23.884(12.410-41.027) | 0.042(0.022-0.072) | 102.457(65.466-149.932) | 0.156(0.100-0.228) | 4.10(3.74,4.45) |
| Timor-Leste | 0.986(0.436-1.945) | 0.126(0.056-0.249) | 2.514(1.276-4.382) | 0.175(0.089-0.305) | 0.96(0.78,1.15) |
| Viet Nam | 84.903(39.632-153.597) | 0.124(0.058-0.224) | 173.489(93.277-293.541) | 0.168(0.090-0.285) | 1.17(1.08,1.27) |
| Armenia | 12.148(9.807-15.013) | 0.354(0.286-0.437) | 7.200(5.929-8.699) | 0.238(0.196-0.287) | -0.41(-0.77,-0.05) |
| Azerbaijan | 7.445(4.669-11.869) | 0.101(0.063-0.161) | 9.377(5.503-14.664) | 0.089(0.052-0.139) | -0.45(-0.63,-0.27) |
| Georgia | 16.748(9.750-26.135) | 0.302(0.176-0.471) | 6.246(4.723-8.160) | 0.174(0.132-0.228) | -1.16(-2.06,-0.25) |
| Kazakhstan | 46.475(37.259-57.148) | 0.274(0.220-0.337) | 55.208(44.233-67.309) | 0.271(0.217-0.330) | 0.55(0.21,0.89) |
| Kyrgyzstan | 18.763(14.519-24.798) | 0.419(0.324-0.554) | 8.289(6.803-9.970) | 0.114(0.094-0.138) | -4.86(-5.71,-3.99) |
| Mongolia | 7.166(3.770-11.583) | 0.331(0.174-0.535) | 8.779(5.609-12.947) | 0.254(0.162-0.374) | -1.55(-1.85,-1.24) |
| Tajikistan | 12.365(7.340-20.159) | 0.230(0.137-0.375) | 25.890(15.601-40.884) | 0.242(0.146-0.383) | -0.27(-0.76,0.22) |
| Turkmenistan | 6.670(5.070-8.927) | 0.180(0.137-0.240) | 12.381(8.583-17.760) | 0.221(0.153-0.317) | 1.76(1.30,2.23) |
| Uzbekistan | 47.047(37.919-60.278) | 0.226(0.182-0.289) | 53.526(43.736-63.116) | 0.151(0.124-0.179) | -1.17(-1.82,-0.53) |
| Brunei Darussalam | 0.782(0.482-1.167) | 0.300(0.185-0.447) | 1.980(1.290-2.866) | 0.431(0.280-0.623) | 1.19(0.84,1.53) |
| Japan | 255.733(212.562-303.880) | 0.207(0.172-0.246) | 503.952(394.402-618.867) | 0.404(0.316-0.496) | 2.19(1.53,2.85) |
| Republic of Korea | 232.727(94.820-376.324) | 0.522(0.213-0.844) | 194.938(120.907-387.712) | 0.377(0.234-0.750) | -2.39(-2.81,-1.97) |
| Singapore | 2.639(2.246-3.183) | 0.087(0.074-0.104) | 4.823(3.912-5.892) | 0.085(0.069-0.104) | -2.18(-3.14,-1.21) |
| Cyprus | 9.522(5.311-16.838) | 1.227(0.684-2.169) | 15.995(10.429-24.463) | 1.169(0.762-1.788) | -0.18(-0.33,-0.03) |
| Israel | 6.656(5.196-8.425) | 0.133(0.104-0.169) | 36.751(28.969-44.323) | 0.368(0.290-0.444) | 3.56(2.86,4.26) |
| Bahrain | 0.427(0.194-0.909) | 0.084(0.038-0.178) | 1.495(0.881-2.361) | 0.095(0.056-0.151) | -0.34(-0.58,-0.11) |
| Iran (Islamic Republic of) | 108.070(65.024-171.423) | 0.189(0.114-0.300) | 207.592(105.693-346.656) | 0.236(0.120-0.394) | 0.32(0.05,0.59) |
| Iraq | 25.179(12.640-49.880) | 0.136(0.068-0.269) | 64.301(35.499-106.292) | 0.146(0.080-0.241) | 0.42(0.22,0.62) |
| Jordan | 2.486(1.229-4.699) | 0.066(0.033-0.125) | 9.138(5.001-14.313) | 0.069(0.038-0.108) | 0.52(0.24,0.81) |
| Kuwait | 1.612(1.150-2.122) | 0.095(0.068-0.125) | 2.704(2.113-3.429) | 0.058(0.045-0.073) | -1.16(-3.39,1.13) |
| Lebanon | 15.125(7.273-26.424) | 0.476(0.229-0.831) | 23.101(14.915-32.365) | 0.405(0.261-0.567) | -0.42(-0.69,-0.14) |
| Palestine | 7.118(3.964-11.898) | 0.346(0.192-0.578) | 8.261(5.198-12.017) | 0.153(0.096-0.222) | -2.64(-3.08,-2.20) |
| Oman | 1.764(0.890-3.280) | 0.088(0.045-0.164) | 5.887(3.322-9.371) | 0.115(0.065-0.183) | 0.94(0.66,1.23) |
| Qatar | 0.513(0.233-0.971) | 0.114(0.052-0.216) | 2.025(1.233-3.062) | 0.065(0.040-0.099) | -2.35(-2.65,-2.05) |
| Saudi Arabia | 4.471(2.377-7.649) | 0.028(0.015-0.047) | 15.875(6.673-27.676) | 0.048(0.020-0.084) | 1.52(1.13,1.91) |
| Syrian Arab Republic | 19.712(11.577-33.560) | 0.155(0.091-0.263) | 31.903(17.800-47.104) | 0.179(0.100-0.265) | 0.47(0.03,0.92) |
| Türkiye | 153.662(83.944-256.693) | 0.267(0.146-0.447) | 278.768(195.691-368.395) | 0.325(0.228-0.429) | 0.77(0.42,1.12) |
| United Arab Emirates | 0.117(0.044-0.324) | 0.006(0.002-0.017) | 1.075(0.540-1.830) | 0.011(0.005-0.018) | 1.11(0.36,1.86) |
| Afghanistan | 11.763(5.551-24.118) | 0.115(0.054-0.235) | 27.787(15.528-44.808) | 0.077(0.043-0.124) | -0.79(-1.19,-0.39) |
| Bangladesh | 381.926(233.421-594.572) | 0.350(0.214-0.545) | 639.895(263.304-1030.921) | 0.368(0.151-0.593) | 0.19(0.01,0.38) |
| Bhutan | 1.655(0.818-3.099) | 0.268(0.132-0.501) | 2.966(1.385-5.441) | 0.397(0.186-0.729) | 0.88(0.70,1.05) |
| India | 1805.074(1037.659-2895.425) | 0.214(0.123-0.343) | 3071.985(1892.646-4828.110) | 0.213(0.131-0.334) | -0.05(-0.20,0.11) |
| Nepal | 62.811(32.577-111.268) | 0.323(0.167-0.572) | 98.304(55.551-157.571) | 0.316(0.179-0.507) | -0.28(-0.59,0.02) |
| Pakistan | 335.566(163.597-615.111) | 0.301(0.147-0.552) | 697.274(378.529-1189.927) | 0.286(0.155-0.488) | -0.54(-0.71,-0.37) |

**S9 Table: Incidence of CD in 1990 and 2023, and estimated annual percentage changes from 1990 to 2023.**

| Countries/  Regions | Number of cases, 1990 | Age-standardised rate per 100 000 population, 1990 | Number of cases, 2023 | Age-standardised rate per 100 000 population, 2023 | Estimated annual percentage change, 1990–2023 |
| --- | --- | --- | --- | --- | --- |
| East Asia | 4217.366(3428.884-5471.255) | 0.308(0.250-0.399) | 10538.750(8671.189-13457.336) | 0.644(0.530-0.822) | 2.29(1.82,2.76) |
| Southeast Asia | 675.245(531.064-901.410) | 0.150(0.118-0.200) | 1393.805(1123.529-1805.901) | 0.201(0.162-0.260) | 0.91(0.84,0.98) |
| South Asia | 10628.553(8765.792-13414.084) | 0.955(0.787-1.205) | 28001.066(23339.516-34848.923) | 1.434(1.195-1.785) | 1.47(1.35,1.58) |
| Central Asia | 520.368(420.809-660.419) | 1.013(0.819-1.286) | 1131.238(923.676-1442.291) | 1.427(1.165-1.819) | 1.04(0.95,1.14) |
| West Asia | 1338.975(1086.566-1698.110) | 0.670(0.544-0.850) | 3536.176(2896.528-4482.092) | 1.034(0.847-1.311) | 1.23(1.05,1.41) |
| China | 2626.787(2129.452-3417.639) | 0.223(0.181-0.290) | 8127.479(6680.664-10433.296) | 0.568(0.467-0.729) | 2.99(2.38,3.60) |
| Democratic People's Republic of Korea | 38.611(30.785-51.382) | 0.185(0.148-0.246) | 71.343(58.471-91.967) | 0.270(0.221-0.348) | 1.00(0.88,1.13) |
| Cambodia | 13.248(10.335-17.771) | 0.129(0.100-0.172) | 32.387(25.605-43.555) | 0.188(0.149-0.253) | 1.35(1.27,1.42) |
| Indonesia | 302.302(240.965-404.019) | 0.163(0.130-0.218) | 617.561(501.846-797.456) | 0.215(0.175-0.277) | 0.80(0.75,0.85) |
| Lao People's Democratic Republic | 5.345(4.130-7.040) | 0.128(0.099-0.169) | 13.960(10.748-18.140) | 0.186(0.143-0.241) | 1.29(1.24,1.34) |
| Malaysia | 31.299(24.731-41.764) | 0.177(0.140-0.236) | 95.233(75.524-126.633) | 0.279(0.221-0.371) | 1.58(1.37,1.80) |
| Maldives | 0.258(0.198-0.338) | 0.116(0.089-0.152) | 1.071(0.845-1.417) | 0.197(0.156-0.261) | 1.93(1.76,2.10) |
| Myanmar | 59.738(46.446-78.360) | 0.148(0.115-0.194) | 114.346(91.688-146.951) | 0.199(0.159-0.256) | 0.93(0.88,0.98) |
| Philippines | 52.140(41.303-69.028) | 0.082(0.065-0.109) | 119.914(96.257-155.155) | 0.105(0.084-0.135) | 0.86(0.77,0.94) |
| Sri Lanka | 35.577(28.439-48.560) | 0.208(0.167-0.284) | 68.941(55.720-89.290) | 0.304(0.246-0.394) | 1.24(0.99,1.50) |
| Thailand | 110.833(86.628-149.469) | 0.194(0.152-0.262) | 178.732(144.225-227.279) | 0.272(0.219-0.346) | 1.01(0.89,1.13) |
| Timor-Leste | 0.993(0.771-1.356) | 0.127(0.099-0.174) | 2.368(1.865-3.088) | 0.165(0.130-0.215) | 0.81(0.77,0.84) |
| Viet Nam | 91.253(69.430-121.914) | 0.133(0.101-0.178) | 198.269(158.789-260.768) | 0.192(0.154-0.253) | 1.17(1.02,1.31) |
| Armenia | 38.109(30.687-48.377) | 1.110(0.894-1.410) | 50.563(41.649-63.026) | 1.668(1.374-2.079) | 1.37(1.29,1.44) |
| Azerbaijan | 94.504(77.200-120.733) | 1.284(1.049-1.641) | 181.336(148.759-228.939) | 1.723(1.414-2.176) | 1.07(1.01,1.14) |
| Georgia | 81.117(67.479-100.921) | 1.463(1.217-1.821) | 58.420(47.075-73.335) | 1.631(1.315-2.048) | 0.33(0.26,0.40) |
| Kazakhstan | 194.228(157.506-241.491) | 1.146(0.929-1.425) | 314.215(262.427-398.443) | 1.540(1.286-1.953) | 0.47(0.26,0.69) |
| Kyrgyzstan | 49.005(39.250-62.721) | 1.095(0.877-1.401) | 87.528(70.890-110.756) | 1.207(0.978-1.528) | 0.23(0.16,0.30) |
| Mongolia | 20.648(16.790-26.295) | 0.954(0.776-1.215) | 52.523(43.135-67.121) | 1.517(1.246-1.939) | 1.62(1.48,1.77) |
| Tajikistan | 50.505(40.519-65.065) | 0.940(0.754-1.211) | 124.411(100.960-159.458) | 1.164(0.945-1.492) | 0.76(0.70,0.83) |
| Turkmenistan | 42.244(33.799-54.933) | 1.138(0.910-1.479) | 85.160(69.285-108.829) | 1.518(1.235-1.940) | 1.05(1.01,1.10) |
| Uzbekistan | 184.386(149.735-236.209) | 0.884(0.718-1.133) | 519.923(420.113-664.805) | 1.471(1.189-1.881) | 1.87(1.71,2.03) |
| Brunei Darussalam | 0.724(0.566-0.983) | 0.277(0.217-0.377) | 1.460(1.174-1.930) | 0.317(0.255-0.420) | 0.54(0.46,0.61) |
| Japan | 711.893(593.394-892.190) | 0.576(0.480-0.721) | 871.055(708.237-1083.591) | 0.699(0.568-0.869) | -0.33(-0.67,0.01) |
| Republic of Korea | 819.428(658.461-1083.750) | 1.839(1.478-2.432) | 1416.350(1180.681-1781.361) | 2.740(2.284-3.446) | 1.42(1.10,1.74) |
| Singapore | 7.368(5.758-9.707) | 0.242(0.189-0.319) | 19.575(15.807-24.945) | 0.344(0.278-0.438) | 1.18(1.06,1.30) |
| Cyprus | 28.784(23.365-36.496) | 3.708(3.010-4.701) | 82.554(67.373-102.668) | 6.034(4.925-7.505) | 1.64(1.56,1.71) |
| Israel | 159.501(135.322-192.903) | 3.197(2.712-3.866) | 459.104(373.550-584.597) | 4.596(3.739-5.852) | 0.43(-0.17,1.04) |
| Bahrain | 4.508(3.510-5.803) | 0.885(0.689-1.139) | 17.298(14.134-22.028) | 1.104(0.903-1.406) | 0.76(0.42,1.10) |
| Iran (Islamic Republic of) | 139.905(114.745-183.040) | 0.245(0.201-0.321) | 339.187(281.452-431.982) | 0.386(0.320-0.491) | 1.80(1.61,1.99) |
| Iraq | 96.856(76.960-123.174) | 0.522(0.415-0.664) | 365.022(296.068-469.438) | 0.826(0.670-1.063) | 1.69(1.59,1.79) |
| Jordan | 18.006(14.345-23.036) | 0.479(0.382-0.613) | 101.490(83.018-129.846) | 0.769(0.629-0.984) | 1.31(1.19,1.43) |
| Kuwait | 11.155(8.717-14.748) | 0.655(0.512-0.866) | 39.155(31.025-50.401) | 0.837(0.663-1.077) | 1.41(1.23,1.60) |
| Lebanon | 46.466(37.377-58.225) | 1.462(1.176-1.831) | 104.999(84.927-134.416) | 1.841(1.489-2.356) | 1.04(0.93,1.15) |
| Palestine | 9.898(7.934-13.061) | 0.481(0.385-0.634) | 40.249(32.039-51.584) | 0.744(0.592-0.954) | 1.69(1.57,1.82) |
| Oman | 10.694(8.677-14.142) | 0.536(0.434-0.708) | 51.907(40.793-66.593) | 1.011(0.795-1.297) | 2.44(2.27,2.62) |
| Qatar | 3.125(2.453-4.030) | 0.695(0.546-0.897) | 35.353(27.114-45.637) | 1.139(0.874-1.471) | 2.04(1.86,2.23) |
| Saudi Arabia | 97.066(76.966-126.712) | 0.597(0.474-0.780) | 397.858(322.457-502.425) | 1.206(0.977-1.523) | 1.70(1.51,1.89) |
| Syrian Arab Republic | 65.456(52.122-84.609) | 0.514(0.409-0.664) | 145.599(119.253-180.886) | 0.819(0.671-1.017) | 1.68(1.50,1.86) |
| Türkiye | 419.212(337.673-529.211) | 0.730(0.588-0.921) | 938.914(786.599-1181.698) | 1.093(0.916-1.376) | 1.07(0.72,1.41) |
| United Arab Emirates | 14.612(11.036-18.888) | 0.776(0.586-1.003) | 127.168(99.241-162.594) | 1.255(0.980-1.605) | 1.73(1.60,1.87) |
| Afghanistan | 45.945(37.560-57.676) | 0.448(0.366-0.562) | 195.215(156.231-253.668) | 0.539(0.431-0.700) | 0.93(0.76,1.11) |
| Bangladesh | 918.338(746.796-1180.683) | 0.842(0.685-1.082) | 2248.062(1845.822-2802.570) | 1.293(1.062-1.612) | 1.43(1.38,1.49) |
| Bhutan | 5.275(4.299-6.782) | 0.853(0.695-1.097) | 11.908(9.889-14.928) | 1.596(1.325-2.000) | 1.94(1.81,2.08) |
| India | 8520.775(7036.818-10741.834) | 1.008(0.832-1.271) | 22083.370(18441.838-27400.025) | 1.530(1.278-1.898) | 1.55(1.41,1.70) |
| Nepal | 168.909(136.061-215.490) | 0.868(0.699-1.108) | 423.493(352.680-539.049) | 1.363(1.135-1.735) | 1.55(1.51,1.59) |
| Pakistan | 933.475(775.620-1162.721) | 0.838(0.697-1.044) | 2969.005(2476.491-3747.976) | 1.217(1.015-1.536) | 0.98(0.88,1.07) |

**S10 Table: DALYs of CD in 1990 and 2023, and estimated annual percentage changes from 1990 to 2023.**

| Countries/  Regions | Number of cases, 1990 | Age-standardised rate per 100 000 population, 1990 | Number of cases, 2023 | Age-standardised rate per 100 000 population, 2023 | Estimated annual percentage change, 1990–2023 |
| --- | --- | --- | --- | --- | --- |
| East Asia | 17277.794(11504.658-25604.604) | 1.261(0.839-1.868) | 23760.059(17066.523-31377.865) | 1.452(1.043-1.917) | 0.40(0.11,0.69) |
| Southeast Asia | 3710.329(2147.009-6680.983) | 0.822(0.476-1.480) | 7928.675(4252.145-14221.627) | 1.141(0.612-2.047) | 1.06(1.00,1.13) |
| South Asia | 33824.744(21845.730-53009.731) | 3.038(1.962-4.761) | 70914.043(45763.805-101869.237) | 3.632(2.344-5.218) | 0.50(0.40,0.59) |
| Central Asia | 2536.297(1859.701-3483.101) | 4.938(3.621-6.782) | 3801.687(2813.795-4912.170) | 4.795(3.549-6.195) | 0.01(-0.20,0.22) |
| West Asia | 8564.777(5409.212-13471.373) | 4.288(2.708-6.744) | 14942.507(10147.019-21300.591) | 4.377(2.984-6.217) | -0.07(-0.15,0.01) |
| China | 11043.942(6928.587-17398.032) | 0.937(0.588-1.475) | 16315.524(11623.231-21397.409) | 1.140(0.812-1.496) | 0.58(0.18,0.97) |
| Democratic People's Republic of Korea | 254.416(139.444-468.605) | 1.219(0.668-2.246) | 255.493(140.450-444.842) | 0.967(0.532-1.684) | -0.41(-0.61,-0.20) |
| Cambodia | 69.248(34.031-139.737) | 0.672(0.330-1.356) | 132.139(71.603-238.971) | 0.769(0.417-1.390) | 0.43(0.28,0.59) |
| Indonesia | 1867.605(1014.435-3608.189) | 1.006(0.546-1.943) | 3795.610(1846.964-7411.378) | 1.320(0.642-2.578) | 0.77(0.67,0.86) |
| Lao People's Democratic Republic | 18.784(11.030-32.964) | 0.450(0.264-0.790) | 51.003(27.885-86.244) | 0.678(0.371-1.147) | 1.49(1.35,1.63) |
| Malaysia | 208.668(123.373-363.895) | 1.179(0.697-2.056) | 500.214(311.952-819.194) | 1.464(0.913-2.398) | 1.13(0.85,1.41) |
| Maldives | 2.470(1.326-4.813) | 1.111(0.596-2.165) | 9.034(5.518-14.632) | 1.665(1.017-2.697) | 1.56(1.39,1.73) |
| Myanmar | 456.716(239.944-859.046) | 1.128(0.593-2.122) | 920.677(407.998-1790.596) | 1.601(0.709-3.113) | 1.23(1.11,1.35) |
| Philippines | 317.152(207.515-514.082) | 0.501(0.328-0.813) | 826.873(534.834-1228.632) | 0.722(0.467-1.073) | 1.28(1.14,1.41) |
| Sri Lanka | 115.554(80.562-159.578) | 0.677(0.472-0.934) | 185.720(131.303-258.049) | 0.820(0.580-1.139) | 0.73(0.57,0.90) |
| Thailand | 293.099(204.356-413.598) | 0.514(0.358-0.725) | 539.115(378.107-745.242) | 0.820(0.575-1.134) | 1.27(1.16,1.37) |
| Timor-Leste | 5.325(2.907-9.461) | 0.682(0.372-1.212) | 11.034(6.020-18.177) | 0.769(0.420-1.267) | 0.34(0.02,0.65) |
| Viet Nam | 420.331(266.948-672.690) | 0.613(0.389-0.981) | 1076.285(609.766-1784.133) | 1.043(0.591-1.730) | 1.94(1.82,2.07) |
| Armenia | 165.869(118.963-225.861) | 4.833(3.466-6.581) | 136.707(96.984-182.870) | 4.509(3.199-6.032) | 0.38(0.13,0.63) |
| Azerbaijan | 250.850(175.748-371.714) | 3.409(2.388-5.051) | 473.519(299.881-687.119) | 4.500(2.850-6.530) | 1.19(0.97,1.41) |
| Georgia | 936.892(660.194-1398.939) | 16.902(11.910-25.238) | 258.027(193.986-328.944) | 7.205(5.417-9.186) | -2.99(-4.05,-1.93) |
| Kazakhstan | 1211.806(920.771-1631.179) | 7.150(5.433-9.625) | 1425.210(1087.498-1803.021) | 6.986(5.330-8.837) | -0.16(-0.43,0.11) |
| Kyrgyzstan | 381.640(271.735-545.710) | 8.524(6.069-12.189) | 252.956(176.347-345.561) | 3.489(2.432-4.766) | -3.32(-4.18,-2.45) |
| Mongolia | 57.027(36.484-86.738) | 2.636(1.686-4.009) | 129.955(88.893-184.563) | 3.753(2.567-5.331) | 1.35(1.24,1.47) |
| Tajikistan | 82.541(48.338-122.843) | 1.536(0.900-2.286) | 199.563(120.605-295.415) | 1.867(1.129-2.764) | 0.73(0.63,0.84) |
| Turkmenistan | 154.410(109.705-216.445) | 4.158(2.954-5.828) | 273.576(179.672-395.445) | 4.877(3.203-7.050) | 0.83(0.71,0.95) |
| Uzbekistan | 705.900(509.152-966.924) | 3.386(2.442-4.638) | 1650.383(1249.674-2072.729) | 4.670(3.536-5.865) | 1.50(1.08,1.91) |
| Brunei Darussalam | 2.274(1.500-3.610) | 0.872(0.575-1.384) | 4.748(3.230-6.884) | 1.033(0.702-1.497) | 0.64(0.52,0.77) |
| Japan | 3495.813(2768.699-4306.096) | 2.826(2.238-3.481) | 4102.887(3178.426-5324.807) | 3.291(2.549-4.271) | 0.10(-0.10,0.30) |
| Republic of Korea | 2426.597(1631.445-3345.134) | 5.445(3.661-7.506) | 2956.201(2035.523-4026.244) | 5.718(3.937-7.788) | 0.31(0.02,0.60) |
| Singapore | 51.129(40.969-63.710) | 1.678(1.345-2.091) | 70.977(53.785-92.175) | 1.247(0.945-1.619) | -0.79(-1.05,-0.53) |
| Cyprus | 122.757(82.168-183.067) | 15.813(10.585-23.582) | 234.689(165.461-325.563) | 17.155(12.095-23.798) | 0.18(0.13,0.22) |
| Israel | 481.462(372.557-627.613) | 9.650(7.467-12.579) | 1174.230(878.589-1538.309) | 11.754(8.795-15.399) | 0.10(-0.56,0.76) |
| Bahrain | 17.196(11.737-23.270) | 3.375(2.304-4.567) | 69.082(44.519-99.768) | 4.411(2.843-6.370) | 0.84(0.52,1.16) |
| Iran (Islamic Republic of) | 1887.521(1044.940-3300.259) | 3.305(1.830-5.779) | 2686.958(1766.252-4112.329) | 3.056(2.009-4.677) | -0.30(-0.49,-0.11) |
| Iraq | 510.560(324.504-820.925) | 2.752(1.749-4.426) | 1435.804(919.642-2177.860) | 3.250(2.082-4.930) | 0.37(0.31,0.42) |
| Jordan | 120.040(74.067-192.858) | 3.193(1.970-5.130) | 536.994(377.279-768.063) | 4.068(2.858-5.819) | 1.21(0.94,1.48) |
| Kuwait | 32.972(22.886-46.733) | 1.937(1.345-2.746) | 157.729(109.916-210.777) | 3.370(2.349-4.504) | 1.75(0.90,2.61) |
| Lebanon | 309.773(189.499-513.932) | 9.744(5.961-16.166) | 597.140(409.748-840.522) | 10.468(7.183-14.734) | 0.20(0.07,0.33) |
| Palestine | 136.833(80.505-230.638) | 6.645(3.909-11.200) | 316.303(216.508-462.298) | 5.849(4.003-8.548) | -0.49(-0.64,-0.34) |
| Oman | 44.085(27.680-75.932) | 2.208(1.386-3.802) | 200.409(119.247-312.386) | 3.903(2.323-6.084) | 1.88(1.72,2.04) |
| Qatar | 11.446(7.019-16.962) | 2.548(1.562-3.775) | 105.336(64.722-154.525) | 3.395(2.086-4.980) | 0.72(0.63,0.81) |
| Saudi Arabia | 256.488(175.051-352.396) | 1.578(1.077-2.169) | 1143.322(731.879-1681.889) | 3.466(2.219-5.098) | 2.02(1.78,2.27) |
| Syrian Arab Republic | 245.465(134.587-437.626) | 1.927(1.057-3.436) | 431.313(276.716-622.759) | 2.426(1.556-3.502) | 0.65(0.55,0.75) |
| Türkiye | 3004.403(1888.933-4608.644) | 5.229(3.287-8.021) | 4658.895(3281.355-6259.925) | 5.426(3.822-7.290) | 0.00(-0.05,0.06) |
| United Arab Emirates | 30.164(18.172-44.005) | 1.602(0.965-2.337) | 326.050(194.336-534.684) | 3.218(1.918-5.278) | 2.19(2.11,2.27) |
| Afghanistan | 179.889(102.462-314.548) | 1.753(0.998-3.065) | 645.268(382.187-939.783) | 1.780(1.055-2.593) | 0.10(-0.15,0.36) |
| Bangladesh | 3792.363(2169.655-6223.540) | 3.477(1.989-5.705) | 7875.420(5062.447-12255.365) | 4.530(2.912-7.049) | 0.97(0.82,1.12) |
| Bhutan | 19.657(11.322-36.697) | 3.180(1.831-5.936) | 34.201(20.141-52.415) | 4.582(2.699-7.023) | 0.93(0.87,0.99) |
| India | 25600.058(17076.437-39313.072) | 3.028(2.020-4.650) | 52527.486(34350.571-73213.223) | 3.639(2.380-5.072) | 0.51(0.40,0.62) |
| Nepal | 735.049(383.720-1280.000) | 3.779(1.973-6.580) | 1332.522(831.827-1939.744) | 4.290(2.678-6.244) | 0.33(0.06,0.60) |
| Pakistan | 3379.704(2020.245-5677.483) | 3.035(1.814-5.098) | 8304.391(4979.812-13196.026) | 3.404(2.041-5.409) | 0.09(0.02,0.16) |

**S11 Table: Prevalence of CD in 1990 and 2023, and estimated annual percentage changes from 1990 to 2023.**

| Countries/  Regions | Number of cases, 1990 | Age-standardised rate per 100 000 population, 1990 | Number of cases, 2023 | Age-standardised rate per 100 000 population, 2023 | Estimated annual percentage change, 1990–2023 |
| --- | --- | --- | --- | --- | --- |
| East Asia | 37677.776(29160.583-48291.582) | 2.749(2.128-3.524) | 89647.300(72054.061-111718.156) | 5.477(4.402-6.825) | 2.08(1.52,2.64) |
| Southeast Asia | 6381.512(4773.459-8424.508) | 1.414(1.058-1.867) | 13380.961(10252.250-17198.597) | 1.926(1.475-2.475) | 0.91(0.86,0.96) |
| South Asia | 86769.452(69045.502-109741.452) | 7.793(6.201-9.856) | 248970.209(198846.063-311390.914) | 12.752(10.184-15.949) | 1.55(1.41,1.68) |
| Central Asia | 5669.507(4428.002-7240.244) | 11.038(8.621-14.097) | 11155.730(8723.765-14118.024) | 14.069(11.002-17.805) | 0.94(0.87,1.02) |
| West Asia | 14916.667(11591.763-18953.839) | 7.468(5.803-9.489) | 46207.024(36238.666-58395.060) | 13.495(10.579-17.057) | 1.57(1.30,1.84) |
| China | 21574.569(16534.270-28106.068) | 1.830(1.402-2.384) | 62291.473(49900.131-77787.017) | 4.354(3.488-5.437) | 2.79(2.09,3.50) |
| Democratic People's Republic of Korea | 456.176(338.039-608.163) | 2.186(1.620-2.914) | 570.486(439.672-722.347) | 2.160(1.665-2.735) | 0.10(-0.05,0.26) |
| Cambodia | 115.045(81.791-155.162) | 1.116(0.793-1.505) | 276.820(204.861-360.488) | 1.610(1.192-2.097) | 1.13(1.08,1.19) |
| Indonesia | 2545.056(1916.263-3344.382) | 1.371(1.032-1.801) | 5270.221(4058.078-6836.287) | 1.833(1.412-2.378) | 0.81(0.77,0.86) |
| Lao People's Democratic Republic | 46.020(33.435-61.456) | 1.103(0.801-1.472) | 121.951(90.855-158.910) | 1.621(1.208-2.113) | 1.29(1.18,1.39) |
| Malaysia | 354.426(272.280-462.614) | 2.002(1.538-2.613) | 1043.119(791.989-1332.906) | 3.053(2.318-3.901) | 1.48(1.21,1.75) |
| Maldives | 2.644(1.973-3.433) | 1.189(0.888-1.544) | 13.465(10.084-17.664) | 2.481(1.858-3.255) | 2.31(2.26,2.35) |
| Myanmar | 530.866(387.730-701.404) | 1.312(0.958-1.733) | 972.237(733.977-1269.115) | 1.691(1.276-2.207) | 0.69(0.65,0.72) |
| Philippines | 569.710(427.130-752.549) | 0.901(0.675-1.190) | 1223.160(929.862-1592.412) | 1.068(0.812-1.391) | 0.37(0.31,0.42) |
| Sri Lanka | 371.475(280.199-488.426) | 2.175(1.640-2.860) | 711.587(549.925-888.182) | 3.141(2.427-3.920) | 1.16(0.92,1.40) |
| Thailand | 1091.296(824.301-1443.454) | 1.912(1.444-2.529) | 1868.131(1470.902-2352.772) | 2.842(2.238-3.580) | 1.17(1.05,1.28) |
| Timor-Leste | 9.208(6.588-12.392) | 1.179(0.844-1.587) | 20.418(15.399-26.504) | 1.423(1.073-1.847) | 0.42(0.27,0.58) |
| Viet Nam | 1021.766(750.992-1364.040) | 1.491(1.096-1.990) | 2323.199(1751.599-2943.120) | 2.252(1.698-2.853) | 1.31(1.24,1.38) |
| Armenia | 479.026(375.899-607.890) | 13.958(10.953-17.713) | 605.002(488.513-756.531) | 19.956(16.114-24.954) | 1.18(1.15,1.21) |
| Azerbaijan | 876.428(686.143-1117.185) | 11.909(9.323-15.180) | 1769.094(1393.152-2251.874) | 16.813(13.240-21.401) | 1.26(1.14,1.38) |
| Georgia | 961.861(759.023-1178.863) | 17.352(13.693-21.267) | 727.971(563.547-924.660) | 20.329(15.737-25.821) | 0.57(0.50,0.65) |
| Kazakhstan | 2303.113(1783.269-2881.199) | 13.589(10.522-17.000) | 3197.950(2540.675-4068.532) | 15.674(12.453-19.941) | 0.78(0.66,0.90) |
| Kyrgyzstan | 466.211(360.776-602.704) | 10.413(8.058-13.462) | 973.011(767.338-1221.741) | 13.420(10.583-16.851) | 0.84(0.77,0.92) |
| Mongolia | 184.217(142.646-233.678) | 8.514(6.593-10.800) | 426.737(334.005-551.901) | 12.325(9.647-15.940) | 1.32(1.19,1.44) |
| Tajikistan | 460.513(358.237-591.048) | 8.570(6.666-10.999) | 1145.731(902.011-1453.946) | 10.722(8.441-13.606) | 0.84(0.73,0.94) |
| Turkmenistan | 344.695(268.433-447.185) | 9.282(7.228-12.041) | 737.149(577.396-920.838) | 13.142(10.294-16.417) | 1.20(1.15,1.25) |
| Uzbekistan | 2094.974(1657.286-2718.109) | 10.048(7.949-13.037) | 5101.890(3936.345-6452.967) | 14.435(11.137-18.258) | 1.21(1.14,1.29) |
| Brunei Darussalam | 7.826(5.794-10.312) | 3.000(2.221-3.953) | 19.369(14.526-24.829) | 4.212(3.159-5.399) | 1.14(1.04,1.25) |
| Japan | 7500.094(5938.552-9238.532) | 6.064(4.801-7.469) | 11577.233(9448.087-14168.509) | 9.286(7.578-11.365) | 0.30(-0.17,0.78) |
| Republic of Korea | 7962.721(6207.076-10105.140) | 17.868(13.929-22.676) | 14781.371(11932.165-18488.381) | 28.591(23.080-35.762) | 1.56(1.07,2.05) |
| Singapore | 90.293(67.155-116.744) | 2.963(2.204-3.831) | 242.336(190.202-301.255) | 4.258(3.342-5.293) | 1.22(0.96,1.48) |
| Cyprus | 351.963(275.038-431.097) | 45.339(35.430-55.533) | 814.960(642.831-1007.230) | 59.572(46.989-73.626) | 0.78(0.61,0.95) |
| Israel | 1840.678(1539.365-2225.729) | 36.893(30.854-44.610) | 4668.132(3686.410-5661.018) | 46.729(36.902-56.668) | -0.09(-0.99,0.82) |
| Bahrain | 57.602(42.714-76.537) | 11.305(8.383-15.021) | 274.723(207.135-358.036) | 17.541(13.226-22.861) | 1.58(1.12,2.04) |
| Iran (Islamic Republic of) | 1832.313(1433.846-2375.515) | 3.208(2.511-4.160) | 5804.512(4537.673-7385.584) | 6.602(5.161-8.400) | 2.33(2.21,2.45) |
| Iraq | 946.061(717.064-1215.263) | 5.100(3.866-6.551) | 3995.397(3065.775-5067.715) | 9.044(6.940-11.471) | 1.59(1.52,1.65) |
| Jordan | 230.675(178.042-305.567) | 6.136(4.736-8.128) | 1544.370(1201.116-1960.790) | 11.700(9.099-14.854) | 1.98(1.95,2.01) |
| Kuwait | 147.410(111.945-193.363) | 8.660(6.577-11.360) | 645.974(499.179-837.846) | 13.804(10.667-17.904) | 1.81(1.70,1.92) |
| Lebanon | 594.783(462.571-758.581) | 18.709(14.550-23.861) | 1785.294(1380.656-2295.336) | 31.296(24.203-40.237) | 1.18(0.92,1.43) |
| Palestine | 107.659(83.459-141.172) | 5.228(4.053-6.855) | 482.084(362.333-619.720) | 8.914(6.700-11.459) | 1.55(1.50,1.60) |
| Oman | 121.398(93.323-159.846) | 6.079(4.673-8.004) | 671.241(508.786-863.963) | 13.074(9.910-16.828) | 2.42(2.33,2.51) |
| Qatar | 43.774(32.954-57.854) | 9.743(7.335-12.877) | 527.168(405.935-682.040) | 16.990(13.083-21.981) | 1.73(1.63,1.83) |
| Saudi Arabia | 955.950(728.735-1263.605) | 5.883(4.485-7.777) | 4623.576(3542.371-5803.300) | 14.016(10.738-17.592) | 2.08(1.81,2.34) |
| Syrian Arab Republic | 663.063(495.093-869.831) | 5.206(3.887-6.829) | 1789.619(1401.680-2227.995) | 10.064(7.883-12.530) | 1.99(1.92,2.06) |
| Türkiye | 4541.502(3452.203-5757.619) | 7.904(6.008-10.020) | 13554.151(10876.613-17190.538) | 15.785(12.667-20.020) | 1.91(1.49,2.34) |
| United Arab Emirates | 164.521(124.347-218.324) | 8.739(6.605-11.597) | 1923.756(1474.961-2500.884) | 18.989(14.559-24.685) | 2.42(2.36,2.48) |
| Afghanistan | 423.403(324.620-538.472) | 4.126(3.163-5.247) | 1914.832(1466.896-2513.638) | 5.283(4.048-6.936) | 0.68(0.53,0.83) |
| Bangladesh | 7956.872(6155.764-10054.983) | 7.294(5.643-9.218) | 24727.364(19732.279-30874.145) | 14.223(11.350-17.758) | 2.18(2.07,2.29) |
| Bhutan | 45.065(35.666-57.882) | 7.290(5.769-9.363) | 102.080(81.348-127.174) | 13.677(10.900-17.040) | 1.89(1.79,1.98) |
| India | 68767.547(54956.066-87004.092) | 8.135(6.501-10.292) | 196551.350(157221.714-245118.341) | 13.617(10.893-16.982) | 1.62(1.47,1.77) |
| Nepal | 1483.329(1159.646-1896.915) | 7.625(5.961-9.751) | 3922.194(3111.747-5002.932) | 12.626(10.017-16.105) | 1.50(1.34,1.65) |
| Pakistan | 7719.117(6131.567-9697.249) | 6.932(5.506-8.708) | 21027.338(16672.070-26848.838) | 8.620(6.834-11.006) | 0.58(0.44,0.71) |

**S12 Table: Deaths of CD in 1990 and 2023, and estimated annual percentage changes from 1990 to 2023.**

| Countries/  Regions | Number of cases, 1990 | Age-standardised rate per 100 000 population, 1990 | Number of cases, 2023 | Age-standardised rate per 100 000 population, 2023 | Estimated annual percentage change, 1990–2023 |
| --- | --- | --- | --- | --- | --- |
| East Asia | 274.140(152.815-454.783) | 0.020(0.011-0.033) | 338.657(213.303-503.612) | 0.021(0.013-0.031) | -0.11(-0.36,0.14) |
| Southeast Asia | 64.585(25.971-134.305) | 0.014(0.006-0.030) | 166.627(70.621-326.237) | 0.024(0.010-0.047) | 1.70(1.62,1.79) |
| South Asia | 442.894(173.814-901.241) | 0.040(0.016-0.081) | 776.229(332.712-1536.064) | 0.040(0.017-0.079) | -0.05(-0.14,0.05) |
| Central Asia | 37.658(25.408-55.841) | 0.073(0.049-0.109) | 51.857(37.622-70.073) | 0.065(0.047-0.088) | -0.36(-0.81,0.09) |
| West Asia | 147.085(76.331-253.127) | 0.074(0.038-0.127) | 221.005(119.530-359.425) | 0.065(0.036-0.105) | -0.54(-0.84,-0.24) |
| China | 166.307(78.010-300.008) | 0.014(0.007-0.025) | 207.098(119.128-318.838) | 0.014(0.008-0.022) | -0.35(-0.71,0.02) |
| Democratic People's Republic of Korea | 3.935(1.282-8.819) | 0.019(0.006-0.042) | 4.226(1.326-9.325) | 0.016(0.005-0.035) | -0.05(-0.31,0.22) |
| Cambodia | 0.988(0.296-2.369) | 0.010(0.003-0.023) | 2.413(0.857-5.342) | 0.014(0.005-0.031) | 1.41(1.19,1.63) |
| Indonesia | 36.075(13.813-77.483) | 0.019(0.007-0.042) | 83.625(33.596-165.886) | 0.029(0.012-0.058) | 1.17(1.06,1.28) |
| Lao People's Democratic Republic | 0.263(0.098-0.597) | 0.006(0.002-0.014) | 0.859(0.314-1.823) | 0.011(0.004-0.024) | 2.32(2.12,2.52) |
| Malaysia | 3.921(1.802-7.954) | 0.022(0.010-0.045) | 10.815(5.070-20.774) | 0.032(0.015-0.061) | 1.68(1.29,2.07) |
| Maldives | 0.045(0.018-0.096) | 0.020(0.008-0.043) | 0.207(0.107-0.364) | 0.038(0.020-0.067) | 2.46(2.24,2.68) |
| Myanmar | 7.416(2.998-16.191) | 0.018(0.007-0.040) | 17.000(6.453-36.669) | 0.030(0.011-0.064) | 1.71(1.57,1.85) |
| Philippines | 5.071(2.493-9.704) | 0.008(0.004-0.015) | 17.533(9.444-29.281) | 0.015(0.008-0.026) | 2.28(2.11,2.45) |
| Sri Lanka | 1.340(0.694-2.349) | 0.008(0.004-0.014) | 2.645(1.526-4.344) | 0.012(0.007-0.019) | 1.44(1.14,1.74) |
| Thailand | 2.665(1.130-5.212) | 0.005(0.002-0.009) | 7.809(3.826-13.707) | 0.012(0.006-0.021) | 2.59(2.45,2.73) |
| Timor-Leste | 0.075(0.028-0.152) | 0.010(0.004-0.019) | 0.211(0.084-0.406) | 0.015(0.006-0.028) | 1.45(1.16,1.74) |
| Viet Nam | 7.225(2.644-13.497) | 0.011(0.004-0.020) | 25.283(10.169-50.914) | 0.025(0.010-0.049) | 3.12(2.90,3.33) |
| Armenia | 2.388(1.500-3.512) | 0.070(0.044-0.102) | 1.272(0.916-1.786) | 0.042(0.030-0.059) | 0.01(-0.63,0.67) |
| Azerbaijan | 2.546(1.055-4.669) | 0.035(0.014-0.063) | 4.978(2.203-9.950) | 0.047(0.021-0.095) | 1.59(1.18,1.99) |
| Georgia | 24.316(16.010-38.366) | 0.439(0.289-0.692) | 4.783(3.562-6.493) | 0.134(0.099-0.181) | -4.30(-6.12,-2.43) |
| Kazakhstan | 20.902(14.619-29.242) | 0.123(0.086-0.173) | 26.008(19.016-34.045) | 0.127(0.093-0.167) | -0.11(-0.47,0.25) |
| Kyrgyzstan | 7.106(4.555-11.154) | 0.159(0.102-0.249) | 2.189(1.541-3.006) | 0.030(0.021-0.041) | -6.35(-7.98,-4.69) |
| Mongolia | 0.569(0.192-1.158) | 0.026(0.009-0.053) | 1.534(0.780-2.796) | 0.044(0.023-0.081) | 2.06(1.81,2.31) |
| Tajikistan | 0.021(0.009-0.046) | 0.000(0.000-0.001) | 0.045(0.018-0.097) | 0.000(0.000-0.001) | 0.06(-0.62,0.75) |
| Turkmenistan | 2.113(1.312-3.430) | 0.057(0.035-0.092) | 3.746(2.039-6.291) | 0.067(0.036-0.112) | 0.73(0.54,0.92) |
| Uzbekistan | 7.516(4.913-11.970) | 0.036(0.024-0.057) | 19.868(15.007-26.633) | 0.056(0.042-0.075) | 2.22(0.98,3.48) |
| Brunei Darussalam | 0.019(0.008-0.040) | 0.007(0.003-0.015) | 0.036(0.014-0.071) | 0.008(0.003-0.015) | 0.29(-0.10,0.67) |
| Japan | 78.865(61.399-100.448) | 0.064(0.050-0.081) | 103.194(79.120-138.213) | 0.083(0.063-0.111) | 1.15(0.59,1.71) |
| Republic of Korea | 24.464(11.932-44.351) | 0.055(0.027-0.100) | 22.605(12.949-34.440) | 0.044(0.025-0.067) | -0.78(-1.08,-0.47) |
| Singapore | 0.866(0.661-1.106) | 0.028(0.022-0.036) | 1.042(0.796-1.364) | 0.018(0.014-0.024) | -1.71(-2.41,-1.00) |
| Cyprus | 2.951(1.372-5.752) | 0.380(0.177-0.741) | 5.311(2.858-8.801) | 0.388(0.209-0.643) | -0.03(-0.23,0.17) |
| Israel | 8.175(6.493-9.955) | 0.164(0.130-0.200) | 21.847(16.338-27.702) | 0.219(0.164-0.277) | 0.73(0.50,0.97) |
| Bahrain | 0.163(0.084-0.285) | 0.032(0.016-0.056) | 0.575(0.284-1.048) | 0.037(0.018-0.067) | -0.15(-0.34,0.03) |
| Iran (Islamic Republic of) | 30.115(14.281-55.381) | 0.053(0.025-0.097) | 48.492(23.126-86.326) | 0.055(0.026-0.098) | -0.18(-0.46,0.11) |
| Iraq | 6.662(3.255-12.814) | 0.036(0.018-0.069) | 16.276(6.739-29.502) | 0.037(0.015-0.067) | 0.04(-0.08,0.16) |
| Jordan | 1.874(0.883-3.719) | 0.050(0.023-0.099) | 7.345(4.145-12.454) | 0.056(0.031-0.094) | 1.10(0.64,1.57) |
| Kuwait | 0.167(0.112-0.252) | 0.010(0.007-0.015) | 1.385(1.029-1.872) | 0.030(0.022-0.040) | 3.13(1.01,5.29) |
| Lebanon | 5.811(2.527-10.476) | 0.183(0.079-0.330) | 9.764(5.273-16.706) | 0.171(0.092-0.293) | -0.03(-0.35,0.30) |
| Palestine | 3.075(1.558-5.388) | 0.149(0.076-0.262) | 6.370(3.506-10.112) | 0.118(0.065-0.187) | -0.92(-1.09,-0.74) |
| Oman | 0.478(0.179-1.256) | 0.024(0.009-0.063) | 1.948(0.822-4.288) | 0.038(0.016-0.084) | 1.53(1.18,1.89) |
| Qatar | 0.090(0.031-0.219) | 0.020(0.007-0.049) | 0.475(0.238-0.812) | 0.015(0.008-0.026) | -1.51(-1.77,-1.24) |
| Saudi Arabia | 1.818(0.815-3.700) | 0.011(0.005-0.023) | 8.439(3.094-15.840) | 0.026(0.009-0.048) | 2.57(1.96,3.19) |
| Syrian Arab Republic | 2.690(0.790-6.148) | 0.021(0.006-0.048) | 3.410(1.047-7.647) | 0.019(0.006-0.043) | -0.37(-0.80,0.06) |
| Türkiye | 53.714(25.370-91.043) | 0.093(0.044-0.158) | 77.858(44.140-117.118) | 0.091(0.051-0.136) | -0.11(-0.40,0.17) |
| United Arab Emirates | 0.053(0.015-0.193) | 0.003(0.001-0.010) | 0.477(0.212-0.965) | 0.005(0.002-0.010) | 1.59(0.88,2.30) |
| Afghanistan | 2.792(0.886-6.669) | 0.027(0.009-0.065) | 6.495(2.290-12.066) | 0.018(0.006-0.033) | -1.19(-1.58,-0.80) |
| Bangladesh | 54.691(19.335-113.505) | 0.050(0.018-0.104) | 92.236(34.502-206.336) | 0.053(0.020-0.119) | 0.40(0.18,0.62) |
| Bhutan | 0.262(0.088-0.627) | 0.042(0.014-0.101) | 0.484(0.160-1.094) | 0.065(0.021-0.147) | 1.09(0.96,1.23) |
| India | 324.418(131.697-647.604) | 0.038(0.016-0.077) | 540.233(242.777-1034.864) | 0.037(0.017-0.072) | -0.11(-0.23,0.01) |
| Nepal | 10.191(3.132-22.690) | 0.052(0.016-0.117) | 18.223(6.977-38.654) | 0.059(0.022-0.124) | 0.32(0.03,0.62) |
| Pakistan | 49.155(17.965-107.699) | 0.044(0.016-0.097) | 115.705(44.374-238.341) | 0.047(0.018-0.098) | -0.12(-0.26,0.01) |

**S13 Table: SDI for Asia, East Asia, Southeast Asia, South Asia, Central Asia, and West Asia from 1990 to 2023.**

| year | Asia | East Asia | Southeast Asia | South Asia | Central Asia | West Asia |
| --- | --- | --- | --- | --- | --- | --- |
| 1990 | 0.435 | 0.503 | 0.454 | 0.328 | 0.540 | 0.484 |
| 1991 | 0.441 | 0.511 | 0.461 | 0.334 | 0.542 | 0.494 |
| 1992 | 0.448 | 0.518 | 0.468 | 0.340 | 0.544 | 0.504 |
| 1993 | 0.454 | 0.526 | 0.476 | 0.345 | 0.547 | 0.513 |
| 1994 | 0.461 | 0.533 | 0.483 | 0.351 | 0.551 | 0.521 |
| 1995 | 0.467 | 0.541 | 0.491 | 0.357 | 0.555 | 0.529 |
| 1996 | 0.475 | 0.551 | 0.498 | 0.364 | 0.559 | 0.537 |
| 1997 | 0.482 | 0.560 | 0.506 | 0.370 | 0.562 | 0.545 |
| 1998 | 0.488 | 0.568 | 0.512 | 0.376 | 0.565 | 0.552 |
| 1999 | 0.495 | 0.576 | 0.518 | 0.383 | 0.569 | 0.560 |
| 2000 | 0.501 | 0.583 | 0.523 | 0.389 | 0.572 | 0.568 |
| 2001 | 0.506 | 0.589 | 0.528 | 0.395 | 0.577 | 0.575 |
| 2002 | 0.512 | 0.595 | 0.533 | 0.401 | 0.581 | 0.583 |
| 2003 | 0.517 | 0.602 | 0.538 | 0.407 | 0.585 | 0.589 |
| 2004 | 0.523 | 0.609 | 0.543 | 0.413 | 0.590 | 0.597 |
| 2005 | 0.530 | 0.617 | 0.549 | 0.420 | 0.594 | 0.605 |
| 2006 | 0.537 | 0.627 | 0.554 | 0.427 | 0.599 | 0.613 |
| 2007 | 0.545 | 0.636 | 0.560 | 0.435 | 0.603 | 0.621 |
| 2008 | 0.553 | 0.645 | 0.566 | 0.443 | 0.607 | 0.629 |
| 2009 | 0.560 | 0.653 | 0.572 | 0.451 | 0.612 | 0.636 |
| 2010 | 0.569 | 0.664 | 0.578 | 0.460 | 0.616 | 0.643 |
| 2011 | 0.577 | 0.673 | 0.585 | 0.469 | 0.620 | 0.650 |
| 2012 | 0.583 | 0.677 | 0.591 | 0.479 | 0.623 | 0.656 |
| 2013 | 0.590 | 0.681 | 0.598 | 0.490 | 0.626 | 0.662 |
| 2014 | 0.597 | 0.685 | 0.604 | 0.501 | 0.629 | 0.668 |
| 2015 | 0.603 | 0.687 | 0.611 | 0.512 | 0.632 | 0.675 |
| 2016 | 0.610 | 0.690 | 0.617 | 0.522 | 0.635 | 0.682 |
| 2017 | 0.618 | 0.697 | 0.623 | 0.533 | 0.637 | 0.689 |
| 2018 | 0.626 | 0.705 | 0.629 | 0.542 | 0.639 | 0.696 |
| 2019 | 0.634 | 0.713 | 0.635 | 0.551 | 0.640 | 0.703 |
| 2020 | 0.641 | 0.722 | 0.641 | 0.559 | 0.641 | 0.709 |
| 2021 | 0.647 | 0.729 | 0.645 | 0.566 | 0.643 | 0.716 |
| 2022 | 0.654 | 0.735 | 0.650 | 0.574 | 0.645 | 0.722 |
| 2023 | 0.660 | 0.741 | 0.655 | 0.581 | 0.649 | 0.729 |

**S14 Table: Projected age-standardized incidence, prevalence, and mortality rates for IBD in Asia through 2050.**

| IBD | | | | | | | | | |
| --- | --- | --- | --- | --- | --- | --- | --- | --- | --- |
|  | Age-standardized  incidence rate | | | Age-standardized prevalence rate | | | Age-standardized  death rate | | |
| Year | Forecast | Lower  _95 | Upper  _95 | Forecast | Lower  _95 | Upper  _95 | Forecast | Lower  _95 | Upper  _95 |
| 2024 | 3.425 | 3.412 | 3.439 | 30.871 | 30.683 | 31.058 | 0.295 | 0.284 | 0.306 |
| 2025 | 3.430 | 3.383 | 3.476 | 31.981 | 31.450 | 32.512 | 0.295 | 0.280 | 0.311 |
| 2026 | 3.444 | 3.352 | 3.536 | 32.839 | 31.842 | 33.836 | 0.295 | 0.276 | 0.314 |
| 2027 | 3.475 | 3.335 | 3.616 | 33.394 | 31.870 | 34.918 | 0.295 | 0.273 | 0.317 |
| 2028 | 3.516 | 3.334 | 3.698 | 33.665 | 31.619 | 35.710 | 0.295 | 0.270 | 0.320 |
| 2029 | 3.557 | 3.341 | 3.773 | 33.727 | 31.221 | 36.234 | 0.295 | 0.268 | 0.322 |
| 2030 | 3.598 | 3.352 | 3.843 | 33.690 | 30.814 | 36.565 | 0.295 | 0.266 | 0.325 |
| 2031 | 3.638 | 3.367 | 3.910 | 33.664 | 30.521 | 36.807 | 0.295 | 0.264 | 0.327 |
| 2032 | 3.679 | 3.384 | 3.974 | 33.744 | 30.422 | 37.066 | 0.295 | 0.262 | 0.329 |
| 2033 | 3.720 | 3.403 | 4.037 | 33.987 | 30.552 | 37.422 | 0.295 | 0.260 | 0.330 |
| 2034 | 3.760 | 3.423 | 4.098 | 34.408 | 30.900 | 37.915 | 0.295 | 0.258 | 0.332 |
| 2035 | 3.801 | 3.444 | 4.158 | 34.983 | 31.423 | 38.543 | 0.295 | 0.257 | 0.334 |
| 2036 | 3.842 | 3.466 | 4.217 | 35.660 | 32.052 | 39.268 | 0.295 | 0.255 | 0.335 |
| 2037 | 3.883 | 3.490 | 4.276 | 36.374 | 32.711 | 40.037 | 0.295 | 0.254 | 0.337 |
| 2038 | 3.923 | 3.513 | 4.333 | 37.060 | 33.325 | 40.794 | 0.295 | 0.252 | 0.338 |
| 2039 | 3.964 | 3.538 | 4.390 | 37.670 | 33.841 | 41.499 | 0.295 | 0.251 | 0.340 |
| 2040 | 4.005 | 3.563 | 4.446 | 38.178 | 34.231 | 42.125 | 0.295 | 0.250 | 0.341 |
| 2041 | 4.045 | 3.589 | 4.502 | 38.585 | 34.502 | 42.667 | 0.295 | 0.248 | 0.342 |
| 2042 | 4.086 | 3.615 | 4.557 | 38.910 | 34.684 | 43.135 | 0.295 | 0.247 | 0.344 |
| 2043 | 4.127 | 3.642 | 4.612 | 39.187 | 34.825 | 43.550 | 0.295 | 0.246 | 0.345 |
| 2044 | 4.167 | 3.669 | 4.666 | 39.457 | 34.971 | 43.944 | 0.295 | 0.244 | 0.346 |
| 2045 | 4.208 | 3.696 | 4.720 | 39.754 | 35.161 | 44.348 | 0.295 | 0.243 | 0.347 |
| 2046 | 4.249 | 3.724 | 4.774 | 40.103 | 35.418 | 44.787 | 0.295 | 0.242 | 0.348 |
| 2047 | 4.290 | 3.752 | 4.827 | 40.512 | 35.751 | 45.273 | 0.295 | 0.241 | 0.350 |
| 2048 | 4.330 | 3.780 | 4.880 | 40.978 | 36.148 | 45.808 | 0.295 | 0.240 | 0.351 |
| 2049 | 4.371 | 3.809 | 4.933 | 41.486 | 36.591 | 46.381 | 0.295 | 0.239 | 0.352 |
| 2050 | 4.412 | 3.838 | 4.986 | 42.014 | 37.054 | 46.975 | 0.295 | 0.238 | 0.353 |

**Note:** Values are ARIMA point projections with corresponding 95% prediction intervals. The prediction intervals quantify ARIMA forecast uncertainty conditional on the historical GBD point-estimate series and do not incorporate uncertainty in the underlying GBD estimates. PI, prediction interval; IBD, inflammatory bowel disease; UC, ulcerative colitis; CD, Crohn’s disease.

**S15 Table: Projected age-standardized incidence, prevalence, and mortality rates for UC in Asia through 2050.**

| UC | | | | | | | | | |
| --- | --- | --- | --- | --- | --- | --- | --- | --- | --- |
|  | Age-standardized  incidence rate | | | Age-standardized prevalence rate | | | Age-standardized  death rate | | |
| Year | Forecast | Lower  _95 | Upper  _95 | Forecast | Lower  _95 | Upper  _95 | Forecast | Lower  _95 | Upper  _95 |
| 2024 | 2.479 | 2.470 | 2.488 | 21.877 | 21.757 | 21.996 | 0.249 | 0.240 | 0.259 |
| 2025 | 2.486 | 2.455 | 2.517 | 22.773 | 22.430 | 23.116 | 0.255 | 0.238 | 0.272 |
| 2026 | 2.499 | 2.437 | 2.560 | 23.527 | 22.871 | 24.183 | 0.255 | 0.235 | 0.275 |
| 2027 | 2.522 | 2.428 | 2.615 | 24.076 | 23.053 | 25.099 | 0.255 | 0.232 | 0.278 |
| 2028 | 2.551 | 2.431 | 2.672 | 24.404 | 23.002 | 25.806 | 0.255 | 0.230 | 0.280 |
| 2029 | 2.581 | 2.438 | 2.724 | 24.536 | 22.780 | 26.292 | 0.255 | 0.228 | 0.282 |
| 2030 | 2.611 | 2.448 | 2.774 | 24.531 | 22.474 | 26.587 | 0.255 | 0.226 | 0.284 |
| 2031 | 2.641 | 2.461 | 2.821 | 24.463 | 22.173 | 26.753 | 0.255 | 0.224 | 0.286 |
| 2032 | 2.670 | 2.475 | 2.866 | 24.411 | 21.955 | 26.867 | 0.255 | 0.222 | 0.288 |
| 2033 | 2.700 | 2.490 | 2.910 | 24.438 | 21.872 | 27.004 | 0.255 | 0.220 | 0.290 |
| 2034 | 2.730 | 2.506 | 2.954 | 24.588 | 21.953 | 27.223 | 0.255 | 0.219 | 0.291 |
| 2035 | 2.759 | 2.523 | 2.996 | 24.875 | 22.195 | 27.555 | 0.255 | 0.217 | 0.293 |
| 2036 | 2.789 | 2.540 | 3.038 | 25.286 | 22.574 | 27.999 | 0.255 | 0.216 | 0.294 |
| 2037 | 2.819 | 2.558 | 3.079 | 25.790 | 23.048 | 28.531 | 0.255 | 0.214 | 0.296 |
| 2038 | 2.849 | 2.577 | 3.120 | 26.339 | 23.563 | 29.115 | 0.255 | 0.213 | 0.297 |
| 2039 | 2.878 | 2.596 | 3.161 | 26.887 | 24.066 | 29.708 | 0.255 | 0.212 | 0.298 |
| 2040 | 2.908 | 2.615 | 3.201 | 27.390 | 24.508 | 30.273 | 0.255 | 0.210 | 0.300 |
| 2041 | 2.938 | 2.635 | 3.240 | 27.822 | 24.860 | 30.783 | 0.255 | 0.209 | 0.301 |
| 2042 | 2.967 | 2.655 | 3.280 | 28.169 | 25.113 | 31.225 | 0.255 | 0.208 | 0.302 |
| 2043 | 2.997 | 2.675 | 3.319 | 28.436 | 25.277 | 31.596 | 0.255 | 0.207 | 0.303 |
| 2044 | 3.027 | 2.696 | 3.358 | 28.643 | 25.380 | 31.907 | 0.255 | 0.206 | 0.304 |
| 2045 | 3.057 | 2.717 | 3.396 | 28.817 | 25.456 | 32.178 | 0.255 | 0.204 | 0.306 |
| 2046 | 3.086 | 2.738 | 3.434 | 28.987 | 25.541 | 32.433 | 0.255 | 0.203 | 0.307 |
| 2047 | 3.116 | 2.759 | 3.473 | 29.181 | 25.663 | 32.699 | 0.255 | 0.202 | 0.308 |
| 2048 | 3.146 | 2.781 | 3.511 | 29.417 | 25.839 | 32.995 | 0.255 | 0.201 | 0.309 |
| 2049 | 3.175 | 2.803 | 3.548 | 29.705 | 26.076 | 33.334 | 0.255 | 0.200 | 0.310 |
| 2050 | 3.205 | 2.824 | 3.586 | 30.043 | 26.369 | 33.717 | 0.255 | 0.199 | 0.311 |

**Note:** Values are ARIMA point projections with corresponding 95% prediction intervals. The prediction intervals quantify ARIMA forecast uncertainty conditional on the historical GBD point-estimate series and do not incorporate uncertainty in the underlying GBD estimates. PI, prediction interval; IBD, inflammatory bowel disease; UC, ulcerative colitis; CD, Crohn’s disease.

**S16 Table: Projected age-standardized incidence, prevalence, and mortality rates for CD in Asia through 2050.**

| CD | | | | | | | | | |
| --- | --- | --- | --- | --- | --- | --- | --- | --- | --- |
|  | Age-standardized  incidence rate | | | Age-standardized  prevalence rate | | | Age-standardized  death rate | | |
| Year | Forecast | Lower  _95 | Upper  _95 | Forecast | Lower  _95 | Upper  _95 | Forecast | Lower  _95 | Upper  _95 |
| 2024 | 0.946 | 0.941 | 0.951 | 9.015 | 8.945 | 9.084 | 0.032 | 0.031 | 0.033 |
| 2025 | 0.944 | 0.927 | 0.960 | 9.275 | 9.064 | 9.486 | 0.032 | 0.031 | 0.034 |
| 2026 | 0.945 | 0.913 | 0.978 | 9.458 | 9.060 | 9.856 | 0.032 | 0.030 | 0.034 |
| 2027 | 0.954 | 0.904 | 1.003 | 9.571 | 8.973 | 10.168 | 0.032 | 0.030 | 0.034 |
| 2028 | 0.965 | 0.901 | 1.029 | 9.638 | 8.854 | 10.422 | 0.032 | 0.030 | 0.035 |
| 2029 | 0.976 | 0.900 | 1.052 | 9.688 | 8.747 | 10.628 | 0.032 | 0.030 | 0.035 |
| 2030 | 0.987 | 0.900 | 1.074 | 9.745 | 8.683 | 10.806 | 0.032 | 0.030 | 0.035 |
| 2031 | 0.998 | 0.902 | 1.094 | 9.823 | 8.671 | 10.976 | 0.032 | 0.030 | 0.035 |
| 2032 | 1.009 | 0.904 | 1.113 | 9.929 | 8.709 | 11.149 | 0.032 | 0.030 | 0.035 |
| 2033 | 1.020 | 0.908 | 1.132 | 10.059 | 8.786 | 11.332 | 0.032 | 0.030 | 0.035 |
| 2034 | 1.031 | 0.911 | 1.150 | 10.203 | 8.885 | 11.522 | 0.032 | 0.030 | 0.035 |
| 2035 | 1.042 | 0.915 | 1.168 | 10.354 | 8.992 | 11.715 | 0.032 | 0.029 | 0.035 |
| 2036 | 1.053 | 0.920 | 1.186 | 10.501 | 9.095 | 11.907 | 0.032 | 0.029 | 0.035 |
| 2037 | 1.064 | 0.925 | 1.203 | 10.641 | 9.188 | 12.093 | 0.032 | 0.029 | 0.035 |
| 2038 | 1.075 | 0.930 | 1.220 | 10.771 | 9.269 | 12.273 | 0.032 | 0.029 | 0.035 |
| 2039 | 1.086 | 0.935 | 1.237 | 10.893 | 9.340 | 12.446 | 0.032 | 0.029 | 0.036 |
| 2040 | 1.097 | 0.940 | 1.253 | 11.011 | 9.407 | 12.614 | 0.032 | 0.029 | 0.036 |
| 2041 | 1.108 | 0.946 | 1.269 | 11.126 | 9.473 | 12.780 | 0.032 | 0.029 | 0.036 |
| 2042 | 1.119 | 0.952 | 1.286 | 11.243 | 9.542 | 12.943 | 0.032 | 0.029 | 0.036 |
| 2043 | 1.130 | 0.958 | 1.302 | 11.362 | 9.617 | 13.107 | 0.032 | 0.029 | 0.036 |
| 2044 | 1.141 | 0.964 | 1.317 | 11.485 | 9.697 | 13.272 | 0.032 | 0.029 | 0.036 |
| 2045 | 1.152 | 0.970 | 1.333 | 11.610 | 9.781 | 13.438 | 0.032 | 0.029 | 0.036 |
| 2046 | 1.163 | 0.977 | 1.349 | 11.737 | 9.869 | 13.604 | 0.032 | 0.029 | 0.036 |
| 2047 | 1.174 | 0.983 | 1.364 | 11.864 | 9.958 | 13.770 | 0.032 | 0.029 | 0.036 |
| 2048 | 1.185 | 0.990 | 1.380 | 11.991 | 10.047 | 13.935 | 0.032 | 0.028 | 0.036 |
| 2049 | 1.196 | 0.996 | 1.395 | 12.117 | 10.136 | 14.099 | 0.032 | 0.028 | 0.036 |
| 2050 | 1.207 | 1.003 | 1.410 | 12.242 | 10.224 | 14.261 | 0.032 | 0.028 | 0.036 |

**Note:** Values are ARIMA point projections with corresponding 95% prediction intervals. The prediction intervals quantify ARIMA forecast uncertainty conditional on the historical GBD point-estimate series and do not incorporate uncertainty in the underlying GBD estimates. PI, prediction interval; IBD, inflammatory bowel disease; UC, ulcerative colitis; CD, Crohn’s disease.

**S17 Table. Country-level associations between the Socio-Demographic Index and age-standardized IBD burden measures in Asia, 2023.**

Panel A. Linear regression effect estimates and assessment of non-linearity.

| **Outcome** | **N** | **β per 0.1 SDI (95% CI)** | **P value** | **FDR-adjusted P value** | **R²** | **Adjusted R²** | **Linear AIC** | **Spline AIC** | **P for non-linearity** | **FDR-adjusted P for non-linearity** |
| --- | --- | --- | --- | --- | --- | --- | --- | --- | --- | --- |
| ASIR | 47 | 0.598 (-0.009 to 1.205) | 0.054 | 0.107 | 0.080 | 0.060 | 231.58 | 234.65 | 0.655 | 0.889 |
| ASPR | 47 | 10.048 (3.642 to 16.454) | 0.003 | 0.011 | 0.182 | 0.163 | 453.04 | 455.59 | 0.517 | 0.889 |
| ASDR | 47 | 1.257 (-0.433 to 2.948) | 0.141 | 0.188 | 0.047 | 0.026 | 327.81 | 331.56 | 0.889 | 0.889 |
| ASMR | 47 | 0.032 (-0.020 to 0.084) | 0.220 | 0.220 | 0.033 | 0.012 | 0.73 | 4.16 | 0.769 | 0.889 |

Panel B. Sensitivity analyses using alternative correlation methods and exclusion criteria.

| **Outcome** | **Analysis scenario** | **Method** | **N** | **Correlation coefficient** | **95% CI** | **P value** | **FDR-adjusted**  **P value** |
| --- | --- | --- | --- | --- | --- | --- | --- |
| **ASIR** | **All countries** | **Pearson** | **47** | **0.283** | **-0.004 to 0.528** | **0.054** | **0.107** |
|  |  | Spearman | 47 | 0.236 | — | 0.110 | 0.220 |
|  | Excluding high-uncertainty countries | Pearson | 35 | 0.243 | -0.098 to 0.533 | 0.160 | 0.258 |
|  |  | Spearman | 35 | 0.180 | — | 0.300 | 0.599 |
|  | Excluding Afghanistan, Myanmar, and Lao PDR | Pearson | 44 | 0.218 | -0.084 to 0.484 | 0.155 | 0.309 |
|  |  | Spearman | 44 | 0.143 | — | 0.355 | 0.710 |
| **ASPR** | **All countries** | **Pearson** | **47** | **0.426** | **0.158 to 0.635** | **0.003** | **0.011** |
|  |  | Spearman | 47 | 0.424 | — | 0.003 | 0.012 |
|  | Excluding high-uncertainty countries | Pearson | 35 | 0.379 | 0.053 to 0.632 | 0.025 | 0.099 |
|  |  | Spearman | 35 | 0.404 | — | 0.016 | 0.064 |
|  | Excluding Afghanistan, Myanmar, and Lao PDR | Pearson | 44 | 0.379 | 0.092 to 0.607 | 0.011 | 0.045 |
|  |  | Spearman | 44 | 0.367 | — | 0.014 | 0.058 |
| **ASDR** | **All countries** | **Pearson** | **47** | **0.218** | **-0.074 to 0.475** | **0.141** | **0.188** |
|  |  | Spearman | 47 | 0.113 | — | 0.448 | 0.597 |
|  | Excluding high-uncertainty countries | Pearson | 35 | 0.149 | -0.194 to 0.459 | 0.393 | 0.393 |
|  |  | Spearman | 35 | 0.005 | — | 0.978 | 0.978 |
|  | Excluding Afghanistan, Myanmar, and Lao PDR | Pearson | 44 | 0.135 | -0.168 to 0.415 | 0.382 | 0.428 |
|  |  | Spearman | 44 | 0.021 | — | 0.893 | 0.893 |
| **ASMR** | **All countries** | **Pearson** | **47** | **0.182** | **-0.111 to 0.446** | **0.220** | **0.220** |
|  |  | Spearman | 47 | 0.015 | — | 0.921 | 0.921 |
|  | Excluding high-uncertainty countries | Pearson | 35 | 0.225 | -0.117 to 0.520 | 0.193 | 0.258 |
|  |  | Spearman | 35 | 0.014 | — | 0.936 | 0.978 |
|  | Excluding Afghanistan, Myanmar, and Lao PDR | Pearson | 44 | 0.123 | -0.181 to 0.405 | 0.428 | 0.428 |
|  |  | Spearman | 44 | -0.058 | — | 0.706 | 0.893 |

Note: Effect estimates in Panel A represent the absolute change in the age-standardized rate per 0.1-unit increase in SDI. Linear models were compared with natural cubic spline models with three degrees of freedom using partial F tests and AIC. High-uncertainty countries were defined as those in the highest quartile of relative uncertainty, calculated as (upper UI - lower UI)/point estimate. P values were adjusted across the four burden measures using the Benjamini-Hochberg false discovery rate procedure. Spearman 95% CIs were not estimated and are shown as em dashes. These country-level cross-sectional ecological associations do not establish causality. Abbreviations: AIC, Akaike information criterion; ASDR, age-standardized DALY rate; ASIR, age-standardized incidence rate; ASMR, age-standardized mortality rate; ASPR, age-standardized prevalence rate; CI, confidence interval; DALY, disability-adjusted life year; FDR, false discovery rate; SDI, Socio-Demographic Index; UI, uncertainty interval.

**S18 Table. Sensitivity analyses of temporal trends in IBD burden from 1990 to 2023.**

| Location | Measure | Point-estimate EAPC | Lower-bound EAPC | Upper-bound EAPC | Direction consistent |
| --- | --- | --- | --- | --- | --- |
| Asia | Deaths | -0.130 | 0.483 | -0.486 | FALSE |
| Asia | DALYs | -0.192 | 0.370 | -0.602 | FALSE |
| Asia | Prevalence | 1.945 | 1.948 | 1.951 | TRUE |
| Asia | Incidence | 1.952 | 1.924 | 1.954 | TRUE |
| East Asia | Deaths | -0.211 | 0.892 | -0.817 | FALSE |
| East Asia | DALYs | -0.903 | 0.336 | -1.551 | FALSE |
| East Asia | Prevalence | 2.425 | 2.420 | 2.432 | TRUE |
| East Asia | Incidence | 2.808 | 2.747 | 2.812 | TRUE |
| Southeast Asia | Deaths | 0.450 | 0.938 | -0.103 | FALSE |
| Southeast Asia | DALYs | 0.017 | 0.588 | -0.432 | FALSE |
| Southeast Asia | Prevalence | 1.164 | 1.200 | 1.128 | TRUE |
| Southeast Asia | Incidence | 1.131 | 1.102 | 1.149 | TRUE |
| South Asia | Deaths | -0.085 | 0.072 | -0.215 | FALSE |
| South Asia | DALYs | 0.116 | 0.236 | -0.163 | FALSE |
| South Asia | Prevalence | 1.512 | 1.518 | 1.517 | TRUE |
| South Asia | Incidence | 1.412 | 1.401 | 1.412 | TRUE |
| Central Asia | Deaths | -0.551 | -0.435 | -0.641 | TRUE |
| Central Asia | DALYs | -0.235 | -0.242 | -0.209 | TRUE |
| Central Asia | Prevalence | 1.058 | 1.016 | 1.093 | TRUE |
| Central Asia | Incidence | 1.173 | 1.096 | 1.160 | TRUE |
| West Asia | Deaths | 0.023 | 0.504 | -0.476 | FALSE |
| West Asia | DALYs | -0.009 | 0.334 | -0.422 | FALSE |
| West Asia | Prevalence | 1.592 | 1.590 | 1.605 | TRUE |
| West Asia | Incidence | 1.314 | 1.236 | 1.343 | TRUE |

**S19 Table. Country-level relative uncertainty in age-standardized IBD burden estimates in Asia, 2023.**

| Measure | Country | Point estimate | Lower bound | Upper bound | Relative uncertainty |
| --- | --- | --- | --- | --- | --- |
| DALYs | Myanmar | 10.306 | 5.105 | 18.028 | 1.254 |
| DALYs | Cambodia | 5.850 | 3.103 | 9.711 | 1.130 |
| DALYs | Malaysia | 10.111 | 4.842 | 16.255 | 1.129 |
| DALYs | Democratic People's Republic of Korea | 8.714 | 4.821 | 14.161 | 1.072 |
| DALYs | Timor-Leste | 6.472 | 3.681 | 10.389 | 1.036 |
| DALYs | Lao People's Democratic Republic | 4.048 | 2.556 | 6.372 | 0.943 |
| DALYs | Bhutan | 20.257 | 12.223 | 31.101 | 0.932 |
| DALYs | Indonesia | 9.500 | 5.770 | 14.547 | 0.924 |
| DALYs | Viet Nam | 5.760 | 3.712 | 8.940 | 0.908 |
| DALYs | Pakistan | 15.667 | 9.997 | 23.981 | 0.893 |
| DALYs | Maldives | 5.856 | 4.007 | 8.637 | 0.791 |
| DALYs | United Arab Emirates | 10.252 | 6.815 | 14.843 | 0.783 |
| DALYs | Afghanistan | 6.506 | 4.434 | 9.467 | 0.774 |
| DALYs | Bangladesh | 21.520 | 13.438 | 29.379 | 0.741 |
| DALYs | Nepal | 17.990 | 12.348 | 25.355 | 0.723 |
| DALYs | Qatar | 11.448 | 8.167 | 15.902 | 0.676 |
| DALYs | Brunei Darussalam | 14.468 | 10.114 | 19.889 | 0.676 |
| DALYs | Tajikistan | 16.081 | 11.244 | 22.105 | 0.675 |
| DALYs | Republic of Korea | 19.555 | 13.892 | 26.938 | 0.667 |
| DALYs | Saudi Arabia | 7.449 | 5.068 | 10.011 | 0.664 |
| DALYs | Kuwait | 10.006 | 7.355 | 13.948 | 0.659 |
| DALYs | Oman | 11.888 | 8.582 | 16.401 | 0.658 |
| DALYs | Iraq | 11.349 | 8.015 | 15.447 | 0.655 |
| DALYs | Philippines | 5.312 | 3.879 | 7.249 | 0.634 |
| DALYs | Thailand | 5.029 | 3.758 | 6.938 | 0.632 |
| DALYs | Bahrain | 11.035 | 7.961 | 14.817 | 0.621 |
| DALYs | Mongolia | 17.201 | 12.503 | 23.088 | 0.615 |
| DALYs | Azerbaijan | 13.783 | 9.973 | 18.300 | 0.604 |
| DALYs | Iran (Islamic Republic of) | 14.089 | 10.262 | 18.756 | 0.603 |
| DALYs | Jordan | 10.671 | 7.978 | 14.331 | 0.595 |
| DALYs | Syrian Arab Republic | 10.795 | 7.826 | 14.174 | 0.588 |
| DALYs | Israel | 30.375 | 22.870 | 40.666 | 0.586 |
| DALYs | India | 14.815 | 10.686 | 19.323 | 0.583 |
| DALYs | Palestine | 12.954 | 9.682 | 16.960 | 0.562 |
| DALYs | Turkmenistan | 18.993 | 14.453 | 24.529 | 0.531 |
| DALYs | Lebanon | 30.624 | 23.234 | 39.443 | 0.529 |
| DALYs | Sri Lanka | 2.961 | 2.218 | 3.783 | 0.528 |
| DALYs | Cyprus | 43.582 | 33.420 | 56.304 | 0.525 |
| DALYs | China | 8.607 | 6.697 | 11.132 | 0.515 |
| DALYs | Armenia | 17.556 | 13.752 | 22.258 | 0.485 |
| DALYs | Kyrgyzstan | 12.712 | 10.021 | 15.977 | 0.469 |
| DALYs | Türkiye | 16.652 | 13.093 | 20.698 | 0.457 |
| DALYs | Georgia | 18.814 | 14.871 | 23.437 | 0.455 |
| DALYs | Singapore | 3.642 | 2.910 | 4.509 | 0.439 |
| DALYs | Japan | 14.133 | 11.264 | 17.285 | 0.426 |
| DALYs | Uzbekistan | 16.131 | 13.287 | 19.257 | 0.370 |
| DALYs | Kazakhstan | 21.240 | 17.580 | 25.002 | 0.349 |
| Deaths | Bhutan | 0.462 | 0.207 | 0.865 | 1.422 |
| Deaths | Malaysia | 0.358 | 0.133 | 0.629 | 1.385 |
| Deaths | Cambodia | 0.171 | 0.077 | 0.304 | 1.321 |
| Deaths | Myanmar | 0.227 | 0.103 | 0.395 | 1.283 |
| Deaths | Republic of Korea | 0.421 | 0.265 | 0.797 | 1.266 |
| Deaths | Democratic People's Republic of Korea | 0.307 | 0.149 | 0.532 | 1.250 |
| Deaths | Saudi Arabia | 0.074 | 0.031 | 0.122 | 1.234 |
| Deaths | Bangladesh | 0.421 | 0.180 | 0.695 | 1.222 |
| Deaths | Timor-Leste | 0.190 | 0.100 | 0.332 | 1.221 |
| Deaths | Pakistan | 0.333 | 0.180 | 0.562 | 1.147 |
| Deaths | Lao People's Democratic Republic | 0.105 | 0.058 | 0.177 | 1.138 |
| Deaths | Iran (Islamic Republic of) | 0.291 | 0.156 | 0.487 | 1.138 |
| Deaths | United Arab Emirates | 0.015 | 0.009 | 0.025 | 1.084 |
| Deaths | Nepal | 0.375 | 0.214 | 0.620 | 1.081 |
| Deaths | Oman | 0.153 | 0.086 | 0.248 | 1.063 |
| Deaths | Iraq | 0.182 | 0.100 | 0.292 | 1.051 |
| Deaths | Viet Nam | 0.193 | 0.110 | 0.309 | 1.032 |
| Deaths | Maldives | 0.169 | 0.104 | 0.274 | 1.005 |
| Deaths | Afghanistan | 0.095 | 0.054 | 0.147 | 0.984 |
| Deaths | Tajikistan | 0.243 | 0.146 | 0.383 | 0.976 |
| Deaths | Indonesia | 0.296 | 0.174 | 0.461 | 0.969 |
| Deaths | Syrian Arab Republic | 0.199 | 0.112 | 0.300 | 0.946 |
| Deaths | Azerbaijan | 0.136 | 0.087 | 0.216 | 0.945 |
| Deaths | India | 0.250 | 0.155 | 0.390 | 0.938 |
| Deaths | Jordan | 0.125 | 0.073 | 0.189 | 0.929 |
| Deaths | Qatar | 0.081 | 0.048 | 0.121 | 0.897 |
| Deaths | Bahrain | 0.132 | 0.079 | 0.197 | 0.896 |
| Deaths | Cyprus | 1.557 | 1.052 | 2.411 | 0.873 |
| Deaths | Sri Lanka | 0.062 | 0.038 | 0.090 | 0.850 |
| Deaths | Lebanon | 0.576 | 0.370 | 0.849 | 0.833 |
| Deaths | Mongolia | 0.298 | 0.192 | 0.440 | 0.832 |
| Deaths | Thailand | 0.168 | 0.107 | 0.243 | 0.809 |
| Deaths | Palestine | 0.271 | 0.174 | 0.390 | 0.797 |
| Deaths | Brunei Darussalam | 0.438 | 0.286 | 0.635 | 0.796 |
| Deaths | Turkmenistan | 0.288 | 0.201 | 0.408 | 0.720 |
| Deaths | Philippines | 0.139 | 0.098 | 0.194 | 0.688 |
| Deaths | China | 0.342 | 0.245 | 0.470 | 0.658 |
| Deaths | Türkiye | 0.415 | 0.295 | 0.534 | 0.576 |
| Deaths | Kuwait | 0.087 | 0.069 | 0.108 | 0.447 |
| Deaths | Georgia | 0.308 | 0.245 | 0.383 | 0.446 |
| Deaths | Japan | 0.487 | 0.380 | 0.590 | 0.431 |
| Deaths | Singapore | 0.103 | 0.084 | 0.125 | 0.401 |
| Deaths | Israel | 0.587 | 0.472 | 0.692 | 0.374 |
| Deaths | Kazakhstan | 0.398 | 0.332 | 0.476 | 0.362 |
| Deaths | Armenia | 0.279 | 0.232 | 0.332 | 0.360 |
| Deaths | Kyrgyzstan | 0.145 | 0.121 | 0.170 | 0.338 |
| Deaths | Uzbekistan | 0.208 | 0.177 | 0.245 | 0.328 |
| Incidence | United Arab Emirates | 5.152 | 3.978 | 6.630 | 0.515 |
| Incidence | Qatar | 4.417 | 3.521 | 5.522 | 0.453 |
| Incidence | Iran (Islamic Republic of) | 3.288 | 2.679 | 4.158 | 0.450 |
| Incidence | Kuwait | 3.542 | 2.914 | 4.437 | 0.430 |
| Incidence | Azerbaijan | 6.092 | 4.929 | 7.539 | 0.428 |
| Incidence | Oman | 3.728 | 3.079 | 4.664 | 0.425 |
| Incidence | Georgia | 5.992 | 4.943 | 7.440 | 0.417 |
| Incidence | Armenia | 6.117 | 5.055 | 7.580 | 0.413 |
| Incidence | Viet Nam | 0.669 | 0.560 | 0.836 | 0.413 |
| Incidence | Kyrgyzstan | 4.121 | 3.388 | 5.082 | 0.411 |
| Incidence | Democratic People's Republic of Korea | 1.132 | 0.930 | 1.392 | 0.408 |
| Incidence | Mongolia | 5.171 | 4.247 | 6.356 | 0.408 |
| Incidence | Philippines | 0.469 | 0.390 | 0.581 | 0.406 |
| Incidence | Bangladesh | 4.947 | 4.052 | 6.038 | 0.402 |
| Incidence | Cambodia | 0.620 | 0.520 | 0.767 | 0.398 |
| Incidence | Bhutan | 6.162 | 5.124 | 7.571 | 0.397 |
| Incidence | Kazakhstan | 5.360 | 4.400 | 6.487 | 0.389 |
| Incidence | Palestine | 2.698 | 2.277 | 3.325 | 0.388 |
| Incidence | Indonesia | 0.757 | 0.634 | 0.925 | 0.385 |
| Incidence | Maldives | 0.680 | 0.574 | 0.833 | 0.381 |
| Incidence | Uzbekistan | 5.002 | 4.182 | 6.083 | 0.380 |
| Incidence | Lao People's Democratic Republic | 0.606 | 0.513 | 0.743 | 0.379 |
| Incidence | Pakistan | 4.554 | 3.836 | 5.555 | 0.377 |
| Incidence | India | 5.853 | 4.923 | 7.131 | 0.377 |
| Incidence | Lebanon | 7.624 | 6.417 | 9.290 | 0.377 |
| Incidence | Syrian Arab Republic | 3.116 | 2.631 | 3.804 | 0.376 |
| Incidence | China | 1.781 | 1.501 | 2.166 | 0.374 |
| Incidence | Tajikistan | 3.880 | 3.238 | 4.684 | 0.373 |
| Incidence | Sri Lanka | 1.020 | 0.863 | 1.242 | 0.371 |
| Incidence | Nepal | 5.235 | 4.375 | 6.317 | 0.371 |
| Incidence | Bahrain | 2.770 | 2.316 | 3.343 | 0.371 |
| Incidence | Brunei Darussalam | 0.887 | 0.755 | 1.084 | 0.370 |
| Incidence | Jordan | 3.285 | 2.777 | 3.993 | 0.370 |
| Incidence | Japan | 2.487 | 2.114 | 3.031 | 0.369 |
| Incidence | Myanmar | 0.661 | 0.562 | 0.806 | 0.368 |
| Incidence | Thailand | 0.727 | 0.609 | 0.876 | 0.366 |
| Incidence | Turkmenistan | 5.143 | 4.297 | 6.174 | 0.365 |
| Incidence | Saudi Arabia | 2.649 | 2.261 | 3.218 | 0.361 |
| Incidence | Afghanistan | 1.940 | 1.659 | 2.359 | 0.361 |
| Incidence | Türkiye | 3.407 | 2.881 | 4.104 | 0.359 |
| Incidence | Timor-Leste | 0.524 | 0.445 | 0.632 | 0.358 |
| Incidence | Malaysia | 0.849 | 0.723 | 1.023 | 0.353 |
| Incidence | Singapore | 0.827 | 0.699 | 0.989 | 0.351 |
| Incidence | Israel | 13.158 | 11.232 | 15.815 | 0.348 |
| Incidence | Iraq | 2.499 | 2.141 | 3.007 | 0.346 |
| Incidence | Republic of Korea | 6.913 | 6.014 | 8.275 | 0.327 |
| Incidence | Cyprus | 11.984 | 10.212 | 13.984 | 0.315 |
| Prevalence | Oman | 40.585 | 33.534 | 49.937 | 0.404 |
| Prevalence | Afghanistan | 16.135 | 13.304 | 19.768 | 0.401 |
| Prevalence | Uzbekistan | 46.581 | 38.933 | 57.246 | 0.393 |
| Prevalence | United Arab Emirates | 65.111 | 53.594 | 78.874 | 0.388 |
| Prevalence | Iran (Islamic Republic of) | 40.682 | 34.190 | 49.701 | 0.381 |
| Prevalence | Democratic People's Republic of Korea | 7.496 | 6.307 | 9.101 | 0.373 |
| Prevalence | Syrian Arab Republic | 33.832 | 28.284 | 40.588 | 0.364 |
| Prevalence | Kyrgyzstan | 43.550 | 36.603 | 52.153 | 0.357 |
| Prevalence | Azerbaijan | 55.741 | 46.620 | 66.507 | 0.357 |
| Prevalence | Palestine | 28.593 | 23.918 | 34.114 | 0.357 |
| Prevalence | Turkmenistan | 42.897 | 35.676 | 50.972 | 0.357 |
| Prevalence | Lao People's Democratic Republic | 4.970 | 4.107 | 5.874 | 0.355 |
| Prevalence | Bahrain | 41.224 | 35.370 | 49.935 | 0.353 |
| Prevalence | Kuwait | 47.869 | 40.899 | 57.799 | 0.353 |
| Prevalence | Georgia | 69.220 | 57.730 | 82.110 | 0.352 |
| Prevalence | Brunei Darussalam | 12.570 | 10.541 | 14.958 | 0.351 |
| Prevalence | Qatar | 54.681 | 45.652 | 64.855 | 0.351 |
| Prevalence | Bangladesh | 50.509 | 42.084 | 59.780 | 0.350 |
| Prevalence | Mongolia | 40.647 | 34.252 | 48.420 | 0.349 |
| Prevalence | Pakistan | 30.245 | 25.412 | 35.925 | 0.348 |
| Prevalence | Bhutan | 49.173 | 41.231 | 58.307 | 0.347 |
| Prevalence | Cambodia | 5.013 | 4.160 | 5.884 | 0.344 |
| Prevalence | Nepal | 45.288 | 37.962 | 53.474 | 0.343 |
| Prevalence | Kazakhstan | 51.740 | 43.725 | 61.355 | 0.341 |
| Prevalence | Armenia | 68.042 | 57.397 | 80.493 | 0.339 |
| Prevalence | Maldives | 7.918 | 6.667 | 9.350 | 0.339 |
| Prevalence | Tajikistan | 34.292 | 28.632 | 40.220 | 0.338 |
| Prevalence | Jordan | 43.053 | 36.474 | 50.988 | 0.337 |
| Prevalence | Philippines | 4.343 | 3.674 | 5.136 | 0.336 |
| Prevalence | Myanmar | 5.338 | 4.525 | 6.307 | 0.334 |
| Prevalence | Japan | 44.817 | 38.514 | 53.461 | 0.334 |
| Prevalence | Sri Lanka | 10.331 | 8.822 | 12.265 | 0.333 |
| Prevalence | Indonesia | 6.148 | 5.265 | 7.314 | 0.333 |
| Prevalence | India | 48.466 | 41.270 | 57.373 | 0.332 |
| Prevalence | Saudi Arabia | 28.776 | 24.537 | 34.084 | 0.332 |
| Prevalence | Singapore | 10.835 | 9.240 | 12.779 | 0.327 |
| Prevalence | Timor-Leste | 4.295 | 3.690 | 5.078 | 0.323 |
| Prevalence | Lebanon | 110.336 | 94.035 | 129.512 | 0.322 |
| Prevalence | China | 12.295 | 10.571 | 14.497 | 0.319 |
| Prevalence | Thailand | 7.964 | 6.773 | 9.300 | 0.317 |
| Prevalence | Iraq | 24.913 | 21.167 | 29.069 | 0.317 |
| Prevalence | Republic of Korea | 83.523 | 72.128 | 98.426 | 0.315 |
| Prevalence | Türkiye | 44.168 | 38.082 | 51.945 | 0.314 |
| Prevalence | Malaysia | 8.993 | 7.683 | 10.470 | 0.310 |
| Prevalence | Israel | 141.601 | 121.267 | 164.833 | 0.308 |
| Prevalence | Viet Nam | 7.433 | 6.445 | 8.644 | 0.296 |
| Prevalence | Cyprus | 129.852 | 110.711 | 148.368 | 0.290 |

**S20 Table：ARIMA model selection, residual diagnostics, and out-of-sample validation for age-standardized IBD, UC, and CD burden measures in Asia.**

Panel A. Final-model selection and residual diagnostics based on the complete 1990–2023 series.

| Disease | Measure | Selected ARIMA model | AICc | Ljung-Box Q | df | P value |
| --- | --- | --- | --- | --- | --- | --- |
| IBD | ASIR | ARIMA(0,1,4) with drift | -216.669 | 3.268 | 6 | 0.774 |
| UC | ASIR | ARIMA(0,1,4) with drift | -242.894 | 3.766 | 6 | 0.708 |
| CD | ASIR | ARIMA(0,1,4) with drift | -285.860 | 3.000 | 6 | 0.809 |
| IBD | ASPR | ARIMA(2,1,0) with drift | -50.777 | 8.178 | 8 | 0.416 |
| UC | ASPR | ARIMA(2,1,0) with drift | -80.229 | 7.129 | 8 | 0.523 |
| CD | ASPR | ARIMA(2,1,1) with drift | -114.646 | 5.761 | 7 | 0.568 |
| IBD | ASMR | ARIMA(0,1,0) | -245.728 | 4.140 | 10 | 0.941 |
| UC | ASMR | ARIMA(0,1,2) | -252.408 | 5.708 | 8 | 0.680 |
| CD | ASMR | ARIMA(0,1,2) | -394.472 | 1.731 | 8 | 0.988 |

Panel B. Out-of-sample validation using 1990-2019 as the training period and 2020-2023 as the validation period

| Disease | Measure | Validation ARIMA model | MAE | RMSE | MAPE (%) | 95% PI coverage |
| --- | --- | --- | --- | --- | --- | --- |
| IBD | ASIR | ARIMA(2,1,1) with drift | 0.0114 | 0.0128 | 0.335 | 4/4 (100%) |
| UC | ASIR | ARIMA(2,1,1) with drift | 0.0069 | 0.0096 | 0.280 | 4/4 (100%) |
| CD | ASIR | ARIMA(3,1,0) | 0.0167 | 0.0192 | 1.777 | 4/4 (100%) |
| IBD | ASPR | ARIMA(3,2,0) | 1.6718 | 2.0822 | 5.796 | 0/4 (0%) |
| UC | ASPR | ARIMA(0,2,2) | 0.9461 | 1.1809 | 4.641 | 0/4 (0%) |
| CD | ASPR | ARIMA(2,2,0) | 0.4841 | 0.6018 | 5.721 | 1/4 (25%) |
| IBD | ASMR | ARIMA(0,1,3) | 0.0096 | 0.0105 | 3.259 | 2/4 (50%) |
| UC | ASMR | ARIMA(1,1,0) | 0.0075 | 0.0083 | 2.898 | 2/4 (50%) |
| CD | ASMR | ARIMA(1,1,0) | 0.0011 | 0.0014 | 3.519 | 2/4 (50%) |

Note: Separate non-seasonal ARIMA models were fitted for each disease-measure series. For the final projections, models were selected using the complete 1990-2023 series by minimizing the corrected Akaike information criterion (AICc) with stepwise = FALSE and approximation = FALSE. Residual autocorrelation was assessed using the Ljung-Box test at lag 10. All Ljung-Box P values were greater than 0.05, indicating no evidence of remaining residual autocorrelation. For out-of-sample validation, models were selected and fitted using the 1990-2019 training series and evaluated against the four observed estimates for 2020-2023. Prediction-interval coverage is presented as the number and percentage of observed validation values falling within the corresponding 95% ARIMA prediction intervals. Because the validation period included only four observations, coverage could change only in increments of 25 percentage points. AICc, corrected Akaike information criterion; ARIMA, autoregressive integrated moving average; IBD, inflammatory bowel disease; UC, ulcerative colitis; CD, Crohn's disease; ASIR, age-standardized incidence rate; ASPR, age-standardized prevalence rate; ASMR, age-standardized mortality rate; MAE, mean absolute error; RMSE, root mean squared error; MAPE, mean absolute percentage error; PI, prediction interval.

**S21 Table. Sex-specific age-standardized DALY and mortality rates of IBD, UC, and CD across Asia and Asian subregions in 2023.**

| Region | Subtype | Male ASDR (95% UI) | Female ASDR (95% UI) | Male ASMR (95% UI) | Female ASMR (95% UI) |
| --- | --- | --- | --- | --- | --- |
| Asia | CD | 2.541 (1.550-4.043) | 2.613 (1.687-3.935) | 0.036 (0.015-0.073) | 0.030 (0.011-0.064) |
| Asia | IBD | 12.843 (8.530-18.975) | 11.263 (7.477-15.985) | 0.314 (0.178-0.522) | 0.276 (0.140-0.474) |
| Asia | UC | 10.301 (6.575-15.654) | 8.650 (5.535-12.777) | 0.278 (0.157-0.461) | 0.246 (0.124-0.427) |
| Central Asia | CD | 4.905 (3.385-6.839) | 4.685 (3.266-6.316) | 0.082 (0.052-0.124) | 0.049 (0.030-0.074) |
| Central Asia | IBD | 18.010 (14.129-23.092) | 16.653 (12.444-21.502) | 0.296 (0.217-0.398) | 0.226 (0.167-0.308) |
| Central Asia | UC | 13.105 (9.812-17.253) | 11.967 (8.630-15.855) | 0.214 (0.145-0.303) | 0.178 (0.124-0.252) |
| East Asia | CD | 1.593 (1.123-2.194) | 1.305 (0.924-1.824) | 0.027 (0.014-0.044) | 0.015 (0.007-0.026) |
| East Asia | IBD | 10.379 (7.556-14.449) | 8.372 (5.733-11.714) | 0.376 (0.249-0.554) | 0.332 (0.177-0.529) |
| East Asia | UC | 8.786 (6.219-12.404) | 7.067 (4.499-10.306) | 0.349 (0.233-0.518) | 0.318 (0.169-0.512) |
| South Asia | CD | 3.280 (1.912-5.348) | 3.998 (2.553-6.002) | 0.038 (0.013-0.088) | 0.042 (0.014-0.095) |
| South Asia | IBD | 15.716 (10.128-23.244) | 14.821 (9.801-20.559) | 0.290 (0.138-0.530) | 0.255 (0.123-0.474) |
| South Asia | UC | 12.436 (7.636-18.991) | 10.823 (7.088-15.721) | 0.252 (0.118-0.452) | 0.213 (0.102-0.400) |
| Southeast Asia | CD | 1.307 (0.590-2.737) | 0.974 (0.493-1.897) | 0.029 (0.009-0.067) | 0.019 (0.006-0.045) |
| Southeast Asia | IBD | 9.179 (4.931-16.363) | 6.270 (3.521-10.989) | 0.258 (0.129-0.478) | 0.208 (0.097-0.378) |
| Southeast Asia | UC | 7.871 (4.103-14.488) | 5.296 (2.797-9.304) | 0.229 (0.114-0.439) | 0.189 (0.085-0.345) |
| West Asia | CD | 4.690 (2.957-7.263) | 3.900 (2.506-5.951) | 0.073 (0.035-0.135) | 0.054 (0.023-0.106) |
| West Asia | IBD | 14.302 (9.918-20.463) | 13.909 (9.616-19.424) | 0.267 (0.153-0.446) | 0.282 (0.151-0.454) |
| West Asia | UC | 9.612 (6.430-14.191) | 10.009 (6.580-14.289) | 0.194 (0.109-0.330) | 0.228 (0.116-0.380) |
| Region | Subtype | Male ASDR (95% UI) | Female ASDR (95% UI) | Male ASMR (95% UI) | Female ASMR (95% UI) |

ASDR, age-standardized DALY rate. ASMR, age-standardized mortality rate.

**S22 Table. Comparison of temporal trends in age-standardized burden measures between ulcerative colitis and Crohn’s disease in Asia and its subregions, 1990–2023.**

| Region | Measure | UC EAPC (95% CI) | CD EAPC (95% CI) | P for temporal-trend difference | FDR-adjusted P |
| --- | --- | --- | --- | --- | --- |
| Asia | ASIR | 2.00 (1.85 to 2.15) | 1.83 (1.66 to 2.00) | 0.137 | 0.221 |
| Asia | ASPR | 1.99 (1.82 to 2.16) | 1.84 (1.63 to 2.05) | 0.270 | 0.341 |
| Asia | ASDR | -0.38 (-0.46 to -0.31) | 0.63 (0.55 to 0.71) | <0.001 | <0.001 |
| Asia | ASMR | -0.17 (-0.31 to -0.03) | 0.20 (0.11 to 0.29) | <0.001 | <0.001 |
| East Asia | ASIR | 3.08 (2.53 to 3.63) | 2.29 (1.82 to 2.76) | 0.030 | 0.059 |
| East Asia | ASPR | 2.61 (2.03 to 3.19) | 2.08 (1.52 to 2.64) | 0.188 | 0.281 |
| East Asia | ASDR | -1.10 (-1.29 to -0.92) | 0.40 (0.11 to 0.69) | <0.001 | <0.001 |
| East Asia | ASMR | -0.22 (-0.51 to 0.08) | -0.11 (-0.36 to 0.14) | 0.571 | 0.595 |
| Southeast Asia | ASIR | 1.23 (1.19 to 1.26) | 0.91 (0.84 to 0.98) | <0.001 | <0.001 |
| Southeast Asia | ASPR | 1.28 (1.24 to 1.33) | 0.91 (0.86 to 0.96) | <0.001 | <0.001 |
| Southeast Asia | ASDR | -0.14 (-0.27 to -0.01) | 1.06 (1.00 to 1.13) | <0.001 | <0.001 |
| Southeast Asia | ASMR | 0.33 (0.20 to 0.46) | 1.70 (1.62 to 1.79) | <0.001 | <0.001 |
| South Asia | ASIR | 1.39 (1.33 to 1.45) | 1.47 (1.35 to 1.58) | 0.249 | 0.333 |
| South Asia | ASPR | 1.50 (1.42 to 1.58) | 1.55 (1.41 to 1.68) | 0.543 | 0.592 |
| South Asia | ASDR | 0.01 (-0.07 to 0.08) | 0.50 (0.40 to 0.59) | <0.001 | <0.001 |
| South Asia | ASMR | -0.09 (-0.21 to 0.03) | -0.05 (-0.14 to 0.05) | 0.540 | 0.592 |
| Central Asia | ASIR | 1.23 (1.15 to 1.31) | 1.04 (0.95 to 1.14) | 0.003 | 0.006 |
| Central Asia | ASPR | 1.11 (1.04 to 1.18) | 0.94 (0.87 to 1.02) | 0.001 | 0.003 |
| Central Asia | ASDR | -0.32 (-0.56 to -0.08) | 0.01 (-0.20 to 0.22) | 0.037 | 0.068 |
| Central Asia | ASMR | -0.61 (-0.94 to -0.28) | -0.36 (-0.81 to 0.09) | 0.364 | 0.436 |
| West Asia | ASIR | 1.35 (1.26 to 1.44) | 1.23 (1.05 to 1.41) | 0.250 | 0.333 |
| West Asia | ASPR | 1.60 (1.48 to 1.73) | 1.57 (1.30 to 1.84) | 0.842 | 0.842 |
| West Asia | ASDR | 0.02 (-0.08 to 0.11) | -0.07 (-0.15 to 0.01) | 0.138 | 0.221 |
| West Asia | ASMR | 0.21 (0.00 to 0.42) | -0.54 (-0.84 to -0.24) | <0.001 | <0.001 |

**S23 Table. Temporal changes in UC-to-CD ratios for age-standardized incidence, prevalence, DALY, and mortality rates in Asia and its subregions, 1990–2023.**

| Region | Measure | UC:CD ratio, 1990 | UC:CD ratio, 2023 | Relative change (%) | EAPC (95% CI) | P value | R squared | Temporal pattern |
| --- | --- | --- | --- | --- | --- | --- | --- | --- |
| Asia | DALYs | 4.949 | 3.684 | -25.56 | -1.00 (-1.10 to -0.91) | 6.32E-20 | 0.929 | Decreasing |
| Asia | Deaths | 8.39 | 7.946 | -5.29 | -0.37 (-0.49 to -0.25) | 0.00000133 | 0.524 | Decreasing |
| Asia | Incidence | 2.543 | 2.61 | 2.62 | 0.17 (0.12 to 0.21) | 3.06E-08 | 0.622 | Increasing |
| Asia | Prevalence | 2.371 | 2.409 | 1.59 | 0.15 (0.08 to 0.21) | 0.000045 | 0.41 | Increasing |
| Central Asia | DALYs | 2.522 | 2.511 | -0.42 | 0.14 (0.02 to 0.26) | 0.0335 | 0.134 | Increasing |
| Central Asia | Deaths | 2.506 | 2.832 | 13.03 | 0.64 (0.29 to 0.99) | 0.0011 | 0.287 | Increasing |
| Central Asia | Incidence | 2.284 | 2.45 | 7.25 | 0.18 (0.14 to 0.21) | 3.61E-12 | 0.784 | Increasing |
| Central Asia | Prevalence | 2.142 | 2.272 | 6.08 | 0.17 (0.15 to 0.18) | 4.4E-21 | 0.94 | Increasing |
| East Asia | DALYs | 8.442 | 5.471 | -35.19 | -1.50 (-1.74 to -1.26) | 1.26E-13 | 0.824 | Decreasing |
| East Asia | Deaths | 15.238 | 16.136 | 5.89 | -0.11 (-0.19 to -0.02) | 0.0201 | 0.158 | Decreasing |
| East Asia | Incidence | 2.076 | 2.097 | 1.01 | 0.77 (0.48 to 1.06) | 0.00000967 | 0.463 | Increasing |
| East Asia | Prevalence | 2.206 | 2.105 | -4.59 | 0.52 (0.23 to 0.80) | 0.00109 | 0.287 | Increasing |
| South Asia | DALYs | 3.657 | 3.206 | -12.32 | -0.49 (-0.56 to -0.41) | 1.26E-13 | 0.824 | Decreasing |
| South Asia | Deaths | 5.882 | 5.863 | -0.33 | -0.05 (-0.13 to 0.04) | 0.299 | 0.034 | Stable or uncertain |
| South Asia | Incidence | 2.745 | 2.814 | 2.51 | -0.07 (-0.16 to 0.02) | 0.123 | 0.073 | Stable or uncertain |
| South Asia | Prevalence | 2.481 | 2.55 | 2.78 | -0.05 (-0.14 to 0.05) | 0.347 | 0.028 | Stable or uncertain |
| Southeast Asia | DALYs | 8.014 | 5.769 | -28.01 | -1.19 (-1.32 to -1.07) | 5.85E-19 | 0.918 | Decreasing |
| Southeast Asia | Deaths | 12.404 | 8.714 | -29.75 | -1.36 (-1.50 to -1.21) | 1.14E-18 | 0.915 | Decreasing |
| Southeast Asia | Incidence | 2.18 | 2.416 | 10.81 | 0.32 (0.25 to 0.38) | 1.17E-10 | 0.732 | Increasing |
| Southeast Asia | Prevalence | 2.017 | 2.263 | 12.21 | 0.37 (0.32 to 0.41) | 6.68E-16 | 0.873 | Increasing |
| West Asia | DALYs | 2.369 | 2.283 | -3.63 | -0.07 (-0.14 to -0.00) | 0.0574 | 0.108 | Stable or uncertain |
| West Asia | Deaths | 3.025 | 3.357 | 10.98 | 0.42 (0.37 to 0.47) | 2.15E-18 | 0.911 | Increasing |
| West Asia | Incidence | 2.771 | 2.666 | -3.78 | 0.09 (-0.06 to 0.24) | 0.264 | 0.039 | Stable or uncertain |
| West Asia | Prevalence | 2.432 | 2.316 | -4.78 | 0.00 (-0.20 to 0.20) | 0.987 | 0 | Stable or uncertain |

**S24 Table. Sex-specific age-standardized incidence, prevalence, DALY, and mortality rates for IBD, UC, and CD across Asia and its subregions, 2023.**

| Region | Sub-type | Measure | Male rate (95% UI) | Female rate (95% UI) | Male-to-female ratio | N_countries |
| --- | --- | --- | --- | --- | --- | --- |
| Asia | IBD | DALYs | 12.843 (8.530–18.975) | 11.263 (7.477–15.985) | 1.14 | 47 |
| Asia | IBD | Deaths | 0.314 (0.178–0.522) | 0.276 (0.140–0.474) | 1.136 | 47 |
| Asia | IBD | Incidence | 3.462 (2.890–4.244) | 3.373 (2.841–4.113) | 1.026 | 47 |
| Asia | IBD | Prevalence | 29.190 (24.865–34.657) | 30.084 (25.543–35.911) | 0.97 | 47 |
| Asia | UC | DALYs | 10.301 (6.575–15.654) | 8.650 (5.535–12.777) | 1.191 | 47 |
| Asia | UC | Deaths | 0.278 (0.157–0.461) | 0.246 (0.124–0.427) | 1.127 | 47 |
| Asia | UC | Incidence | 2.583 (2.027–3.374) | 2.356 (1.859–3.091) | 1.096 | 47 |
| Asia | UC | Prevalence | 21.219 (16.873–26.735) | 20.643 (16.457–25.829) | 1.028 | 47 |
| Asia | CD | DALYs | 2.541 (1.550–4.043) | 2.613 (1.687–3.935) | 0.973 | 47 |
| Asia | CD | Deaths | 0.036 (0.015–0.073) | 0.030 (0.011–0.064) | 1.203 | 47 |
| Asia | CD | Incidence | 0.879 (0.727–1.109) | 1.017 (0.838–1.281) | 0.865 | 47 |
| Asia | CD | Prevalence | 7.971 (6.365–9.955) | 9.440 (7.412–11.943) | 0.844 | 47 |
| East Asia | IBD | DALYs | 10.379 (7.556–14.449) | 8.372 (5.733–11.714) | 1.24 | 5 |
| East Asia | IBD | Deaths | 0.376 (0.249–0.554) | 0.332 (0.177–0.529) | 1.131 | 5 |
| East Asia | IBD | Incidence | 2.002 (1.681–2.440) | 1.985 (1.683–2.416) | 1.009 | 5 |
| East Asia | IBD | Prevalence | 16.624 (14.237–19.587) | 17.398 (14.973–20.597) | 0.956 | 5 |
| East Asia | UC | DALYs | 8.786 (6.219–12.404) | 7.067 (4.499–10.306) | 1.243 | 5 |
| East Asia | UC | Deaths | 0.349 (0.233–0.518) | 0.318 (0.169–0.512) | 1.099 | 5 |
| East Asia | UC | Incidence | 1.399 (1.096–1.827) | 1.299 (1.025–1.696) | 1.076 | 5 |
| East Asia | UC | Prevalence | 11.515 (9.310–14.349) | 11.541 (9.341–14.502) | 0.998 | 5 |
| East Asia | CD | DALYs | 1.593 (1.123–2.194) | 1.305 (0.924–1.824) | 1.22 | 5 |
| East Asia | CD | Deaths | 0.027 (0.014–0.044) | 0.015 (0.007–0.026) | 1.828 | 5 |
| East Asia | CD | Incidence | 0.604 (0.495–0.776) | 0.685 (0.563–0.877) | 0.881 | 5 |
| East Asia | CD | Prevalence | 5.109 (4.088–6.434) | 5.858 (4.724–7.241) | 0.872 | 5 |
| Southeast Asia | IBD | DALYs | 9.177 (4.931–16.359) | 6.268 (3.520–10.986) | 1.464 | 12 |
| Southeast Asia | IBD | Deaths | 0.258 (0.129–0.478) | 0.208 (0.097–0.378) | 1.241 | 12 |
| Southeast Asia | IBD | Incidence | 0.691 (0.574–0.852) | 0.679 (0.574–0.827) | 1.018 | 12 |
| Southeast Asia | IBD | Prevalence | 6.013 (5.112–7.151) | 6.559 (5.557–7.737) | 0.917 | 12 |
| Southeast Asia | UC | DALYs | 7.869 (4.102–14.483) | 5.294 (2.797–9.301) | 1.486 | 12 |
| Southeast Asia | UC | Deaths | 0.229 (0.113–0.439) | 0.189 (0.085–0.345) | 1.213 | 12 |
| Southeast Asia | UC | Incidence | 0.507 (0.401–0.669) | 0.462 (0.364–0.604) | 1.099 | 12 |
| Southeast Asia | UC | Prevalence | 4.309 (3.405–5.488) | 4.409 (3.533–5.563) | 0.977 | 12 |
| Southeast Asia | CD | DALYs | 1.308 (0.591–2.738) | 0.974 (0.493–1.897) | 1.344 | 12 |
| Southeast Asia | CD | Deaths | 0.029 (0.009–0.067) | 0.019 (0.006–0.045) | 1.512 | 12 |
| Southeast Asia | CD | Incidence | 0.184 (0.147–0.239) | 0.218 (0.175–0.283) | 0.845 | 12 |
| Southeast Asia | CD | Prevalence | 1.704 (1.294–2.195) | 2.150 (1.652–2.771) | 0.792 | 12 |
| South Asia | IBD | DALYs | 15.719 (10.130–23.248) | 14.824 (9.803–20.562) | 1.06 | 7 |
| South Asia | IBD | Deaths | 0.290 (0.138–0.530) | 0.255 (0.123–0.474) | 1.139 | 7 |
| South Asia | IBD | Incidence | 5.534 (4.620–6.781) | 5.406 (4.553–6.588) | 1.024 | 7 |
| South Asia | IBD | Prevalence | 44.575 (38.012–52.916) | 46.008 (39.053–54.853) | 0.969 | 7 |
| South Asia | UC | DALYs | 12.439 (7.638–18.994) | 10.825 (7.089–15.724) | 1.149 | 7 |
| South Asia | UC | Deaths | 0.252 (0.118–0.452) | 0.213 (0.102–0.400) | 1.186 | 7 |
| South Asia | UC | Incidence | 4.230 (3.325–5.522) | 3.836 (3.029–5.047) | 1.103 | 7 |
| South Asia | UC | Prevalence | 33.225 (26.346–41.926) | 31.794 (25.253–39.553) | 1.045 | 7 |
| South Asia | CD | DALYs | 3.280 (1.912–5.348) | 3.999 (2.553–6.003) | 0.82 | 7 |
| South Asia | CD | Deaths | 0.038 (0.013–0.088) | 0.042 (0.014–0.095) | 0.904 | 7 |
| South Asia | CD | Incidence | 1.304 (1.087–1.626) | 1.570 (1.301–1.961) | 0.83 | 7 |
| South Asia | CD | Prevalence | 11.350 (9.123–14.023) | 14.213 (11.115–18.046) | 0.799 | 7 |
| Central Asia | IBD | DALYs | 17.762 (13.771–23.014) | 16.257 (12.102–21.135) | 1.093 | 8 |
| Central Asia | IBD | Deaths | 0.288 (0.207–0.395) | 0.212 (0.155–0.290) | 1.359 | 8 |
| Central Asia | IBD | Incidence | 5.002 (4.084–6.247) | 5.169 (4.262–6.291) | 0.968 | 8 |
| Central Asia | IBD | Prevalence | 45.451 (37.872–55.106) | 51.279 (42.277–63.226) | 0.886 | 8 |
| Central Asia | UC | DALYs | 12.698 (9.390–16.903) | 11.631 (8.333–15.469) | 1.092 | 8 |
| Central Asia | UC | Deaths | 0.202 (0.134–0.291) | 0.167 (0.115–0.239) | 1.21 | 8 |
| Central Asia | UC | Incidence | 3.687 (2.849–4.863) | 3.538 (2.717–4.575) | 1.042 | 8 |
| Central Asia | UC | Prevalence | 32.618 (25.435–42.398) | 34.564 (26.888–45.235) | 0.944 | 8 |
| Central Asia | CD | DALYs | 5.063 (3.436–7.165) | 4.625 (3.182–6.287) | 1.095 | 8 |
| Central Asia | CD | Deaths | 0.086 (0.053–0.135) | 0.045 (0.028–0.068) | 1.913 | 8 |
| Central Asia | CD | Incidence | 1.315 (1.059–1.681) | 1.631 (1.316–2.085) | 0.807 | 8 |
| Central Asia | CD | Prevalence | 12.832 (10.099–16.286) | 16.716 (12.788–21.749) | 0.768 | 8 |
| West Asia | IBD | DALYs | 14.192 (9.814–20.357) | 13.878 (9.564–19.422) | 1.023 | 15 |
| West Asia | IBD | Deaths | 0.268 (0.152–0.449) | 0.290 (0.154–0.467) | 0.923 | 15 |
| West Asia | IBD | Incidence | 3.736 (3.102–4.623) | 3.483 (2.899–4.298) | 1.073 | 15 |
| West Asia | IBD | Prevalence | 43.390 (36.605–51.936) | 43.490 (36.370–52.798) | 0.998 | 15 |
| West Asia | UC | DALYs | 9.556 (6.384–14.140) | 10.004 (6.558–14.322) | 0.955 | 15 |
| West Asia | UC | Deaths | 0.197 (0.110–0.334) | 0.234 (0.118–0.391) | 0.839 | 15 |
| West Asia | UC | Incidence | 2.690 (2.103–3.516) | 2.563 (2.026–3.338) | 1.05 | 15 |
| West Asia | UC | Prevalence | 29.812 (23.580–37.486) | 30.927 (24.671–39.050) | 0.964 | 15 |
| West Asia | CD | DALYs | 4.636 (2.922–7.194) | 3.874 (2.490–5.940) | 1.197 | 15 |
| West Asia | CD | Deaths | 0.071 (0.034–0.132) | 0.056 (0.024–0.110) | 1.277 | 15 |
| West Asia | CD | Incidence | 1.046 (0.852–1.342) | 0.919 (0.740–1.164) | 1.138 | 15 |
| West Asia | CD | Prevalence | 13.578 (10.627–17.368) | 12.563 (9.685–16.073) | 1.081 | 15 |
