# Supplementary material for: The burden of inflammatory bowel disease in Asia (1990–2023) and projections to 2050: A comprehensive analysis of the 2023 global burden of disease study
Source: PLoS One. 2026 Oct 8;21(10):e0359856. doi: 10.1371/journal.pone.0359856 (PMC13649031; doi:10.1371/journal.pone.0359856)
Supplement: Supplementary file 2 [file pone.0359856.s004.docx]

**Response to Reviewers**

Manuscript ID: PONE-D-26-15368
Title: *The Burden of Inflammatory Bowel Disease in Asia (1990-2023) and Projections to 2050: A Comprehensive Analysis of the 2023 Global Burden of Disease Study*

Dear Dr. Paulraj and Reviewers,

Thank you for your careful evaluation of our manuscript and for the constructive comments and suggestions. We have revised the manuscript extensively in response to the editor's and reviewers' recommendations. We believe these revisions have substantially improved the clarity, rigor, and overall quality of the manuscript.

For ease of review, we provide a point-by-point response below. Reviewer comments are reproduced in bold, followed by our responses. All changes have been incorporated into the revised manuscript and highlighted in the tracked-changes version.

**Response to Journal Requirements**

Comment 1: **Please ensure that your manuscript meets PLOS ONE's style requirements, including those for file naming.**

Response: Thank you for this reminder. We have carefully revised the manuscript and submission files to conform to the PLOS ONE formatting and file-naming requirements.

Comment 2: **Please note that PLOS One has specific guidelines on code sharing for submissions in which author-generated code underpins the findings in the manuscript. In these cases, we expect all author-generated code to be made available without restrictions upon publication of the work.**

Response: Thank you for this comment. We have uploaded a compressed file with the revised submission containing the author-generated code and the data required for reproducing the analyses in this study. The file includes the scripts used for data processing, statistical analysis, forecasting, and figure generation. We have also clarified in the Data Availability Statement that these materials are available as part of the submission.

Comment 3: **Please note that funding information should not appear in any section or other areas of your manuscript. We will only publish funding information present in the Funding Statement section of the online submission form. Please remove any funding-related text from the manuscript.**

Response: We have removed all funding-related text from the manuscript body. Funding information is now provided only in the appropriate section of the online submission form, in accordance with journal requirements.

Comment 4: **The grant information provided in the "Funding Information" and "Financial Disclosure" sections does not match.**

Response: Thank you for noting this inconsistency. We have corrected the grant information during resubmission and ensured that the funding details provided in the “Funding Information” section are now accurate and consistent with the “Financial Disclosure” section.

Comment 5: **Please state what role the funders took in the study.**

Response: We have clarified the role of the funders in the cover letter and request that the submission record be updated accordingly. The revised statement is:

"The funders had no role in study design, data collection and analysis, decision to publish, or preparation of the manuscript."

Comment 6: **Please clarify whether part of the manuscript was previously presented or published elsewhere, and if so, whether that constituted prior peer-reviewed publication.**

Response: We clarify that this work has not been previously peer-reviewed or formally published elsewhere. We have included this clarification in the cover letter.

Comment 7: **Figure 3, Appendix Figure 1, and Appendix Figure 2 may contain copyrighted map images.**

Response: Thank you for raising this issue. Figure 3, Appendix Figure 1, and Appendix Figure 2 were generated by the authors using original R code rather than reproduced from previously published images. The map boundary data were obtained from Natural Earth (public domain), accessed via the rnaturalearth and rnaturalearthdata packages. We have updated the figure captions accordingly. In particular, the revised captions now state: “Base map data were obtained from Natural Earth (public domain). The figure was generated by the authors using original R code.”

Comment 8: **Please include your tables as part of the main manuscript and remove the individual files.**

Response: Thank you for this comment. In the revised submission, all tables belonging to the main text have been incorporated into the manuscript itself, and the corresponding separate table files have been removed. Supplementary tables have been retained and uploaded separately as supporting information files in accordance with the journal’s requirements.

Comment 9: **Please include captions for your Supporting Information files at the end of the manuscript and update in-text citations accordingly.**

Response: Thank you for this comment. We have added captions for all Supporting Information files at the end of the revised manuscript and updated the corresponding in-text citations to ensure consistency with the uploaded supplementary materials.

Comment 10: **The submitted data files contain potentially identifying information and must be anonymized before resubmission.**

Response: Thank you for raising this concern. We would like to clarify that the submitted data sheet files do not contain individual-level participant data. They consist solely of aggregated estimates obtained from the publicly available GBD database and do not include any personally identifiable information. We have re-checked all submitted data sheet files, confirmed that no identifying data are present, and re-uploaded them in a clean format with their public GBD source clearly indicated.

Comment11: **If reviewers recommend citing previously published work, please evaluate whether those works are relevant.**

Response: Thank you for this clarification. As the reviewers did not recommend any specific previously published works for citation, no changes were required in response to this point.

**Response to Additional Editor Comments**

Overall editorial assessment

Comment 1: **Please carefully address Reviewer 3's methodological concerns regarding the limitations of GBD-based estimates, justification and validation of the ARIMA forecasting model, interpretation of temporal trends, and the discussion of SDI-related findings. In particular, the Discussion should more clearly acknowledge the uncertainty associated with model-derived estimates and avoid causal interpretations of correlation analyses.**

Response: Thank you for the careful and constructive editorial assessment. In response to Reviewer 3’s methodological concerns, we have substantially revised the manuscript to more explicitly acknowledge the limitations and uncertainty of GBD-based estimates, particularly in settings with sparse primary epidemiological data. We also conducted additional sensitivity analyses to assess the robustness of key findings, including exclusion analyses for high-uncertainty countries and re-analysis of temporal trends using the lower and upper bounds of the 95% uncertainty intervals. The Discussion and Limitations sections have been revised accordingly to emphasize that model-derived estimates should be interpreted with caution.

We also revised the forecasting component to clarify that the ARIMA-based results represent trend-based projections rather than mechanistic forecasts of future epidemiologic change. In addition, we performed out-of-sample validation using data from 1990-2019 as the training period and 2020-2023 as the validation period, and summarized the validation results in the revised manuscript and supplementary materials. The interpretation of temporal trends has also been revised to acknowledge that the observed increases may reflect not only true epidemiological change, but also improved diagnosis, increased endoscopic access, greater clinician awareness, and strengthening of health information systems over time.

For the SDI-related findings, we have avoided causal language and now describe these results as cross-sectional associations rather than evidence that higher SDI leads to higher IBD burden. We further strengthened the analysis by repeating the SDI-related analyses using Spearman correlation and after excluding high-uncertainty countries. These revisions are now reflected in the Methods, Results, Discussion, and Limitations sections.

Comment 2: **In addition, please strengthen the discussion of regional differences in IBD burden, healthcare infrastructure, diagnostic capacity, and access to advanced therapies across Asia. The age-, sex-, and disease subtype-specific findings should also be further interpreted.**

Response: We expanded the Discussion of regional differences in IBD burden across Asia by incorporating consideration of healthcare infrastructure, diagnostic capacity, access to biologic therapies and specialist care, healthcare system differences, the diagnostic challenge of distinguishing Crohn’s disease from intestinal tuberculosis in endemic settings, and the possible impact of the COVID-19 period on IBD care. We also further interpreted the age-, sex-, and disease subtype-specific findings, including additional stratified analyses of sex differences by region and subtype.

Comment 3: **Reviewer 1's suggestions regarding presentation of results, comparison with global trends, and discussion of factors such as smoking and diagnostic awareness are encouraged and should be considered where appropriate.**

Response: In response to Reviewer 1’s suggestions, we improved the presentation of the Results section by moving detailed numerical information into tables, added discussion placing the Asian trends in a broader global context, and expanded the Discussion of potential contributing factors such as smoking patterns and changes in diagnostic awareness.

Comment 4: **Finally, please improve the overall presentation of the manuscript, including language editing, figure quality, and readability of the Results section.**

Response: we revised the manuscript for language clarity, improved figure resolution and quality to meet journal requirements, and enhanced the overall readability and presentation of the revised manuscript.

**Response to Reviewer #1**

We sincerely thank Reviewer #1 for the positive evaluation of our work and for the valuable suggestions to improve the presentation and contextual interpretation of the manuscript.

Comment 1: **The results section is very difficult to read currently. Much of the numerical data could be moved to tables.**

Response: Thank you for this helpful suggestion. We agree that the original Results section contained too much dense numerical information in the main text. In the revised manuscript, we have reorganized the Results section for clarity and moved selected detailed numerical results into tables, while retaining the main messages in the narrative text. We believe this substantially improves readability.

Comment 2: **Consider including published trend data from other parts of the world to assess whether the changes are worldwide.**

Response: Thank you for this valuable suggestion. We agree that placing the Asian trends in a broader international context strengthens the manuscript. Although the present study did not generate de novo estimates for other world regions, we have revised the Discussion to compare our findings with recent published global and transregional epidemiological studies. These additions clarify that the increasing burden observed in Asia is broadly consistent with a wider worldwide shift in IBD epidemiology, while also highlighting important regional differences.

Comment 3: **The authors could speculate more on whether the trends reflect true increases in disease rates or increased diagnosis.**

Response: Thank you for this valuable comment. We agree that the increasing trends observed in this study may reflect both true epidemiological change and improved diagnosis and reporting over time. In the revised Discussion, we have added text to explicitly acknowledge the potential contribution of improved case ascertainment, including expanded diagnostic capacity, greater clinician awareness, and better surveillance systems. We have also added relevant references and revised the wording to avoid implying that all observed increases necessarily represent true changes in underlying disease occurrence.

Comment 4: **What about population cigarette smoking rates over this time, a major association with IBD?**

Response: Thank you for raising this important point. We agree that cigarette smoking is a relevant environmental factor in IBD and may have contributed to the temporal and regional variation observed in this study. In the revised Discussion, we have added text acknowledging that changes in smoking patterns over time, as well as differences across countries and between sexes, may partly influence the burden of IBD, particularly for Crohn's disease. At the same time, we clarify that the present study was not designed to directly quantify the contribution of smoking, and therefore this interpretation remains cautious.

**Response to Reviewer #2**

We thank Reviewer #2 for the thoughtful comments regarding broader environmental and public health considerations.

Comment 1: **Future studies should more extensively consider the contribution of diet, nutrition, and other environmental or social factors, as well as public health approaches to alter disease onset and course.**

Response: Thank you for this thoughtful and valuable comment. We agree that diet, nutrition, environmental exposures, and broader social determinants are important contributors to the development and progression of IBD. These factors were beyond the scope of the present GBD-based analysis, which was designed to describe temporal, regional, and demographic patterns of disease burden in Asia. In response to your suggestion, we have expanded the Discussion to acknowledge the likely contribution of these factors and to emphasize the importance of future studies that integrate environmental, nutritional, and social determinants. We have also added brief public health implications regarding early detection, equitable access to care, and long-term disease management.

**Response to Reviewer #3**

We sincerely thank Reviewer #3 for the detailed and insightful comments. These suggestions have been highly valuable in strengthening the methodological interpretation and clinical relevance of our study. We address each point below.

Comment 1: **The study relies entirely on GBD 2023 modeled estimates without adequately acknowledging the limitations of this approach, especially in Asian countries with sparse primary data.**

Response: Thank you for this important and insightful comment. We would first like to clarify that our study did not rely solely on data from 2023. The temporal analyses were based on GBD 2023 estimates for the full period from 1990 to 2023, whereas some figures were restricted to 2023 in order to present the most recent cross-sectional geographic and demographic patterns. We agree, however, that this distinction was not sufficiently clear in the original manuscript.

More importantly, we agree that the limitations of relying on GBD-based estimates, particularly for countries with sparse primary epidemiological data and limited IBD surveillance infrastructure, should be more explicitly acknowledged. In the revised manuscript, we have expanded the Methods, Discussion, and Limitations sections to clarify that GBD estimates are partly model-derived and that uncertainty may be greater in low- and middle-income settings where primary data are limited. We now explicitly note that estimates for some countries should be interpreted with caution, especially where disease surveillance, registry coverage, and diagnostic capacity are less developed.

To address the concern regarding robustness, we conducted several sensitivity analyses using the publicly available GBD output data. First, we identified countries with high relative uncertainty in the 2023 estimates and repeated selected analyses after excluding these countries. We also performed an additional exclusion analysis for reviewer-highlighted countries with potentially limited data infrastructure, including Afghanistan, Myanmar, and Lao People's Democratic Republic. Second, temporal trend analyses were repeated using the point estimates as well as the lower and upper bounds of the 95% uncertainty intervals. Third, SDI-related associations were re-examined using both Pearson and Spearman correlation coefficients and after excluding high-uncertainty countries. These analyses showed that the increasing trends in incidence and prevalence were generally robust, whereas findings related to DALY, mortality, and some SDI-related associations were less stable and are now interpreted more cautiously in the revised manuscript.

We agree that a more detailed evaluation of the internal GBD modeling process for data-sparse settings would be valuable. However, because the present study used publicly available GBD output data rather than the full underlying modeling framework, our analyses were limited to output-level sensitivity assessments. We have now clarified this point in the revised manuscript.

Comment 2: **The ARIMA model assumes future trends follow historical patterns, which may not adequately capture epidemiologic transition. The manuscript also lacks validation and alternative scenarios.**

Response: Thank you for this important and constructive comment. We agree that ARIMA models have inherent limitations for forecasting IBD burden because they assume that future trends will largely follow historical patterns and therefore may not fully capture epidemiologic transitions, changes in diagnostic capacity, improvements in treatment access, or other structural shifts over time. In the revised manuscript, we have clarified that the ARIMA-based estimates should be interpreted as trend-based projections derived from historical data rather than mechanistic forecasts of future disease occurrence.

To strengthen this part of the analysis, we performed an out-of-sample validation by fitting the ARIMA models to data from 1990 to 2019 and comparing the predicted values with the observed estimates for 2020-2023. Forecast performance was evaluated using MAE, RMSE, MAPE, and 95% prediction interval coverage. The validation results indicated that the models generally reproduced the broad direction of recent trends, although predictive performance varied across measures. We have added the validation approach to the Methods section and summarized the corresponding results in the Results section, with the detailed results provided in the Supplementary Material.

We have also expanded the Discussion and Limitations to explicitly acknowledge that IBD epidemiology in Asia may still be evolving and that simple time-series models may not fully capture structural transitions in disease patterns. In addition, we note that alternative scenario modeling, such as incorporating future changes in diagnostic capacity or access to biologic therapies, was beyond the scope of the present study and should be addressed in future research.

Comment 3: **The manuscript interprets increases as true disease burden increases, but these trends may partly reflect expansion in colonoscopy availability, specialist care, and awareness.**

Response: Thank you for this important comment. We agree that the observed increases in IBD incidence and prevalence in Asia should not be interpreted solely as reflecting a true increase in disease occurrence. In the revised Discussion, we have clarified that part of the increase may also reflect improved case ascertainment over time, including expanded access to colonoscopy, a growing gastroenterology workforce, greater clinician awareness, and improved diagnostic capacity. We have also noted that the high incidence observed in South Asia may partly reflect improved detection in countries such as India. The revised manuscript now avoids implying that all observed increases necessarily represent true epidemiological change alone.

Comment 4: **The correlation analysis between SDI and IBD burden is superficial; Pearson correlation may not be sufficient, multiple comparisons were not addressed, and causality should not be implied.**

Response: Thank you for this important comment. We agree that the SDI analysis should be interpreted more cautiously. In the revised manuscript, we have avoided causal language and now describe these findings as cross-sectional associations rather than evidence that higher SDI leads to higher IBD rates. To strengthen the analysis, we repeated the SDI-related analyses using Spearman correlation and after excluding high-uncertainty countries. The overall pattern for prevalence remained broadly consistent, whereas the associations for incidence, DALYs, and deaths were weaker and less stable. We have also noted in the Discussion and Limitations that cross-sectional correlations cannot establish temporality or causality.

Comment 5: **The finding that UC DALY rates decrease while CD DALY rates increase is important but insufficiently explored.**

Response: Thank you for this important and insightful comment. We agree that the divergent trends observed for UC and CD deserve more detailed interpretation. In the revised Discussion, we have expanded the discussion of the decline in UC DALY rates and the increase in CD DALY rates, noting that these patterns may reflect differences in disease course, diagnostic practices, and access to effective treatment, including biologic therapies. We have also added text to acknowledge that geographic variation in the UC:CD ratio may be informative and that changes in this ratio over time within countries warrant further investigation. Because the present study was primarily designed to characterize the burden of IBD in Asia at the regional level, a detailed country-specific longitudinal analysis of UC:CD ratios was beyond the scope of the current manuscript and is now identified as an important direction for future research.

Comment 6. Limited integration with clinical realities of IBD care in Asia

Comment: The manuscript lacks discussion of biologic access, COVID-19 disruption, intestinal tuberculosis misdiagnosis, and healthcare system structure.

Response: Thank you for this important comment. We agree that the original manuscript did not adequately reflect the clinical realities of IBD care in Asia. We have therefore expanded the Discussion to address regional differences in access to biologic therapies, specialist and endoscopic care, the possible impact of COVID-19 on IBD care, the challenge of differentiating Crohn’s disease from intestinal tuberculosis in endemic settings, and the influence of healthcare system factors such as access and insurance coverage.

Comment 7: **The finding that males have higher DALY and mortality rates deserves deeper interpretation and, if possible, stratification by region and subtype.**

Response: Thank you for this important and insightful comment. We agree that the higher DALY and mortality rates observed in males deserve deeper analysis. In response, we conducted additional stratified analyses of age-standardized DALY and mortality rates by Asian subregion and disease subtype (IBD, UC, and CD), and the results are now provided in S22 Table. These analyses showed that the magnitude and direction of sex differences varied across regions and between subtypes. Although males generally had higher DALY and mortality rates for IBD overall, this pattern was not uniform across all settings, and the sex disparity was less consistent for CD. We have therefore revised the Discussion to interpret these findings more cautiously and to consider the possible contributions of biological differences, healthcare-seeking behavior, delayed diagnosis, and sociocultural factors affecting access to care.

Comment 9: **The bimodal peak in CD prevalence (40-44 and 60-64 years) is mentioned but not explained. Age-specific incidence and biological/clinical implications should be discussed.**

Response: Thank you for this important and insightful comment. We agree that the bimodal peak in CD prevalence deserves clearer interpretation. Age-specific incidence curves were already included in the manuscript (Fig. 1), together with the age-specific prevalence, DALY, and mortality patterns; however, we acknowledge that this was not sufficiently highlighted or discussed in the original version. In the revised manuscript, we have clarified that the bimodal pattern was observed in the prevalence curve and have expanded the Discussion to consider possible explanations, including differences in disease course, cohort effects, improved survival, and age-related accumulation of prevalent cases. We have also noted that this pattern should not be overinterpreted as evidence of distinct disease phenotypes in the absence of phenotype-specific data. In addition, we now clarify that whether this bimodal pattern is uniform across all regions remains uncertain and warrants further region-specific investigation.

Comment 10: **The resolution of the manuscript images is too low and does not meet the 300 DPI requirement.**

Response: Thank you for pointing this out. We have replaced the original low-resolution images with revised high-resolution figures that meet the journal’s technical requirements, including a resolution of at least 300 DPI.

We thank the Academic Editor and Reviewers again for their careful evaluation and valuable suggestions. We believe that the revisions made in response to these comments have significantly improved the manuscript. We hope that the revised version is now suitable for consideration for publication in *PLOS ONE*.

Sincerely,
Juan Huang
On behalf of all authors.
