# Supplementary material for: The burden of inflammatory bowel disease in Asia (1990–2023) and projections to 2050: A comprehensive analysis of the 2023 global burden of disease study
Source: PLoS One. 2026 Oct 8;21(10):e0359856. doi: 10.1371/journal.pone.0359856 (PMC13649031; doi:10.1371/journal.pone.0359856)
Supplement: Supplementary file 3 [file pone.0359856.s005.docx]

Response to Reviewers

**Manuscript ID: PONE-D-26-15368R1**

*The Burden of Inflammatory Bowel Disease in Asia (1990–2023) and Projections to 2050: A Comprehensive Analysis of the 2023 Global Burden of Disease Study*

Dear Academic Editor and Reviewers,

Thank you for your careful evaluation of our manuscript. We have addressed each comment and revised the manuscript and Supporting Information accordingly. The comments are reproduced below, followed by our point-by-point responses.

# Additional Editor Comments

## Comment 1

GBD uncertainty: Please explain more clearly how limited surveillance data and GBD modelling uncertainty may affect country- and regional-level estimates, particularly in data-sparse Asian countries.

**Response:** Thank you for this important comment. We agree that the implications of limited primary surveillance data and GBD modelling uncertainty were not described sufficiently clearly in the previous version. We have therefore revised the Methods, Results, and Discussion.

In the Methods, we now explain that estimates from countries with limited population-based surveillance, disease registries, vital registration, or endoscopic diagnostic capacity depend more heavily on statistical modelling, covariates, and information borrowed across locations and periods. We also provide a more explicit description of our sensitivity analyses. Relative uncertainty was calculated as the width of the 95% UI divided by the point estimate, and countries in the highest quartile for each burden measure were classified as high-uncertainty countries. We repeated selected analyses after excluding these countries and conducted a separate analysis excluding Afghanistan, Myanmar, and the Lao People’s Democratic Republic. In addition, EAPCs were recalculated using the lower and upper bounds of the GBD 95% UIs.

In the Results, we now report that the increasing incidence and prevalence trends remained directionally consistent across these sensitivity analyses, whereas DALY, mortality, and some SDI-related findings were less stable. In the Discussion, we clarify that country-level differences and rankings may partly reflect variation in data availability, diagnostic capacity, and model dependence rather than genuine epidemiological differences alone. We further explain that regional aggregation may reduce random variation but cannot eliminate systematic or correlated modelling errors, and that the reported 95% UIs may not capture all sources of structural uncertainty, underdiagnosis, or disease misclassification. Accordingly, we have adopted a more cautious interpretation of estimates from data-sparse settings.

## Comment 2

Forecasting: The addition of out-of-sample validation is appreciated. However, the numerical MAE, RMSE, MAPE and prediction-interval coverage should be clearly reported, together with model-selection criteria and residual diagnostics. Please also ensure consistent use of ARIMA versus BAPC throughout the manuscript and submission materials.

**Response:** Thank you for this important comment. We have strengthened the forecasting analysis by reporting the selected ARIMA specifications and AICc values, residual diagnostics using the Ljung–Box test, and numerical out-of-sample validation results, including MAE, RMSE, MAPE, and 95% prediction-interval coverage, in the Results and Supplementary Table S20. The models were trained using data from 1990–2019 and validated against estimates from 2020–2023. All final-model Ljung–Box P values were greater than 0.05, although prediction-interval coverage was limited for some prevalence and mortality series, which is now acknowledged in the Discussion. We also reviewed the manuscript and submission materials and standardized the forecasting terminology throughout to ARIMA, removing all references to BAPC.

## Comment 3

SDI analysis: Please report effect estimates, 95% CIs and model fit more clearly, and address potential non-linearity, multiple testing and the limitations of causal interpretation.

**Response:** Thank you for this important comment. We clarified that the SDI analyses used separate country-level ecological linear regression models with SDI as the sole explanatory variable. The Results now report the regression coefficients per 0.1-unit increase in SDI, 95% CIs, P values, FDR-adjusted P values, R², adjusted R², and AIC, with complete results provided in Supplementary Table S17. Potential non-linearity was assessed using natural cubic spline models, and multiple comparisons were addressed using the Benjamini–Hochberg false discovery rate procedure. We also clarified that these cross-sectional ecological associations cannot be interpreted causally and may be influenced by unmeasured differences in diagnostic capacity, healthcare access, treatment availability, survival, and other country-level factors.

## Comment 4

Interpretation of incidence trends: The distinction between true epidemiological increases and improved diagnosis/case ascertainment should be further emphasized. Importantly, population growth should not be used to explain changes in age-standardized incidence rates.

**Response:** Thank you for this important comment. We have further emphasized that the observed increases in age-standardized incidence may reflect both genuine epidemiological changes and improvements in diagnostic capacity, clinical awareness, endoscopic access, case ascertainment, and reporting. We also removed population growth and population ageing as explanations for changes in age-standardized incidence rates and explicitly clarified that these demographic factors cannot account for temporal changes in age-standardized measures.

## Comment 5

Regional/subtype analyses: The additional sex-stratified analyses are helpful. Further analyses should assess temporal UC/CD differences and whether the reported bimodal CD prevalence pattern is consistent across Asian subregions.

**Response:** Thank you for this important comment. We performed additional analyses comparing temporal trends in UC and CD from 1990 to 2023 using log-linear models with a calendar year × disease subtype interaction term, with Benjamini–Hochberg FDR correction. At the Asia-wide level, age-standardized incidence and prevalence trends did not differ significantly between UC and CD, whereas the temporal trends in age-standardized DALY and mortality rates differed significantly, with UC declining and CD increasing; patterns varied across subregions. We also examined age-specific CD prevalence rates separately across Asia and its five subregions in 2023. The apparent bimodal pattern was reproduced only in East Asia and was not consistently observed in the other subregions. These results are reported in Supplementary Table S22 and Supplementary Figures S4–S5, and the corresponding Methods, Results, and Discussion sections have been revised accordingly.

## Comment 6

Asia-specific context: The Discussion should better address intestinal tuberculosis as a differential diagnosis for CD, regional inequalities in access to biologics/advanced therapies, and the possible impact of COVID-19 on IBD diagnosis and care.

**Response:** Thank you for this important suggestion. We expanded the Discussion to address three Asia-specific considerations. First, we discuss the potential diagnostic overlap between Crohn’s disease and intestinal tuberculosis in endemic settings. Second, we emphasize regional inequalities in access to biologics, advanced therapies, specialist services, and therapeutic monitoring. Third, we discuss how disruptions to endoscopy, outpatient follow-up, infusion services, and treatment continuity during the COVID-19 pandemic may have affected IBD diagnosis and care. We also clarify that these effects could not be directly quantified using the GBD data.

## Comment 7

Projection uncertainty and reproducibility: Please clarify whether projection intervals reflect GBD uncertainty, ARIMA forecast uncertainty, or both. The reported 2050 UC mortality rate of 2.262 should also be checked, as it appears internally inconsistent. The Data Availability Statement should provide the exact GBD query strategy and preferably the analysis code.

**Response:** Thank you for this important comment. We clarified that the reported 95% prediction intervals reflect ARIMA forecast uncertainty conditional on the historical GBD point-estimate series. The GBD uncertainty bounds were not propagated through the forecasting models; therefore, these intervals do not represent GBD uncertainty or combined GBD and ARIMA uncertainty. We also rechecked the 2050 UC mortality projection. The correct value is 0.255 per 100,000 population (95% PI, 0.199–0.311), and this value has been used consistently in the revised manuscript, Fig 7, and S15 Table. Finally, we expanded the Availability of data and materials statement and provided the analytical datasets (Datasheet1.csv–Datasheet4.csv), data descriptions, exact GBD query strategy, complete R code, and Supplementary Appendix to improve reproducibility.

## Comment 8

Please claim the importance of healthcare utilisation and diagnostic delay in IBD, and it would support a more cautious interpretation of increasing incidence as potentially reflecting changes in case detection and healthcare access (PMID: 38802264). Also, the improvements in diagnostic technology and disease assessment can influence observed epidemiological patterns over time (PMID: 39944363).

**Response:** Thank you for this important suggestion. We expanded the Discussion to emphasise the roles of healthcare utilisation, diagnostic delay, specialist access, and advances in endoscopy, imaging, histopathology, biomarkers, and disease assessment. We also revised the interpretation of increasing incidence to clarify that the observed trends may reflect both genuine epidemiological changes and improvements in case detection, healthcare access, reporting, and diagnostic technology. The revised text now explicitly states that increasing incidence should not be interpreted as a true increase in disease occurrence alone. The two suggested references have been added to support these statements.

## Comment 9

In the discussion, please refer to the expanding use, costs and clinical implications of biologics and other advanced therapies in IBD, which is relevant to the advanced treatment across Asian healthcare systems (PMID: 38341192).

**Response:** Thank you for this important suggestion. We have expanded the Discussion to address the increasing use of biologics, small molecules, and other advanced therapies in IBD. We now discuss their clinical benefits, drug and monitoring costs, potential adverse effects, and decisions regarding treatment continuation or withdrawal. We also emphasize that differences in reimbursement, affordability, specialist capacity, and treatment infrastructure may result in unequal uptake and clinical benefit across Asian healthcare systems. The recommended reference (PMID: 38341192) has been added.

## Comment 10

Please discuss the substantial temporal reductions in IBD surgery following the introduction of biologic therapy, highlighting how changes in treatment availability may influence long-term disease burden and outcomes (PMID: 40541271).

**Response:** Thank you for this important suggestion. We have added a concise discussion of the substantial reduction in IBD-related surgery following the introduction of biologic therapy and clarified that differences in treatment availability may contribute to variation in long-term outcomes across Asian healthcare systems.

## Comment 11

Please highlight the heterogeneity of IBD pathogenesis and treatment response and discuss regional and socioeconomic variation in implementation of precision medicine, providing useful context for interpreting heterogeneous IBD trends across Asia (PMID: 39944752).

**Response:** Thank you for this important suggestion. We have expanded the Discussion to highlight the heterogeneity of IBD pathogenesis and treatment response. We also discussed that precision medicine requires molecular testing, specialist expertise, healthcare infrastructure, and financial resources, which are unevenly available across Asian healthcare systems and may contribute to regional differences in treatment response and long-term outcomes.

# Reviewer 1

## Comment 1

Thank you for your responses to my review—the corresponding changes to your manuscript have, in my opinion, improved it. I am satisfied with your response.

**Response:** We sincerely thank the reviewer for the positive assessment of our revisions and for the constructive comments provided during the review process.

# Reviewer 3

## Comment 1

The limitations of GBD-based estimates are now acknowledged, but the revision remains largely descriptive. For countries with limited surveillance data, the reliability of the estimates should be discussed more specifically, and the authors should clarify how uncertainty from GBD modeling affects the country- and regional-level results.

**Response:** Thank you for this important comment. We have clarified the reliability of estimates from countries with limited surveillance and now identify specific data-limited settings with relatively wide uncertainty. We also report the results of sensitivity analyses based on the GBD uncertainty bounds, exclusion of high-uncertainty countries, and separate exclusion of Afghanistan, Myanmar, and Lao PDR. These analyses showed that the increasing incidence and prevalence trends were generally robust, whereas DALY and mortality trends were less stable. We further clarified that uncertainty from data-sparse countries is carried into subregional estimates and that aggregation cannot eliminate systematic uncertainty arising from underdiagnosis, misclassification, or shared modelling assumptions. S19 Table has also been revised to report country-level relative uncertainty and the high-uncertainty classification more clearly.

## Comment 2

The forecasting analysis remains insufficiently validated. The manuscript does not report model selection criteria, residual diagnostics, or out-of-sample validation metrics such as MAE, RMSE, or MAPE. In addition, the Methods describe an ARIMA model, whereas the Discussion refers to a Bayesian age-period-cohort (BAPC) model. This inconsistency should be corrected and the forecasting method clearly described.

**Response:** Thank you for this important comment. We have clarified that all projections were generated using separate non-seasonal ARIMA models. Model selection was based on the corrected Akaike information criterion (AICc), and model adequacy was assessed using residual plots, autocorrelation functions, and the Ljung–Box test. We have added the numerical MAE, RMSE, MAPE, and 95% prediction-interval coverage values to the Results and Supplementary Table S20. We also corrected the terminology throughout the manuscript so that the forecasting method is consistently described as ARIMA rather than BAPC. The limitations of the relatively short validation period and imperfect prediction-interval coverage are now explicitly acknowledged.

## Comment 3

The SDI analysis still requires improvement. Although multivariable linear regression is mentioned in the Methods, the corresponding regression coefficients, confidence intervals, model fit, and covariates are not clearly reported in the Results. The issues of linearity, multiple comparisons, and causal interpretation of cross-sectional correlations also remain.

**Response:** Thank you for this important comment. We clarified that the SDI analyses used separate country-level ecological linear regression models, with SDI as the sole explanatory variable. The Results now report the regression coefficient per 0.1-unit increase in SDI, 95% CI, FDR-adjusted P value, R², and adjusted R², with complete coefficients, P values, AIC values, and model-fit results provided in Supplementary Table S17. Potential non-linearity was assessed using natural cubic spline models, and multiple comparisons were addressed using the Benjamini–Hochberg false discovery rate procedure. We also clarified that these cross-sectional ecological associations cannot be interpreted causally and may reflect differences in diagnostic capacity, healthcare access, treatment availability, survival, and other unmeasured country-level factors.

## Comment 4

The discussion of increasing IBD incidence has improved by acknowledging better diagnosis and healthcare access. However, the manuscript should be more cautious in distinguishing true epidemiologic increases from improved case detection. In particular, population growth should not be used to explain changes in age-standardized incidence rates.

**Response:** Thank you for this important clarification. We revised the Discussion to distinguish more explicitly between genuine epidemiological changes and increases resulting from improved case detection, diagnostic capacity, healthcare utilization, and reporting. We also clarified that population growth and population ageing may increase the absolute number of cases but cannot explain temporal changes in age-standardized incidence rates.

## Comment 5

The authors have expanded the discussion of the different trends in UC and CD, but several requested analyses remain absent, including temporal changes in UC ratios and further stratification by region.

**Response:** Thank you for this important comment. We have now conducted an additional analysis of temporal changes in UC-to-CD ratios for age-standardized incidence, prevalence, DALY, and mortality rates across Asia and its five subregions from 1990 to 2023. The results showed heterogeneous patterns across burden measures and subregions. At the Asian level, the UC-to-CD ratios increased slightly for incidence and prevalence but decreased for DALYs and mortality. We have added the analytical method to the Methods section, summarized the principal findings in the Results and Discussion, and provided the complete results in Supplementary Table S23 and Supplementary Figure S6. The previous statement indicating that longitudinal UC-to-CD ratio analysis was beyond the scope of this study has accordingly been removed.

## Comment 6

The sex-related findings are discussed in more detail, but the requested analyses by both region and disease subtype have not been provided. These subgroup analyses would help determine whether the observed sex differences are consistent across Asia.

**Response:** Thank you for this important comment. We expanded the sex-stratified analyses to include age-standardized incidence, prevalence, DALY, and mortality rates for IBD, UC, and CD across Asia and its five subregions in 2023. Male-to-female ratios were calculated for each region, disease subtype, and burden measure. The results showed that the magnitude and direction of sex differences varied across regions, subtypes, and outcomes rather than following a uniform pattern across Asia. The revised methods and results have been added to the manuscript, and the complete findings are presented in Supplementary Table S24 and Supplementary Figure S7. The Discussion was also revised to emphasize that sex-related differences in IBD burden are context-dependent.

## Comment 7

The explanation of the bimodal prevalence pattern in CD has improved, and age-specific incidence is now presented. However, it remains unclear whether the bimodal pattern is consistent across different Asian regions.

**Response:** Thank you for this important comment. We conducted an additional subregional analysis of age-specific CD prevalence rates in Asia and its five subregions in 2023. The apparent bimodal prevalence pattern was reproduced only in East Asia and was not consistently observed in Southeast Asia, South Asia, Central Asia, or West Asia. We have clarified the analytical approach in the Methods, reported the finding explicitly in the Results, and revised the Discussion to avoid interpreting the aggregate Asian pattern as evidence of universally distinct CD phenotypes. The complete subregional age-specific patterns are presented in Supplementary Figure S5.

## Comment 8

Important Asia-specific clinical issues remain insufficiently addressed, including intestinal tuberculosis as a diagnostic challenge for Crohn’s disease, differences in access to biologics and advanced therapies, and the possible impact of COVID-19 on IBD diagnosis and care during 2020–2023.

**Response:** Thank you for highlighting these important Asia-specific clinical issues. We have reorganized and expanded the Discussion to address them more clearly. We now discuss the diagnostic difficulty caused by the clinical, endoscopic, and histological overlap between Crohn’s disease and intestinal tuberculosis in endemic settings. We also emphasize regional inequalities in access to biologics, other advanced therapies, specialist care, and therapeutic monitoring across Asian healthcare systems. In addition, we discuss how the COVID-19 pandemic may have disrupted endoscopy, outpatient follow-up, infusion services, and treatment continuity during 2020–2023. We further acknowledge that these factors could not be quantified using the GBD data and may have influenced the observed country- and regional-level trends.

## Comment 9

The calculation and interpretation of uncertainty intervals for the projections need clarification. The authors should state whether these intervals represent GBD uncertainty, ARIMA forecast uncertainty, or both. Please also check the reported 2050 UC mortality rate of 2.262, which appears inconsistent with the stated 95% UI of 0.210–0.315.

**Response:** Thank you for identifying this issue. We clarified that the reported 95% intervals for the 2024–2050 projections are ARIMA prediction intervals conditional on the historical GBD point estimates. They represent ARIMA forecast uncertainty only and do not incorporate GBD uncertainty or combined uncertainty from both sources. We also corrected the previously reported 2050 UC mortality rate. The correct projected UC mortality rate is 0.255 per 100,000 population, with a 95% ARIMA prediction interval of 0.199–0.311. The incorrect value of 2.262 and the inconsistent interval have been removed from the manuscript and supplementary materials. We have also standardized the terminology by using “95% prediction interval” for ARIMA forecasts and “95% uncertainty interval” for historical GBD estimates.

## Comment 10

Several minor issues remain. The wording “IBD is increasingly growing in Asia” should be revised, terminology for age-standardized rates should be standardized throughout the manuscript, and the Data Availability Statement should provide sufficient information for reproducibility, including the exact GBD query strategy and, ideally, the analysis code.
Overall, the revision has improved the interpretation of the findings, but the major methodological concerns, particularly regarding forecasting validation, uncertainty, and statistical analysis, remain unresolved. Further major revision is recommended.

**Response:** Thank you for these comments. We revised the phrase “IBD is increasingly growing in Asia” to state that IBD is becoming an increasingly important public health concern in Asia. We also standardized the terminology throughout the manuscript to consistently use “age-standardized incidence rate,” “age-standardized prevalence rate,” “age-standardized DALY rate,” and “age-standardized mortality rate.” The figure and table captions were revised accordingly.

We expanded the Data Availability Statement to provide the GBD source, query context, causes, measures, metrics, sexes, age groups, years, and geographic coverage. The complete query specification, analytical datasets, variable descriptions, and R scripts required to reproduce the analyses, projections, tables, and figures are provided in the Supporting Information file “IBD data and R-code.”

We also addressed the major methodological concerns raised in this review. Specifically, we added ARIMA model-selection criteria, residual diagnostics, numerical out-of-sample validation metrics, and prediction-interval coverage; clarified the distinction between GBD uncertainty intervals and ARIMA prediction intervals; conducted sensitivity analyses for GBD uncertainty; reported the SDI regression estimates, confidence intervals, model-fit statistics, non-linearity tests, and FDR-adjusted results; and added the requested regional, disease-subtype, UC-to-CD ratio, and sex-stratified analyses. The limitations of these analyses are now explicitly acknowledged in the Discussion.
