# Supplementary material for: The burden of inflammatory bowel disease in Asia (1990–2023) and projections to 2050: A comprehensive analysis of the 2023 global burden of disease study
Source: PLoS One. 2026 Oct 8;21(10):e0359856. doi: 10.1371/journal.pone.0359856 (PMC13649031; doi:10.1371/journal.pone.0359856)
Supplement: Peer review history

# Peer review: Decision Letter 0

**DOI:** 10.1371/journal.pone.0359856.r001

**Authors:** Raja Singh Paulraj

1 Jun 2026

Dear Dr. Chen,

Thank you for submitting your manuscript to PLOS ONE. After careful consideration, we feel that it has merit but does not fully meet PLOS ONE’s publication criteria as it currently stands. Therefore, we invite you to submit a revised version of the manuscript that addresses the points raised during the review process.

Please submit your revised manuscript by Jul 16 2026 11:59PM. If you will need more time than this to complete your revisions, please reply to this message or contact the journal office at plosone@plos.org. When you're ready to submit your revision, log on to https://www.editorialmanager.com/pone/ and select the 'Submissions Needing Revision' folder to locate your manuscript file.

- A letter that responds to each point raised by the academic editor and reviewer(s). You should upload this letter as a separate file labeled 'Response to Reviewers'.
- A marked-up copy of your manuscript that highlights changes made to the original version. You should upload this as a separate file labeled 'Revised Manuscript with Track Changes'.
- An unmarked version of your revised paper without tracked changes. You should upload this as a separate file labeled 'Manuscript'.

If you would like to make changes to your financial disclosure, please include your updated statement in your cover letter. Guidelines for resubmitting your figure files are available below the reviewer comments at the end of this letter.

If applicable, we recommend that you deposit your laboratory protocols in protocols.io to enhance the reproducibility of your results. Protocols.io assigns your protocol its own identifier (DOI) so that it can be cited independently in the future. For instructions see: https://journals.plos.org/plosone/s/submission-guidelines#loc-laboratory-protocols. Additionally, PLOS ONE offers an option for publishing peer-reviewed Lab Protocol articles, which describe protocols hosted on protocols.io. Read more information on sharing protocols at https://plos.org/protocols?utm_medium=editorial-email&utm_source=authorletters&utm_campaign=protocols.

As the corresponding author, your ORCID iD is verified in the submission system and will appear in the published article. PLOS supports the use of ORCID, and we encourage all coauthors to register for an ORCID iD and use it as well. Please encourage your coauthors to verify their ORCID iD within the submission system before final acceptance, as unverified ORCID iDs will not appear in the published article. *Only* the individual author can complete the verification step; PLOS staff *cannot* verify ORCID iDs on behalf of authors.

We look forward to receiving your revised manuscript.

Kind regards,

Raja Singh Paulraj, Ph.D.

Academic Editor

PLOS One

Journal Requirements:

When submitting your revision, we need you to address these additional requirements.

1.Please ensure that your manuscript meets PLOS ONE's style requirements, including those for file naming. The PLOS ONE style templates can be found at

https://journals.plos.org/plosone/s/file?id=wjVg/PLOSOne_formatting_sample_main_body.pdf and

https://journals.plos.org/plosone/s/file?id=ba62/PLOSOne_formatting_sample_title_authors_affiliations.pdf

2. Please note that PLOS One has specific guidelines on code sharing for submissions in which author-generated code underpins the findings in the manuscript. In these cases, we expect all author-generated code to be made available without restrictions upon publication of the work. Please review our guidelines at https://journals.plos.org/plosone/s/materials-and-software-sharing#loc-sharing-code and ensure that your code is shared in a way that follows best practice and facilitates reproducibility and reuse.

3. Please note that funding information should not appear in any section or other areas of your manuscript. We will only publish funding information present in the Funding Statement section of the online submission form. Please remove any funding-related text from the manuscript.

4. We note that the grant information you provided in the ‘Funding Information’ and ‘Financial Disclosure’ sections do not match.

When you resubmit, please ensure that you provide the correct grant numbers for the awards you received for your study in the ‘Funding Information’ section.

5. Thank you for stating the following financial disclosure:

“National Natural Science Foundation of China(82575056);Clinical Research Center for Traditional Chinese Medicine on Anorectal and Perianal Wound Repair of Fujian Province (X202502)”

Please state what role the funders took in the study.  If the funders had no role, please state: "The funders had no role in study design, data collection and analysis, decision to publish, or preparation of the manuscript."

If this statement is not correct you must amend it as needed.

Please include this amended Role of Funder statement in your cover letter; we will change the online submission form on your behalf.

6. We noted in your submission details that a portion of your manuscript may have been presented or published elsewhere. Please clarify whether this [conference proceeding or publication] was peer-reviewed and formally published. If this work was previously peer-reviewed and published, in the cover letter please provide the reason that this work does not constitute dual publication and should be included in the current manuscript.

7. We note that Figure 3, Appendix Figure 1 and Appendix Figure 2 in your submission contain map images which may be copyrighted. All PLOS content is published under the Creative Commons Attribution License (CC BY 4.0), which means that the manuscript, images, and Supporting Information files will be freely available online, and any third party is permitted to access, download, copy, distribute, and use these materials in any way, even commercially, with proper attribution. For these reasons, we cannot publish previously copyrighted maps or satellite images created using proprietary data, such as Google software (Google Maps, Street View, and Earth). For more information, see our copyright guidelines: http://journals.plos.org/plosone/s/licenses-and-copyright.

We require you to either (1) present written permission from the copyright holder to publish these figures specifically under the CC BY 4.0 license, or (2) remove the figures from your submission:

1. You may seek permission from the original copyright holder of Figure 3, Appendix Figure 1 and Appendix Figure 2 to publish the content specifically under the CC BY 4.0 license.

We recommend that you contact the original copyright holder with the Content Permission Form (http://journals.plos.org/plosone/s/file?id=7c09/content-permission-form.pdf) and the following text:

“I request permission for the open-access journal PLOS ONE to publish XXX under the Creative Commons Attribution License (CCAL) CC BY 4.0 (http://creativecommons.org/licenses/by/4.0/). Please be aware that this license allows unrestricted use and distribution, even commercially, by third parties. Please reply and provide explicit written permission to publish XXX under a CC BY license and complete the attached form.”

Please upload the completed Content Permission Form or other proof of granted permissions as an "Other" file with your submission.

In the figure caption of the copyrighted figure, please include the following text: “Reprinted from [ref] under a CC BY license, with permission from [name of publisher], original copyright [original copyright year].”

2. If you are unable to obtain permission from the original copyright holder to publish these figures under the CC BY 4.0 license or if the copyright holder’s requirements are incompatible with the CC BY 4.0 license, please either i) remove the figure or ii) supply a replacement figure that complies with the CC BY 4.0 license. Please check copyright information on all replacement figures and update the figure caption with source information. If applicable, please specify in the figure caption text when a figure is similar but not identical to the original image and is therefore for illustrative purposes only.

The following resources for replacing copyrighted map figures may be helpful:

USGS National Map Viewer (public domain): http://viewer.nationalmap.gov/viewer/

The Gateway to Astronaut Photography of Earth (public domain): http://eol.jsc.nasa.gov/sseop/clickmap/

Maps at the CIA (public domain): https://www.cia.gov/library/publications/the-world-factbook/index.html and https://www.cia.gov/library/publications/cia-maps-publications/index.html

NASA Earth Observatory (public domain): http://earthobservatory.nasa.gov/

Landsat: http://landsat.visibleearth.nasa.gov/

USGS EROS (Earth Resources Observatory and Science (EROS) Center) (public domain): http://eros.usgs.gov/#

Natural Earth (public domain): http://www.naturalearthdata.com/

8. Please include your tables as part of your main manuscript and remove the individual files. Please note that supplementary tables (should remain/ be uploaded) as separate "supporting information" files.

9. Please include captions for your Supporting Information files at the end of your manuscript, and update any in-text citations to match accordingly. Please see our Supporting Information guidelines for more information: http://journals.plos.org/plosone/s/supporting-information.

10. We note that there is identifying data in the Supporting Information file Data Sheet 1.csv and Data Sheet 2.csv. Due to the inclusion of these potentially identifying data, we have removed this file from your file inventory. Prior to sharing human research participant data, authors should consult with an ethics committee to ensure data are shared in accordance with participant consent and all applicable local laws.

Data sharing should never compromise participant privacy. It is therefore not appropriate to publicly share personally identifiable data on human research participants. The following are examples of data that should not be shared:

-Name, initials, physical address

-Ages more specific than whole numbers

-Internet protocol (IP) address

-Specific dates (birth dates, death dates, examination dates, etc.)

-Contact information such as phone number or email address

-Location data

-ID numbers that seem specific (long numbers, include initials, titled “Hospital ID”) rather than random (small numbers in numerical order)

Data that are not directly identifying may also be inappropriate to share, as in combination they can become identifying. For example, data collected from a small group of participants, vulnerable populations, or private groups should not be shared if they involve indirect identifiers (such as sex, ethnicity, location, etc.) that may risk the identification of study participants.

Additional guidance on preparing raw data for publication can be found in our Data Policy (https://journals.plos.org/plosone/s/data-availability#loc-human-research-participant-data-and-other-sensitive-data) and in the following article: http://www.bmj.com/content/340/bmj.c181.long.

Please remove or anonymize all personal information (<specific identifying information in file to be removed>), ensure that the data shared are in accordance with participant consent, and re-upload a fully anonymized data set. Please note that spreadsheet columns with personal information must be removed and not hidden as all hidden columns will appear in the published file.

11. If the reviewer comments include a recommendation to cite specific previously published works, please review and evaluate these publications to determine whether they are relevant and should be cited. There is no requirement to cite these works unless the editor has indicated otherwise.

Additional Editor Comments:

Thank you for submitting your manuscript.

After consideration of the reviewer comments and my own assessment, I find that the manuscript addresses an important topic and has potential for publication. However, several issues must be addressed before the manuscript can be further considered.

Please carefully address Reviewer 3's methodological concerns regarding the limitations of GBD-based estimates, justification and validation of the ARIMA forecasting model, interpretation of temporal trends, and the discussion of SDI-related findings. In particular, the Discussion should more clearly acknowledge the uncertainty associated with model-derived estimates and avoid causal interpretations of correlation analyses.

In addition, please strengthen the discussion of regional differences in IBD burden, healthcare infrastructure, diagnostic capacity, and access to advanced therapies across Asia. The age-, sex-, and disease subtype-specific findings should also be further interpreted.

Reviewer 1's suggestions regarding presentation of results, comparison with global trends, and discussion of factors such as smoking and diagnostic awareness are encouraged and should be considered where appropriate.

Finally, please improve the overall presentation of the manuscript, including language editing, figure quality, and readability of the Results section.

I believe the concerns raised are addressable through revision; therefore, the manuscript is invited for Major Revision. Please provide a detailed point-by-point response to all reviewer and editor comments.

Minor Issues

1. Improve the overall English language and grammatical consistency throughout the manuscript.

2. Increase the resolution and quality of all figures to meet journal requirements.

3. Expand discussion regarding age- and sex-specific findings.

4. Clarify several speculative interpretations and ensure conclusions remain supported by the presented data.

Editorial Assessment

The manuscript has merit and may become suitable for publication after substantial revision. The authors are encouraged to provide a detailed point-by-point response addressing all reviewer and editorial comments.

Editorial Decision: Major Revision

Sincerely,

Academic Editor

Reviewers' comments:

Reviewer's Responses to Questions

**Comments to the Author**

1. Is the manuscript technically sound, and do the data support the conclusions?

Reviewer #1: Yes

Reviewer #2: Yes

Reviewer #3: Partly

**********

2. Has the statistical analysis been performed appropriately and rigorously?

Reviewer #1: I Don't Know

Reviewer #2: Yes

Reviewer #3: I Don't Know

**********

3. Have the authors made all data underlying the findings in their manuscript fully available?

The PLOS Data policy

Reviewer #1: Yes

Reviewer #2: Yes

Reviewer #3: Yes

**********

4. Is the manuscript presented in an intelligible fashion and written in standard English?

Reviewer #1: Yes

Reviewer #2: Yes

Reviewer #3: No

**********

Reviewer #1: Interesting paper of potential importance.

1. My main comment is about presentation - the results section is very difficult to read currently. I would suggest much of the numerical data that's there could be moved to tables which would help alot.

2. I would also suggest authors consider including some published trend data from other parts of the world over this time period to suggest if the changes are 'worldwide'.

3. They could also speculate more if the trends are due to true jncreaees in rates or just more diagnoses being made.

4. What about population cigarette smoking rates over this time- a major association with IBD?

Reviewer #2: I hope in following studies you can include in an extensive way the contribution of diet, nutrition and other environmental or social factors to this phenomena. A reasonable proposal to change the diseases course and preferably, a deep critical public health system to change the onset of the disease.

Reviewer #3: This manuscript presents a comprehensive analysis of IBD burden across 47 Asian countries from 1990-2023 using GBD 2023 data, with projections to 2050. The authors report increasing incidence and prevalence with declining DALY rates, and identify significant regional heterogeneity. While the scope is ambitious and the topic timely, several methodological and interpretative concerns limit the manuscript's impact.

1.The authors rely entirely on GBD 2023 modeled estimates without acknowledging the significant limitations of this approach for IBD in Asia.GBD estimates for IBD in low- and middle-income countries are heavily model-dependent due to sparse primary data. The manuscript does not discuss how GBD handles countries with minimal or no IBD surveillance systems. No sensitivity analyses are presented to assess the reliability of estimates, particularly for countries like Afghanistan, Myanmar, or Lao PDR where health data infrastructure is limited. The authors state data gaps are "filled using statistical modeling techniques" but provide no detail on the uncertainty introduced by this process.

2.The ARIMA model assumes future trends follow historical patterns, which is problematic for IBD.IBD incidence in Asia is in an acceleration phase (Kaplan's Stage 2-3), whereas Western countries have reached compounding prevalence (Stage 4). Simple time-series models cannot capture epidemiologic transitions.The manuscript provides no validation of ARIMA model performance (e.g., out-of-sample prediction accuracy for 2020-2023).No alternative scenarios are presented (e.g., what if diagnostic capacity improves dramatically, or if biologic access expands?).

3.The manuscript interprets all increases as true disease burden increases, but Asia has seen dramatic expansion of colonoscopy availability, GI specialist workforce, and awareness over this period.South Asia's highest "incidence rate" may partly reflect better detection in countries like India with expanding healthcare infrastructure.

4. The correlation analysis between SDI and IBD burden is superficial. Pearson correlation assumes linear relationships, which may not apply here. No adjustment for multiple comparisons despite numerous correlations tested. The interpretation that "higher SDI leads to higher IBD rates" oversimplifies complex causal pathways. Cross-sectional correlations cannot establish temporality

5. The observation that UC DALY rates decrease while CD rates increase is critically important but inadequately explored. Could this reflect differential treatment advances (biologics benefit CD more?) or changing diagnostic patterns? The geographic variation in UC:CD ratios deserves detailed analysis, does this ratio change over time within countries?

6. The manuscript lacks integration with clinical realities of IBD care in Asia. No discussion of biologic drug availability/access across regions. No mention of the impact of COVID-19 on IBD care (data span includes 2020-2023).No consideration of the role of intestinal tuberculosis misdiagnosis in CD diagnosis in endemic areas.Healthcare system factors (public vs. private care, insurance coverage) are not addressed

7.The finding that males have higher DALY and mortality rates deserves deeper analysis. Is this specific to certain countries/regions? How do gender disparities vary by disease subtype?

Could this reflect healthcare-seeking behavior differences or biological differences? The authors should stratify gender analyses by region and subtype; discuss cultural factors affecting healthcare access for women.

9. The bimodal peak in CD prevalence (40-44 and 60-64) is mentioned but not explained. Could this reflect two different disease phenotypes? Is this pattern consistent across all regions? Does this bimodal pattern exist in incidence or only prevalence? The authors should provide age-specific incidence curves (not just prevalence) and discuss biological/clinical implications.

10.The resolution of the images in the manuscript is too low and does not meet the 300 DPI requirement.

**********

what does this mean?). If published, this will include your full peer review and any attached files.

If you choose “no”, your identity will remain anonymous but your review may still be made public.

**Do you want your identity to be public for this peer review?** For information about this choice, including consent withdrawal, please see our Privacy Policy

Reviewer #1: No

Reviewer #2: **Yes:**  Sophia E Martinez Vazquez

Reviewer #3: No

**********

[NOTE: If reviewer comments were submitted as an attachment file, they will be attached to this email and accessible via the submission site. Please log into your account, locate the manuscript record, and check for the action link "View Attachments". If this link does not appear, there are no attachment files.]

To ensure your figures meet our technical requirements, please review our figure guidelines: https://journals.plos.org/plosone/s/figures

You may also use PLOS’s free figure tool, NAAS, to help you prepare publication quality figures: https://journals.plos.org/plosone/s/figures#loc-tools-for-figure-preparation.

NAAS will assess whether your figures meet our technical requirements by comparing each figure against our figure specifications.

# Author comment: Author response to Decision Letter 1

**DOI:** 10.1371/journal.pone.0359856.r002

4 Jul 2026

Thank you for the careful and constructive comments from the Academic Editor and Reviewers. We have addressed all editor and reviewer comments in detail and uploaded a separate “Response to Reviewers” file containing our point-by-point responses. In the revised manuscript, we have clarified the limitations of the GBD-based estimates, added sensitivity analyses, strengthened the validation and interpretation of the ARIMA projections, revised the discussion of SDI-related findings to avoid causal language, expanded the interpretation of regional, age-, sex-, and subtype-specific results, improved the presentation and readability of the Results section, and updated the figures, supplementary materials, and data/code availability information accordingly.

Attachment Submitted filename: Response to Reviewers.docx

# Peer review: Decision Letter 1

**DOI:** 10.1371/journal.pone.0359856.r003

**Authors:** Lanlan Chen

24 Aug 2026

Dear Dr. Chen,

Thank you for submitting your manuscript to PLOS One. After careful consideration, we feel that it has merit but does not fully meet PLOS One’s publication criteria as it currently stands. Therefore, we invite you to submit a revised version of the manuscript that addresses the points raised during the review process.

Please submit your revised manuscript by Oct 08 2026 11:59PM. If you will need more time than this to complete your revisions, please reply to this message or contact the journal office at plosone@plos.org. When you're ready to submit your revision, log on to https://www.editorialmanager.com/pone/ and select the 'Submissions Needing Revision' folder to locate your manuscript file.

- A letter that responds to each point raised by the academic editor and reviewer(s). You should upload this letter as a separate file labeled 'Response to Reviewers'.
- A marked-up copy of your manuscript that highlights changes made to the original version. You should upload this as a separate file labeled 'Revised Manuscript with Track Changes'.
- An unmarked version of your revised paper without tracked changes. You should upload this as a separate file labeled 'Manuscript'.

If you would like to make changes to your financial disclosure, please include your updated statement in your cover letter. Guidelines for resubmitting your figure files are available below the reviewer comments at the end of this letter.

If applicable, we recommend that you deposit your laboratory protocols in protocols.io to enhance the reproducibility of your results. Protocols.io assigns your protocol its own identifier (DOI) so that it can be cited independently in the future. For instructions see: https://journals.plos.org/plosone/s/submission-guidelines#loc-laboratory-protocols. Additionally, PLOS One offers an option for publishing peer-reviewed Lab Protocol articles, which describe protocols hosted on protocols.io. Read more information on sharing protocols at https://plos.org/protocols?utm_medium=editorial-email&utm_source=authorletters&utm_campaign=protocols.

As the corresponding author, your ORCID iD is verified in the submission system and will appear in the published article. PLOS supports the use of ORCID, and we encourage all coauthors to register for an ORCID iD and use it as well. Please encourage your coauthors to verify their ORCID iD within the submission system before final acceptance, as unverified ORCID iDs will not appear in the published article. *Only* the individual author can complete the verification step; PLOS staff *cannot* verify ORCID iDs on behalf of authors.

We look forward to receiving your revised manuscript.

Kind regards,

Lanlan Chen

Academic Editor

PLOS One

Journal Requirements:

1. If the reviewer comments include a recommendation to cite specific previously published works, please review and evaluate these publications to determine whether they are relevant and should be cited. There is no requirement to cite these works unless the editor has indicated otherwise.

Additional Editor Comments:

The revision has improved the manuscript, but several important methodological issues remain.

1. GBD uncertainty: Please explain more clearly how limited surveillance data and GBD modelling uncertainty may affect country- and regional-level estimates, particularly in data-sparse Asian countries.

2. Forecasting: The addition of out-of-sample validation is appreciated. However, the numerical MAE, RMSE, MAPE and prediction-interval coverage should be clearly reported, together with model-selection criteria and residual diagnostics. Please also ensure consistent use of ARIMA versus BAPC throughout the manuscript and submission materials.

3. SDI analysis: Please report effect estimates, 95% CIs and model fit more clearly, and address potential non-linearity, multiple testing and the limitations of causal interpretation.

4. Interpretation of incidence trends: The distinction between true epidemiological increases and improved diagnosis/case ascertainment should be further emphasized. Importantly, population growth should not be used to explain changes in age-standardized incidence rates.

5. Regional/subtype analyses: The additional sex-stratified analyses are helpful. Further analyses should assess temporal UC/CD differences and whether the reported bimodal CD prevalence pattern is consistent across Asian subregions.

6. Asia-specific context: The Discussion should better address intestinal tuberculosis as a differential diagnosis for CD, regional inequalities in access to biologics/advanced therapies, and the possible impact of COVID-19 on IBD diagnosis and care.

7. Projection uncertainty and reproducibility: Please clarify whether projection intervals reflect GBD uncertainty, ARIMA forecast uncertainty, or both. The reported 2050 UC mortality rate of 2.262 should also be checked, as it appears internally inconsistent. The Data Availability Statement should provide the exact GBD query strategy and preferably the analysis code.

8. Please claim the importance of healthcare utilisation and diagnostic delay in IBD, and it would support a more cautious interpretation of increasing incidence as potentially reflecting changes in case detection and healthcare access (PMID: 38802264). Also, the improvements in diagnostic technology and disease assessment can influence observed epidemiological patterns over time (PMID: 39944363).

9. In the discussion, please refer to the expanding use, costs and clinical implications of biologics and other advanced therapies in IBD, which is relevant to the advanced treatment across Asian healthcare systems (PMID: 38341192).

10. Please discuss the substantial temporal reductions in IBD surgery following the introduction of biologic therapy, highlighting how changes in treatment availability may influence long-term disease burden and outcomes (PMID: 40541271).

11. Please highlight the heterogeneity of IBD pathogenesis and treatment response and discuss regional and socioeconomic variation in implementation of precision medicine, providing useful context for interpreting heterogeneous IBD trends across Asia (PMID: 39944752).

[Note: HTML markup is below. Please do not edit.]

Reviewer's Responses to Questions

**Comments to the Author**

Reviewer #1: All comments have been addressed

Reviewer #3: (No Response)

**********

2. Is the manuscript technically sound, and do the data support the conclusions?

Reviewer #1: Yes

Reviewer #3: Partly

**********

3. Has the statistical analysis been performed appropriately and rigorously?

Reviewer #1: I Don't Know

Reviewer #3: I Don't Know

**********

4. Have the authors made all data underlying the findings in their manuscript fully available?

The PLOS Data policy

Reviewer #1: Yes

Reviewer #3: No

**********

5. Is the manuscript presented in an intelligible fashion and written in standard English?

Reviewer #1: Yes

Reviewer #3: Yes

**********

Reviewer #1: Thank you for your responses to my review - the corresponding changes to your manuscript has, in my opinion, improved it. I am satisfied with your response.

Reviewer #3: 1.The limitations of GBD-based estimates are now acknowledged, but the revision remains largely descriptive. For countries with limited surveillance data, the reliability of the estimates should be discussed more specifically, and the authors should clarify how uncertainty from GBD modeling affects the country- and regional-level results.

2.The forecasting analysis remains insufficiently validated. The manuscript does not report model selection criteria, residual diagnostics, or out-of-sample validation metrics such as MAE, RMSE, or MAPE. In addition, the Methods describe an ARIMA model, whereas the Discussion refers to a Bayesian age-period-cohort (BAPC) model. This inconsistency should be corrected and the forecasting method clearly described.

3.The SDI analysis still requires improvement. Although multivariable linear regression is mentioned in the Methods, the corresponding regression coefficients, confidence intervals, model fit, and covariates are not clearly reported in the Results. The issues of linearity, multiple comparisons, and causal interpretation of cross-sectional correlations also remain.

4.The discussion of increasing IBD incidence has improved by acknowledging better diagnosis and healthcare access. However, the manuscript should be more cautious in distinguishing true epidemiologic increases from improved case detection. In particular, population growth should not be used to explain changes in age-standardized incidence rates.

5.The authors have expanded the discussion of the different trends in UC and CD, but several requested analyses remain absent, including temporal changes in UC ratios and further stratification by region.

6.The sex-related findings are discussed in more detail, but the requested analyses by both region and disease subtype have not been provided. These subgroup analyses would help determine whether the observed sex differences are consistent across Asia.

7.The explanation of the bimodal prevalence pattern in CD has improved, and age-specific incidence is now presented. However, it remains unclear whether the bimodal pattern is consistent across different Asian regions.

8.Important Asia-specific clinical issues remain insufficiently addressed, including intestinal tuberculosis as a diagnostic challenge for Crohn’s disease, differences in access to biologics and advanced therapies, and the possible impact of COVID-19 on IBD diagnosis and care during 2020–2023.

9.The calculation and interpretation of uncertainty intervals for the projections need clarification. The authors should state whether these intervals represent GBD uncertainty, ARIMA forecast uncertainty, or both. Please also check the reported 2050 UC mortality rate of 2.262, which appears inconsistent with the stated 95% UI of 0.210–0.315.

10.Several minor issues remain. The wording “IBD is increasingly growing in Asia” should be revised, terminology for age-standardized rates should be standardized throughout the manuscript, and the Data Availability Statement should provide sufficient information for reproducibility, including the exact GBD query strategy and, ideally, the analysis code.

Overall, the revision has improved the interpretation of the findings, but the major methodological concerns, particularly regarding forecasting validation, uncertainty, and statistical analysis, remain unresolved. Further major revision is recommended.

**********

what does this mean?). If published, this will include your full peer review and any attached files.

If you choose “no”, your identity will remain anonymous but your review may still be made public.

**Do you want your identity to be public for this peer review?** For information about this choice, including consent withdrawal, please see our Privacy Policy

Reviewer #1: No

Reviewer #3: No

**********

[NOTE: If reviewer comments were submitted as an attachment file, they will be attached to this email and accessible via the submission site. Please log into your account, locate the manuscript record, and check for the action link "View Attachments". If this link does not appear, there are no attachment files.]

To ensure your figures meet our technical requirements, please review our figure guidelines: https://journals.plos.org/plosone/s/figures

You may also use PLOS’s free figure tool, NAAS, to help you prepare publication quality figures: https://journals.plos.org/plosone/s/figures#loc-tools-for-figure-preparation.

NAAS will assess whether your figures meet our technical requirements by comparing each figure against our figure specifications.

# Author comment: Author response to Decision Letter 2

**DOI:** 10.1371/journal.pone.0359856.r004

8 Sep 2026

We have addressed all editor and reviewer comments. A detailed point-by-point response is provided in the uploaded “Response to Reviewers” file, and all revisions are highlighted in the tracked-changes manuscript.

Attachment Submitted filename: Response_to_Reviewers_auresp_2.docx

# Editor report: Decision Letter 2

**DOI:** 10.1371/journal.pone.0359856.r005

**Authors:** Lanlan Chen

21 Sep 2026

The burden of inflammatory bowel disease in Asia (1990-2023) and projections to 2050: a comprehensive analysis of the 2023 global burden of disease study

PONE-D-26-15368R2

Dear Dr. Chen,

We’re pleased to inform you that your manuscript has been judged scientifically suitable for publication and will be formally accepted for publication once it meets all outstanding technical requirements.

Within one week, you’ll receive an e-mail detailing the required amendments. When these have been addressed, you’ll receive a formal acceptance letter and your manuscript will be scheduled for publication.

An invoice will be generated when your article is formally accepted. Please note, if your institution has a publishing partnership with PLOS and your article meets the relevant criteria, all or part of your publication costs will be covered. Please make sure your user information is up-to-date by logging into Editorial Manager at Editorial Manager® and clicking the ‘Update My Information' link at the top of the page. For questions related to billing, please contact billing support.

If your institution or institutions have a press office, please notify them about your upcoming paper to help maximize its impact. If they’ll be preparing press materials, please inform our press team as soon as possible -- no later than 48 hours after receiving the formal acceptance. Your manuscript will remain under strict press embargo until 2 pm Eastern Time on the date of publication. For more information, please contact onepress@plos.org.

Kind regards,

Lanlan Chen

Academic Editor

PLOS One

Additional Editor Comments (optional):

Reviewers' comments:

# Editor report: Acceptance letter

**DOI:** 10.1371/journal.pone.0359856.r006

**Authors:** Lanlan Chen

PONE-D-26-15368R2

PLOS One

Dear Dr. Chen,

I'm pleased to inform you that your manuscript has been deemed suitable for publication in PLOS One. Congratulations! Your manuscript is now being handed over to our production team.

At this stage, our production department will prepare your paper for publication. This includes ensuring the following:

* All references, tables, and figures are properly cited

* All relevant supporting information is included in the manuscript submission,

* There are no issues that prevent the paper from being properly typeset

You will receive further instructions from the production team, including instructions on how to review your proof when it is ready. Please keep in mind that we are working through a large volume of accepted articles, so please give us a few days to review your paper and let you know the next and final steps.

Lastly, if your institution or institutions have a press office, please let them know about your upcoming paper now to help maximize its impact. If they'll be preparing press materials, please inform our press team within the next 48 hours. Your manuscript will remain under strict press embargo until 2 pm Eastern Time on the date of publication. For more information, please contact onepress@plos.org.

You will receive an invoice from PLOS for your publication fee after your manuscript has reached the completed accept phase. If you receive an email requesting payment before acceptance or for any other service, this may be a phishing scheme. Learn how to identify phishing emails and protect your accounts at https://explore.plos.org/phishing.

If we can help with anything else, please email us at customercare@plos.org.

Thank you for submitting your work to PLOS One and supporting open access.

Kind regards,

PLOS One Editorial Office Staff

on behalf of

M.D Lanlan Chen

Academic Editor

PLOS One
